# Synthesis, Moleculer Modeling, and Carbonic Anhydrase Inhibitor Effects of Novel 1‐(1‐benzyl‐2‐oxoindolin‐3‐ylidene)‐4‐(3‐sulfamoylphenyl)thiosemicarbazides

**DOI:** 10.1002/cmdc.70467

**Published:** 2026-10-12

**Authors:** Çağla Begüm Apaydın, Tuğçe Cinek, Hayrunnisa Nadaroğlu, Nilgün Karalı

**Affiliations:** ^1^ Department of Pharmaceutical Chemistry Faculty of Pharmacy Istanbul University Istanbul Turkey; ^2^ Department of Pharmaceutical Chemistry Faculty of Pharmacy Istanbul Health and Technology University Istanbul Turkey; ^3^ Department of Food Technology Vocational College of Technical Sciences Atatürk University Erzurum Turkey

**Keywords:** 2‐oxoindoline, carbonic anhydrase, molecular docking, sulfonamide, thiosemicarbazide

## Abstract

In this study, we describe the synthesis of new 1‐(1‐benzyl‐2‐oxoindolin‐3‐ylidene)‐4‐(3‐sulfamoylphenyl)thiosemicarbazides and evaluate their inhibitory activity against hCAs (**6a–l**). The inhibitory effects of compounds **6a–l** were examined in vitro against hCA I, II, IX, and XII isoforms. Acetazolamide (AAZ) was employed as the standard reference compound in this study. The compounds **6a–l** inhibited the tumor‐associated hCA IX and XII isoenzymes at nanomolar levels, and showed weak inhibitory effects on the off‐target hCA I and II isoenzymes. Selectivity index (SI) values of compounds **6a–l** were determined to be significantly high for hCA IX and XII over hCA I and II compared to AAZ. Compound **6k** (R_1_ = Cl, R_2_ = 3‐Cl) showed the strongest inhibitory effect (*K*_*i*_ = 25.23 nM) and high selectivity (SI = 225, 185) against hCA IX over hCA I and II. Compound **6f** (R_1_ = CH_3_, R_2_ = 4‐Cl) was found to have the highest inhibitory effect (*K*_*i*_ = 36.65 nM) and high selectivity (SI = 377 and 192, respectively) against hCA XII over hCA I and II. Molecular docking and dynamic simulation analyses were conducted to explore how compounds **6f** and **6k** interact with active sites of hCA XII and IX enzymes.

## Graphical abstract

Novel 1‐(1‐benzyl‐2‐oxoindolin‐3‐ylidene)thiosemicarbazides (**6a–l**) bearing a 3‐sulfamoylphenyl moiety selectively inhibited the tumor‐associated hCA IX and XII isoforms over hCA I and II isoforms at the nanomolar levels. Compounds **6k** and **6f** emerged as the most potent inhibitors of hCA IX and XII, respectively. Molecular modeling studies also supported the stable binding of compounds **6k** and **6f** to the active sites of the target enzymes.

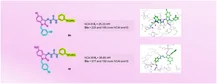

## 1 Introduction

Carbonic anhydrases (CAs; EC 4.2.1.1) are a large family of metalloenzymes that facilitate the reversible interconversion of carbon dioxide (CO_2_), bicarbonate (HCO_3_^−^), and protons (H^+^), thereby regulating their balance within biological systems [1]. The active site of most CAs contains a zinc ion (Zn^2+^), which is essential for their catalytic function. The enzymatic activity of CAs is essential for numerous physiological and pathological functions. These functions encompass the exchange of CO_2_ and bicarbonate between active tissues and the lungs, regulation of pH and CO_2_ levels, electrolyte secretions in different organs, participation in biosynthetic pathways including gluconeogenesis, urea formation, and lipogenesis, and involvement in processes including bone remodeling, calcification, and cancer development [2].

Altered expression of human CAs (hCAs) has been associated with the pathophysiology of numerous disorders, including cancer, hypertension, glaucoma, epilepsy, and neuropathic pain. Among the different hCA isoforms, the transmembrane isoenzymes hCA IX and XII are overexpressed in many solid tumors and contribute to tumor progression by regulating extracellular pH, cell proliferation, adhesion, and invasion. Consequently, these tumor‐associated isoenzymes have emerged as attractive targets for the development of anticancer agents [3, 4].

Sulfonamide‐containing drugs functioning as hCAIs have been widely used for many years in the treatment of conditions such as glaucoma and edema, including acetazolamide (AAZ), brinzolamide, dichlorophenamide, dorzolamide, ethoxzolamide, and methazolamide. However, sulfonamide‐based hCAIs, defined as first‐ or second‐generation, have many negative side effects because they inhibit most of the hCA isoenzymes found in mammals and have low selectivity [5] (Figure 1).

**FIGURE 1:**
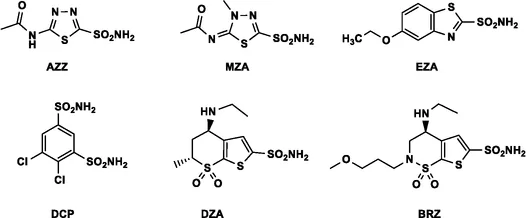
Clinically used sulfonamide hCAIs.

Benzenesulfonamides are widely represented among hCAIs, and their interactions with the enzyme have been extensively studied. These compounds serve as versatile scaffolds that can be efficiently modified to achieve isoenzyme specificity. It has been reported that 4‐sulfamoylphenylthiourea derivatives (**I** and **II**, Figure 2), developed to achieve a balanced hydrophilic/lipophilic profile with the aim of improving selectivity and reducing side effects, exhibit different CA inhibition profiles depending on the nature of the R substituent. Several derivatives have shown significant inhibitory activity against hCA I, II, and XII isoenzymes in the low nanomolar range [6, 7, 8, 9]. Likewise, ureido‐substituted benzenesulfonamides (**III**) have been reported to display diverse isoform selectivity depending on the substituent pattern, with several derivatives exhibiting potent inhibition of hCA IX and XII at low nanomolar concentrations, while some compounds also demonstrated significant anticancer activity in both in vitro and in vivo studies [10, 11, 12, 13, 14].

**FIGURE 2:**
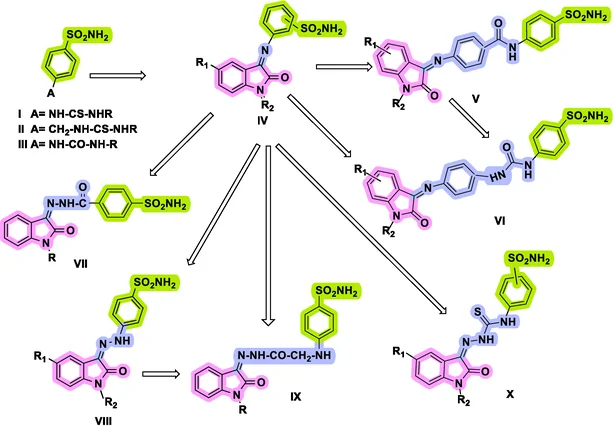
Development of 2‐oxoindoline derivatives bearing sulfamoylphenyl moiety as hCAIs.

In recent years, numerous studies have been conducted to develop benzene‐sulfonamide conjugates bearing an 2‐oxoindoline core structure as selective hCAIs. 3‐Imino‐2‐oxoindoline‐substituted benzenesulfonamides (**IV**) were found to be selective inhibitors against hCA IX and XII. The highest inhibitory effect was observed in 3‐ and 4‐benzenesulfonamides, and the effect decreased significantly in 2‐benzenesulfonamides. Among 4‐benzenesulfonamide derivatives, the methyl and nitro‐substituted compounds at R_1_ were found to be the most effective derivatives against hCA IX and XII isoenzymes, respectively [15] (Figure 2).

The newly developed amido/ureido‐substituted benzenesulfonamide‐oxindole conjugates (**V** and **VI**) exhibited significant, nonselective inhibitory effects against hCA isoenzymes at nanomolar concentrations [16]. However, it was determined that the selective inhibitory effects of 1‐alkyl/benzyl‐2‐oxoindoline conjugates (**VI**), which enhance hydrophobic tail interactions within the hCA IX active site‐were significantly increased compared to their 1‐unsubstituted derivatives [17]. Hydrazono‐2‐oxoindoline‐based benzenesulfonamide derivatives (**VII**) were found to inhibit hCA IX and XII isoenzymes at low nanomolar levels. In that study, the 1‐ethyl‐substituted compound was identified as having the highest inhibitory activity against hCA IX and XII [18]. Synthesized 3‐hydrazono‐2‐oxoindolines bearing an N‐(4‐sulfamoylphenyl) group (**VIII**) and those bearing an N‐(4‐sulfamoylphenyl)acetamide group (**IX**) displayed significant inhibition profiles against various hCA isoforms. While the unsubstituted compounds VIII and IX showed significant inhibitory effects against hCA II and IX at low nanomolar levels, it was found that ethyl‐ and benzyl‐substituted **IX** compounds significantly enhanced selective inhibitory activity against hCA IX. Furthermore, for both series (**VIII** and **IX**), the presence of a benzoyl group on the 2‐oxoindoline ring was determined to enhance selective activity against hCA XII [19, 20] (Figure 2).

In one of our previous studies, we evaluated the inhibitory effects of compounds (**X**) containing a 4‐benzenesulfonamide group against hCA I and II, as well as hCA IX and hCA XII. The compounds exhibited potent, selective inhibitory effects against hCA XII and hCA IX at low nanomolar concentrations. While substitution at the 5‐position of the 2‐oxoindoline ring enhanced inhibitory activity and selectivity—particularly against hCA XII‐compounds bearing a fluorine or chlorine group at the 7‐position showed reduced inhibitory effects and selectivity against hCA XII [21]. In another of our studies, compounds (**X**) with a 3‐benzenesulfonamide substituent demonstrated higher inhibitory activities against the hCA II isoenzyme compared to other hCA isoenzymes at nanomolar levels. Overall, it was determined that compounds bearing methyl, ethyl, or benzyl substituents at the 1‐position of the 2‐oxoindoline ring exhibited stronger inhibitory effects than their unsubstituted derivatives. However, a decrease in selectivity against hCA II was observed alongside increased activity against hCA IX and XII isoenzymes, particularly in compounds bearing a chlorine group at the 5‐position and ethyl and benzyl substituents at the 1‐position of the 2‐oxoindoline ring [22] (Figure 2).

Collectively, these studies illustrate a chronological improvement in the design of 2‐oxoindoline‐based hCAIs—from micromolar to low‐nanomolar inhibition‐achieved through strategic variation of linkers, substitution patterns, and tail‐group modifications to enhance isoform selectivity and therapeutic potential. It shows that the ethyl, benzyl, and benzoyl moieties located in the 1‐position of the 2‐oxoindoline ring play an important role in increasing the selective effect against tumor‐based hCA IX and XII isoenzymes. In this study, which is a continuation of our previous studies, we aimed to examine the inhibitory effects of 2‐oxoindolines bearing methyl‐, fluorine‐, and chlorine‐substituted benzyl moiety at the 1‐position of the 2‐oxoindoline ring against hCA I, II, IX, and XII isozymes.

## 2 Results and Discussion

### 2.1 Chemistry

1‐Benzyl‐1*H*‐indole‐2,3‐diones (**2a–l**) were synthesized from the reaction of 1*H*‐indole‐2,3‐diones (**1a,b**) with benzyl chlorides [23, 24]. 3‐(Sulfamoylphenyl)isothiocyanate (**4**) was obtained by reacting 3‐aminobenzenesulfonamide (**3**) with thiophosgene [25]. Subsequently, 4‐(3‐sulfamoylphenyl)thiosemicarbazide (**5**) was prepared from the reaction of compound **4** with hydrazine hydrate [24, 26]. Finally, 1‐(1‐benzyl‐2‐oxoindolin‐3‐ylidene)‐4‐(3‐sulfamoylphenyl)thiosemicarbazides (**6a–l**) were obtained via the condensation of compound **5** with compounds **2a–l** (Scheme 1). The structures of all newly synthesized compounds (**6a–l**) were confirmed using elemental analysis and spectroscopic techniques (IR, ^1^H NMR, and ^13^C NMR‐APT).

**SCHEME 1:**
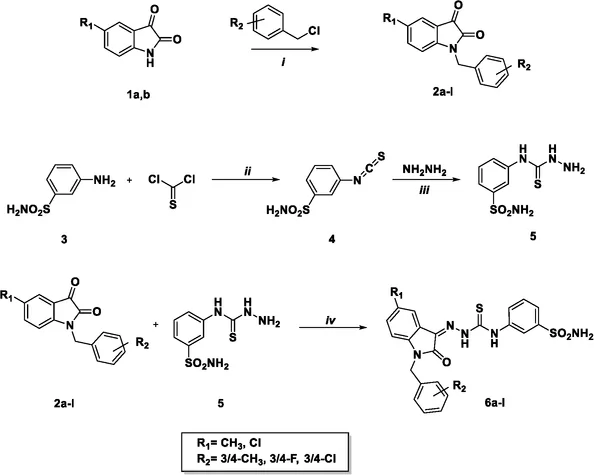
Synthesis of compounds **6a–l**. Reagents and conditions: (i) DMF, K_2_CO_3_, KI, stirred, 50–60 °C, 5–6 h, (ii) H_2_O, HCl, stirred, (iii) EtOH, stirred, cooled, and (iv) EtOH, H_2_SO_4_, reflux.

The IR spectra of compounds **6a–l** showed absorption bands between 3398 and 3165 cm^−1^, corresponding to the N=H stretching vibrations of the thioamide, lactam, and sulfonamide groups. Besides the absorption bands defining the lactam C=O (1787–1782 cm^−1^) and thioamide C=S (1125–1117 cm^−1^) groups, the S=O stretching vibrations of the sulfonamide groups exhibited two distinct absorption bands at 1322–1315 and 1156–1149 cm^−1^. In the ^1^H NMR spectra of compounds **6a–l**, the thioamide protons at N_2_ and N_4_ appeared as two separate singlets, resonating at *δ* 12.81–12.58 and *δ* 11.14–11.08 ppm regions, respectively. Additionally, a singlet was detected at *δ* 7.49–7.40 ppm region, attributable to the sulfonamide NH protons. Moreover, the CH_2_ protons of the benzyl group gave rise to a singlet at *δ* 5.03–4.93 ppm region. Carbon signals were assigned based on ^13^C NMR‐APT spectral data. The presence of C=S (*δ* 177.05–177.01 ppm region) and C=O (*δ* 161.45–161.12 ppm region) resonances in the ^13^C NMR‐APT spectra verified the thiosemicarbazone structure of compounds **6a–l**. The splitting of indole carbon resonances into doublets in compounds **6c**, **6d**, **6i**, and **6j** was attributed to ^13^C‐^19^F coupling. For 3‐fluorobenzyl‐substituted thiosemicarbazone derivatives (**6c** and **6i**), the benzyl C_3_ carbon resonated more downfield at *δ* 162.80–162.78 ppm compared to the indole C_2_ carbon (*δ* 161.44–161.20 ppm region) resulting from the fluorine atom's deshielding influence, and showed as a doublet with a coupling constant of 242.5 Hz. For the 4‐fluorobenzyl‐substituted thiosemicarbazone derivatives (**6d** and **6j**), the C_4_ carbon of the benzyl group exhibited a downfield shift to *δ* 162.06–162.05 ppm relative to the indole C_2_ carbon (*δ* 161.38–161.14 ppm), owing to the fluorine atom's electron‐withdrawing properties, and appeared as a doublet with a coupling constant of 242.5 Hz.

### 2.2 CA Inhibitory Activity

The inhibitory effects of compounds **6a–l** against hCA I, II, IX, and XII isoenzymes were evaluated using a modified Wilbur‐Anderson spectrophotometric assay [27, 28], with AAZ as the reference. IC_50_ values were determined at varying inhibitor concentrations while keeping the substrate constant, defining enzyme activity without inhibitors as 100%. Residual activities were used to generate activity (%) versus inhibitor concentration ([I]) plots for IC_50_ calculation. Inhibition constants (*K* *_i_*) were derived from Lineweaver–Burk plots, and average *K* *_i_* values were reported for each compound [27, 28, 29]. The IC_50_ and *K* *_i_* results for **6a–l** are summarized in Tables 1 and 2 for hCA I/II and hCA IX/XII, respectively.

**TABLE 1:** The results obtained from regression analysis graphs for hCA I and II of the compounds **6a–l**.

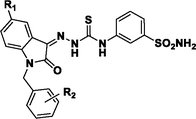								
Compound	**R**_**1**_	**R**_**2**_	hCA I, μM			hCA II, μM		
			**IC**_**50**_	***R*** ^**2**^	***K*** *_**i**_*	**IC**_**50**_	***R*** ^**2**^	***K*** *_**i**_*
**6a**	CH_3_	3‐CH_3_	7.63	0.9920	6.996 ± 0.84	9.39	0.9644	7.76 ± 0.93
**6b**	CH_3_	4‐CH_3_	10.78	0.9948	8.12 ± 0.97	7.45	0.9917	6.22 ± 0.75
**6c**	CH_3_	3‐F	4.73	0.9331	3.24 ± 0.39	7.43	0.9864	6.17 ± 0.74
**6d**	CH_3_	4‐F	8.76	0.9898	6.497 ± 0.78	8.87	0.9822	7.34 ± 0.88
**6e**	CH_3_	3‐Cl	8.14	0.9942	6.497 ± 1.78	1.17	0.9934	9.12 ± 1.09
**6f**	CH_3_	4‐Cl	17.26	0.9880	13.81 ± 2.66	8.49	0.9833	7.04 ± 0.84
**6g**	Cl	3‐CH_3_	8.23	0.9843	6.497 ± 0.78	2.78	0.9935	2.32 ± 0.28
**6h**	Cl	4‐CH_3_	6.74	0.9854	4.87 ± 0.58	1.06	0.9836	8.63 ± 1.04
**6i**	Cl	3‐F	5.61	0.9982	4.062 ± 0.49	1.10	0.9697	9.02 ± 1.21
**6j**	Cl	4‐F	9.10	0.9892	7.99 ± 0.96	7.17	0.9892	5.13 ± 0.62
**6k**	Cl	3‐Cl	7.69	0.9789	5.685 ± 0.68	3.39	0.9943	4.68 ± 0.56
**6l**	Cl	4‐Cl	8.36	0.9890	9.75 ± 1.17	1.03	0.9646	8.38 ± 1.01
**AAZ**	—	—	0.15	0.9896	0.07 ± 0.14	0.16	0.9952	0.012 ± 0.01

**TABLE 2:** The results obtained from regression analysis graphs for hCA IX and XII of the compounds **6a–l**.

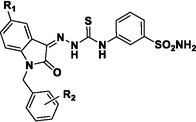								
Compound	**R**_**1**_	**R**_**2**_	hCA IX, nM			hCA XII, nM		
			**IC**_**50**_	***R*** ^**2**^	***K*** *_**i**_*	**IC**_**50**_	***R*** ^**2**^	***K*** *_**i**_*
**6a**	CH_3_	3‐CH_3_	66.27	0.9733	53.84 ± 1.62	66.70	0.9921	51.13 ± 2.53
**6b**	CH_3_	4‐CH_3_	66.67	0.9978	54.15 ± 1.63	51.72	0.9816	39.61 ± 2.19
**6c**	CH_3_	3‐F	62.16	0.9522	50.50 ± 2.52	50.36	0.9536	38.57 ± 1.16
**6d**	CH_3_	4‐F	67.25	0.9884	54.63 ± 3.64	62.06	0.9884	47.58 ± 3.43
**6e**	CH_3_	3‐Cl	49.90	0.9928	40.54 ± 0.22	58.63	0.9863	44.91 ± 2.35
**6f**	CH_3_	4‐Cl	68.56	0.9849	55.71 ± 2.67	47.85	0.9918	36.65 ± 2.10
**6g**	Cl	3‐CH_3_	59.62	0.9967	48.44 ± 2.45	53.34	0.9815	40.84 ± 3.23
**6h**	Cl	4‐CH_3_	48.42	0.9719	39.34 ± 0.18	50.53	0.9768	38.70 ± 0.16
**6i**	Cl	3‐F	36.95	0.9667	30.02 ± 0.90	54.92	0.9868	42.04 ± 2.26
**6j**	Cl	4‐F	50.37	0.9787	40.92 ± 1.23	55.19	0.9773	42.25 ± 3.27
**6k**	Cl	3‐Cl	31.06	0.996	25.23 ± 0.76	50.16	0.9809	38.42 ± 2.15
**6l**	Cl	4‐Cl	49.78	0.9887	40.44 ± 2.21	76.61	0.9928	58.69 ± 3.76
**AAZ**	—	—	38.65	0.9915	46.22 ± 4.22	31.43	0.9911	23.15 ± 2.77

All new compounds **6a–l** inhibited the hCA IX and XII isoenzymes at nanomolar levels (*K* *_i_* = 25.23–55.71 nM and 36.65–58.69 nM, respectively). However, compounds **6a–l** displayed inhibitory effects against hCA I and II isoenzymes at low micromolar levels (*K* *_i_* = 3.24–13.81 and 2.32–9.12 μM, respectively). For this reason, the selectivity index (SI) values of compounds **6a–l** were determined to be significantly high for hCA IX (SIs = 64–248 and 48–300, respectively) and XII (SIs = 84–377 and 57–223, respectively) over hCA I and II. Whereas, the SI values of the standard compound AAZ were found to be extremely low for hCA IX (SIs = 2 and 0.3, respectively) and XII (SIs = 3 and 0.5, respectively) over hCA I and II (Tables 2 and 3).

**TABLE 3:** Selectivity index values of the compounds **6a–l** for hCA IX and XII over hCA I and II.

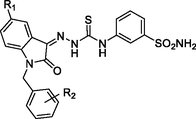						
Compound	**R**_**1**_	**R**_**2**_	SI values for hCA IX		SI values for hCA XII	
			**over** **hCA I**	**over** **hCA II**	**over** **hCA I**	**over** **hCA II**
**6a**	CH_3_	3‐CH_3_	130	144	137	152
**6b**	CH_3_	4‐CH_3_	150	115	205	157
**6c**	CH_3_	3‐F	64	122	84	160
**6d**	CH_3_	4‐F	119	134	137	154
**6e**	CH_3_	3‐Cl	160	225	145	203
**6f**	CH_3_	4‐Cl	248	126	377	192
**6g**	Cl	3‐CH_3_	134	48	159	57
**6h**	Cl	4‐CH_3_	124	219	126	223
**6i**	Cl	3‐F	135	300	97	215
**6j**	Cl	4‐F	195	125	189	121
**6k**	Cl	3‐Cl	225	185	148	122
**6l**	Cl	4‐Cl	241	207	166	143
**AAZ**			2	0.3	3	0.5

Among the tested compounds, compound **6k** (R_1_ = Cl, R_2_ = 3‐Cl) was found to be the strongest inhibitor against hCA IX (*K* *_i_* = 25.23 ± 0.76 nM), and had high selectivity against hCA XII over hCA I and II (SIs = 225 and 185, respectively). Compound **6k** also had an approximately two‐fold higher inhibition value compared to AAZ (*K* *_i_* = 46.22 ± 4.22 nM) and 112‐ and 617‐fold higher selectivity against hCA IX over hCA I and II, respectively. Moreover, compound **6e** (R_1_ = CH_3_, R_2_ = 3‐Cl), compound **6h** (R_1_ = Cl, R_2_ = 4‐CH_3_), compound **6j** (R_1_ = Cl, R_2_ = 4‐F), and compound **6l** (R_1_ = Cl, R_2_ = 4‐Cl), were found to have significantly higher inhibition and SI values compared to AAZ against hCA IX over hCA I and II. *K*_*i*_ values for compounds **6e**, **6h**, **6i**, **6j**, and **6l** were 40.54 ± 0.22, 39.34 ± 0.18, 30.02 ± 0.90, 40.92 ± 1.23, and 40.44 ± 2.21 nM, respectively. SI values for these compounds were 160, 124, 135, 195 and 241 against hCA IX over hCA I and 225, 219, 300, 125, and 207 against hCA IX over hCA II, respectively. All compounds (except **6e**, **6h**, **6i**, **6j**, and **6l**) exhibited high inhibitory activity against hCA IX, comparable to that of AAZ (Tables 2 and 3).

All tested compounds showed lower inhibitory effects than AAZ (*K*_*i*_ = 23.15 ± 2.77 nM) at the nanomolar level against hCA XII. However, the SI values of all compounds were found to be significantly higher compared to AAZ against hCA XII over hCA I and II. Compound **6f** (R_1_ = CH_3_, R_2_ = 4‐Cl), was found to be the highest inhibitory effect against hCA XII (*K*_*i*_ = 36.65 ± 36.65 ± 2.10 nM) and had high selectivity against hCA XII over hCA I and II (SIs = 377 and 192, respectively). Moreover, compound **6b** (R_1_ = CH_3_, R_2_ = 4‐CH_3_), compound **6c** (R_1_ = CH_3_, R_2_ = 3‐F), compound **6h** (R_1_ = Cl, R_2_ = 4‐CH_3_), and compound **6k** (R_1_ = Cl, R_2_ = 3‐Cl) showed high inhibitory effects against hCA XII. *K*_*i*_ values for compounds **6b**, **6c**, **6h**, and **6k** were 39.61 ± 2.19, 38.57 ± 1.16, 38.70 ± 0.16, and 38.42 ± 2.15 nM, respectively. SI values for the compounds were 205, 84, 126, and 148 against hCA XII over hCA I, and 157, 160, 223, and 122 against hCA XII over hCA II, respectively.

In our previous study [22], it was determined that compounds bearing nonsubstituted benzyl ring showed notable inhibitory activity against hCA II, together with a higher selectivity for hCA II compared to hCAs I, IX, and XII. In contrast, in the present study, substitutions introduced on the benzyl ring reduced the inhibitory activity against the off‐target isoforms hCA I and II to the micromolar range, while markedly enhancing the inhibitory potency toward the anticancer target isoforms hCA IX and XII to the nanomolar level, thereby enabling the development of more selective compounds.

Evaluation of the substituent effects at the R_1_ and R_2_ positions of the synthesized compounds **6a–l** revealed that these modifications play a significant role in determining both inhibitory potency and selectivity. Notably, compounds bearing a chlorine substituent at the R_1_ position exhibited higher inhibitory potency against hCA IX and XII isoenzymes compared with their methyl‐substituted analogues. The compounds (**6a–f**) bearing a methyl group at the R_1_ position generally exhibit lower inhibitory effects compared to chlorine‐substituted compounds (**6g–l**). The presence of an electron‐withdrawing a chlorine group at the R_2_ position has been found to increase inhibition against hCA IX and XII. It was found that the SI values of some compounds (**6c**, **6g**, and **6i**) bearing 3‐methyl and 3‐fluorine groups on the R_2_ position were below 100, while all other compounds were above 100.

### 2.3 Molecular Modeling Studies

Molecular modeling studies were performed to elucidate the potential binding modes and interaction profiles of the compounds **6k** (in the active sites of hCA IX) and **6f** (in the active sites of hCA XII). Since compounds **6k** and **6f** were determined to be the strongest inhibitor compounds against hCA IX and XII, respectively, these compounds were used in the modeling studies. Analysis of the docked pose of **6k** revealed that the negatively charged sulfonamide group forms a hydrogen bond (with THR200) and a coordination interaction (with the Zn^2+^ ion in the binding pocket). Another hydrogen bond was observed between TRP9 and the oxindole carbonyl, while a pi–pi stacking interaction was identified between HIS94 and benzenesulfonamide (Figure 3A,B).

**FIGURE 3:**
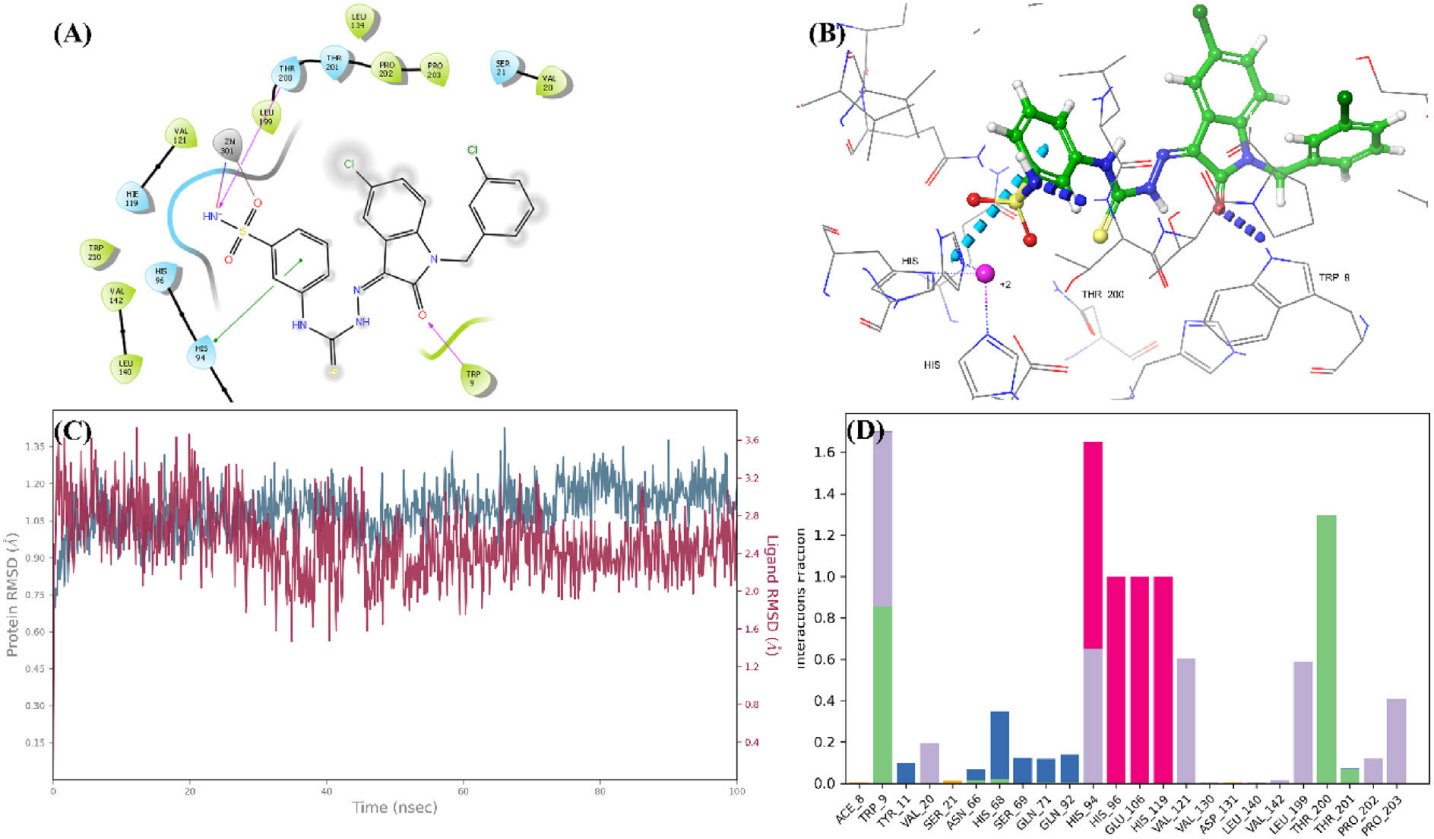
Docking and MD simulation of compound **6k** in hCA IX (PDB ID: 8Q18). (A) The docked pose of compound **6k** in the hCA IX active site (8Q18) (pink arrow shows hydrogen bond, gray line shows metal coordination and green line shows pi–pi stacking interaction). (B) 2D interaction diagram of top‐scored docking pose of compound **6k** (dark blue line shows hydrogen bond, blue/pink dots shows metal coordination and light blue line represents pi–pi stacking interaction). (C) RMSD of the protein Cα‐atoms (blue) and ligand (red). (D) Protein–ligand contacts during the simulation exist more than 30 % of the time during the MD simulation.

The 100 ns molecular dynamics (MD) simulation of **6k**‐hCA IX (PDB ID: 8Q18) complex shows that the protein root–mean‐square deviation (RMSD) rapidly stabilized within the first few nanoseconds and remained consistently around ~1.0–1.2 Å throughout the simulation, indicating that no significant conformational fluctuations occurred in the global protein structure. This stable RMSD profile confirms that the protein maintained its overall fold under the simulation conditions. The ligand RMSD fluctuates within the range of ≈2–3 Å after the initial relaxation period, reflecting stable binding within the active site (Figure 3C). The complex maintains a stable interaction pattern throughout the simulation. The anionic sulfonamide group showed stable coordination with Zn^2+^ ion, which remained conserved for nearly the entire duration of the MD simulation. Additionally, a stable hydrogen bond was formed between the sulfonamide oxygen and the backbone of THR200, persisting for over 90% of simulation. A pi–pi stacking between HIS94 and benzenesulfonamide phenyl ring was maintained for a substantial portion of the simulation (~60%). Additionally, the oxindole carbonyl group formed a persistent hydrogen bond with TRP9 (85%). VAL121, PRO202, and PRO203 formed hydrophobic interactions that contributed to maintaining the binding pose of compound **6k** (Figure 3D).

Analysis of the docked pose of compound **6f** revealed that the negatively charged sulfonamide group forms a hydrogen bond with the THR198 and simultaneously coordinates the active‐site Zn^2+^ ion, a characteristic interaction observed in many potent hCAIs. Also, a hydrogen bond was observed between the carbonyl group of the oxindole moiety and SER130 (Figure 4A,B).

**FIGURE 4:**
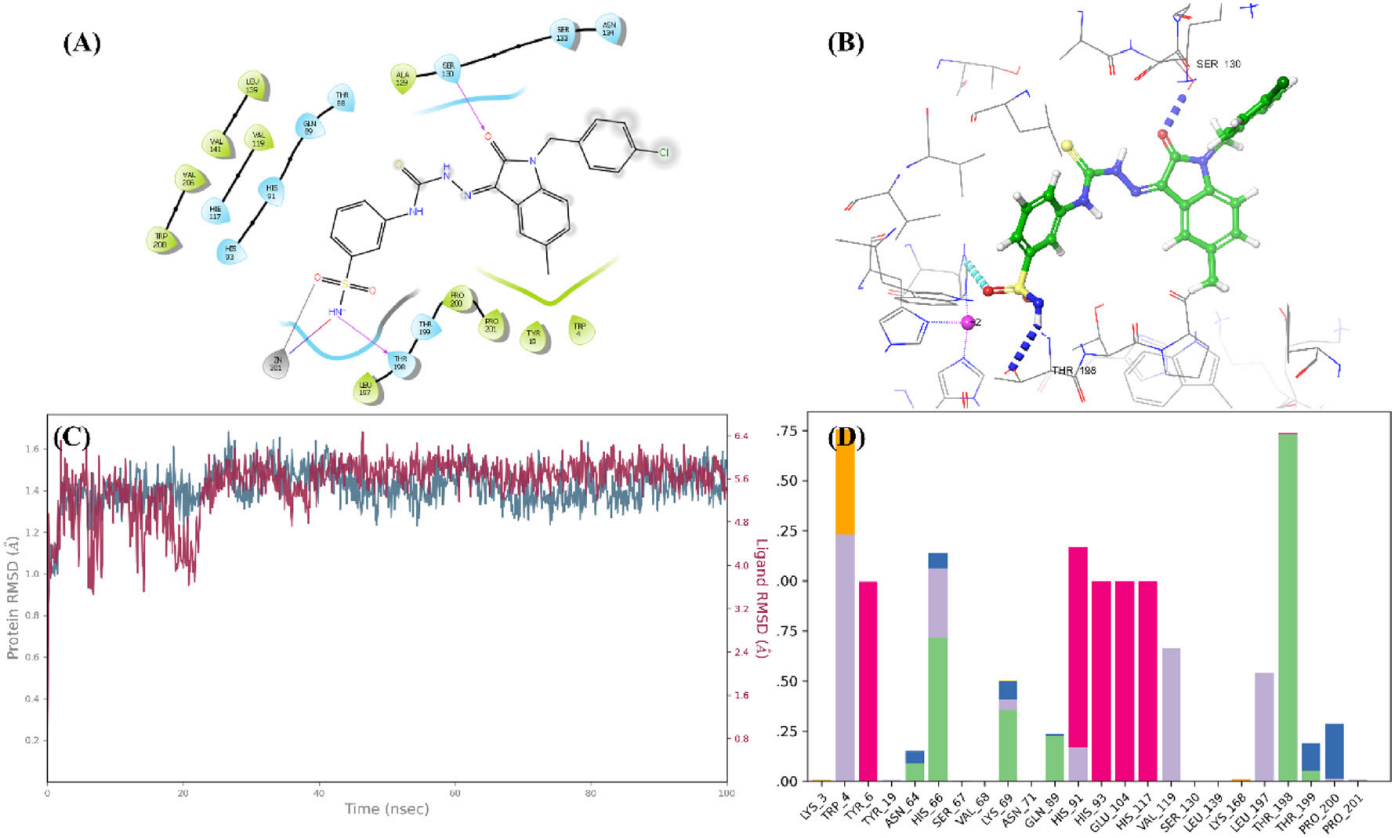
Docking and MD simulation of compound **6f** in hCA XII (PDB ID: 4KP5). (A) The docked pose of compound **6f** in the hCA XII active site (4KP5) (pink arrow shows hydrogen bond, gray line shows metal coordination and green line shows pi–pi stacking interaction). (B) 2D interaction diagram of top‐scored docking pose of compound **6f** (dark blue line shows hydrogen bond, blue/pink dots shows metal coordination and light blue line shows pi–pi stacking interaction). (C) RMSD of the protein Cα‐atoms (blue) and ligand (red). (D) Protein–ligand contacts during the simulation exist more than 30 % of the time during the MD simulation.

Analysis of the 100 ns MD trajectory of **6f**‐hCA XII (PDB ID: 4KP5) complex indicated that the protein backbone displayed only minor fluctuations, reaching equilibrium at an early stage and maintaining the RMSD values within the range of 1.3–1.6 Å during the simulation, as illustrated in the RMSD profile. This behavior indicates that the overall protein structures remained stable over the simulation time. In addition, ligand RMSD showed that the ligand exhibited initial fluctuation of up to ~5 Å with respect to the initial binding pose, followed by stabilization after ≈20 ns, indicating the relatively stable binding conformation (Figure 4C). The complex revealed a stable interaction network between the ligand and the catalytic site, characterized by hydrogen bonding, hydrophobic interactions, and metal coordination (Zn^2+^). The anionic sulfonamide group showed stable coordination with Zn^2+^ ion, which remained (98%) interaction throughout the MD simulation. The hydrogen‐bond interaction with THR198 observed in the docking pose was retained for 74% of the MD simulation. Throughout the simulation, it was observed that the oxindole carbonyl group formed hydrogen‐bond interactions with HIS66 and LYS69, with corresponding occupancies of ~70% and ~35%, respectively. In addition, HIS66 was found to participate in pi–pi stacking with the 4‐chlorophenyl ring (~33% occupancy), while this aromatic moiety also exhibited a pi–pi stacking with TRP4, exhibiting a higher occupancy of ≈66%. Furthermore, TRP4 displayed an additional pi–pi stacking with the phenyl ring of the oxindole scaffold for about 50% of the simulation time (Figure 4D).

### 2.4 In Silico ADME Properties

To evaluate the drug‐likeness of compounds **6a–l**, key physicochemical properties were predicted and assessed in accordance with Lipinski's rule of five (RO5). The predicted pharmacokinetic profiles of compounds **6a–l** indicated that the series exhibits favorable drug‐like characteristics. According to RO5, all compounds were within the acceptable limits, with no more than one allowable violation. Compounds **6a–l** have molecular weights ranging from 493 to 534, along with three hydrogen bond donors and 10 hydrogen bond acceptors. According to in silico predictions, central nervous system (CNS) activity scores for all compounds were −2, indicating CNS‐inactive. Their predicted blood–brain barrier (BBB) partition coefficients were predicted to fall within the range of −2.172 to −1.806, while partition coefficient (logP) values were observed between 3.238 and 3.672. Human serum albumin binding constants (logKhsa) of the compounds were found to be within an acceptable range (0.182–0.283), comparable to the reference values. In silico estimation of oral absorption revealed percentages ranging from 69.63% to 82.26%, indicating moderate to favorable oral absorption potential. Based on RO5, all evaluated physicochemical parameters were within the acceptable range, except for the molecular weight for some compounds [30] (Table 4).

**TABLE 4:** Lipinski's rule of five and predicted pharmacokinetic properties data of compounds **6a–l**.

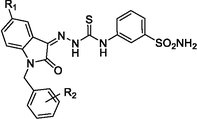											
Cpd.	Molecular weight	Number of hydrogen bond donors		Number of hydrogen bond acceptors	logP octanol / water	Number of RO5 rules not complied with	logBB	CNS	logKhsa	Oral absorption percentage in human	
	Ref. <500	<5		<10	<5	≤1	−3 to 1.2	−2 inactive; +2 active	−1.5 to 1.5	**>80 high;** **<25 weak**	
**6a**	493.597	3		10	3.271	0	−2.204	−2	0.280	81.85	
**6b**	493.597	3		10	3.299	0	−2.172	−2	0.283	82.67	
**6c**	497.561	3		10	3.238	0	−2.086	−2	0.185	81.55	
**6d**	497.561	3		10	3.239	0	−2.024	−2	0.182	82.26	
**6e**	514.016	3		10	3.483	1	−2.040	−2	0.247	70.13	
**6f**	514.016	3		10	3.487	1	−2.001	−2	0.248	70.64	
**6g**	514.016	3		10	3.456	1	−2.007	−2	0.241	70.04	
**6h**	514.016	3		10	3.441	1	−2.008	−2	0.240	69.98	
**6i**	517.979	3		10	3.423	1	−1.901	−2	0.147	69.63	
**6j**	517.979	3		10	3.423	1	−1.827	−2	0.144	70.44	
**6k**	534.434	3		10	3.646	1	−1.827	−2	0.204	71.14	
**6l**	534.434	3		10	3.672	1	−1.806	−2	0.209	71.76	
**AAZ**	222.236	3		9	−1.745	0	−1.741	−2	−0.973	44.81	

Abbreviations: CNS, central nervous system activity; Cpd, compound; logBB, blood–brain barrier partition coefficient; logKhsa, human serum albumin binding ratio; logP, partition coefficient.

## 3 Conclusion

In this study, new 1‐(1‐benzyl‐2‐oxoindolin‐3‐ylidene)thiosemicarbazides bearing 3‐sulfamoylphenyl group were synthesized, and all compounds were found to selectively inhibit cancer‐related transmembrane hCA IX and XII isoenzymes in the nanomolar range. One of the most important outcomes of this study is that the design of halogen‐ and methyl‐substituted benzyl derivatives from derivatives bearing an unsubstituted benzyl scaffold successfully reduced cytosolic hCA I and II inhibition while increasing activity against tumor‐associated hCA IX and XII isoforms, resulting in improved selectivity. Additionally, it was determined that the SI values of all compounds were above 100, excluding some 3‐methyl and 3‐fluorine benzyl substituted compounds against hCA IX and XII isoforms over the off‐target hCA I and II isoforms. It was determined that the compound **6k** bearing chlorine groups at the 5‐position of the oxindole ring and the 3‐position of the benzyl residue showed the strongest inhibitory effect against the hCA IX isoenzyme. The strongest inhibitor compound against hCA XII isoenzyme was found to be compound **6f** bearing a methyl group at the 5‐position of the oxindole ring and a chlorine group at the 4‐position of the benzyl moiety. Molecular modeling of the most potent inhibitor compounds **6k** and **6f** revealed that π–π stacking interactions involving the benzyl moiety contribute to their enhanced potency. The results obtained show that these compounds can be considered as promising leads for the optimization of compounds with therapeutic potential with planned cellular cytotoxicity tests and mechanism of action determinations.

## 4 Experimental

### 4.1 Chemistry

All reagents used were purchased from Sigma–Aldrich or Merck (Germany). Melting points were recorded using open capillary tubes on a Buchi B‐540 apparatus and are reported without correction. Infrared (IR) spectra were collected as KBr pellets on a Shimadzu IR Affinity‐1 FTIR spectrometer. ^1^H and ^13^C NMR spectra were recorded in DMSO‐d_6_ using Varian UNITY INOVA 500 MHz and Varian Mercury (Agilent) 400 MHz spectrometers. Elemental analyses (C, H, N) were performed using a Leco CHNS 932 analyzer. (ind., indole; benz., benzyl; ph., phenyl).

#### 4.1.1 Synthesis of 1‐(1‐benzyl‐2‐oxoindolin‐3‐ylidene)‐4‐(3‐sulfamoylphenyl)thiosemicarbazides (6a–l)

Compounds **6a–l** were obtained from 4‐(3‐sulfamoylphenyl)thiosemicarbazide (**5**) (3.5 mmol) and 1‐benzyl‐1*H*‐indole‐2,3‐diones (**2a–l**) (3.5 mmol) in ethanol with catalytic sulfuric acid under reflux, following our previously reported procedure [21, 22]. The obtained product was purified by recrystallization from ethanol.

##### 4.1.1.1 1‐(3‐methylbenzyl‐5‐methyl‐2‐oxoindolin‐3‐ylidene)‐4‐(3‐sulfamoylphenyl)thiosemicarbazide (6a)

Yellow powder (86%): m.p.: 228–233 °C. **IR (KBr) *ν***_**maks**_ **(cm** ^**−1**^ **)**: 3332, 3257, 3165 (N=H), 1689 (C=O), 1622, 1597, 1583, 1514 (C=N, C=C, N=H), 1330, 1151 (S=O), 1124 (C=S). ^**1**^ **H NMR (DMSO‐d**_**6**_ **/500 MHz) *δ* (ppm)**: 12.81 (1H, s, N_2_=H), 11.08 (1H, s, N_4_=H), 8.12 (1H, t, *J* = 1.9 Hz, H_4_‐ind.), 7.92 (1H, ddd, *J* = 8.1, 2.2, 1.1 Hz, H_2_‐ph.), 7.75 (1H, dt, *J* = 7.8, 1.4 Hz, H_6_‐ph.), 7.70–7.60 (2H, m, H_4,5_‐ph.), 7.48 (2H, s, SO_2_NH_2_), 7.27–7.15 (4H, m, H_6_‐ind., H_2,5,6_‐benz.), 7.10 (1H, d, *J* = 7.4 Hz, H_4_‐benz.), 6.93 (1H, d, *J* = 8.1 Hz, H_7_‐ind.), 4.93 (2H, s, N=CH_2_), 2.32 (3H, s, CH_3_), 2.28 (3H, s, CH_3_). ^**13**^ **C NMR (APT) (DMSO‐d**_**6**_ **/125 MHz) *δ* ppm**: 177.05 (C=S), 161.37 (C_2_‐ind.), 144.84 (C_3_‐ph.), 141.17 (C_7a_‐ind.), 139.38 (C_1_‐ph.), 138.44 (C_1_‐benz.), 136.10 (C_3_‐benz.), 132.73 (C_5_‐ind.), 132.30 (C_3_‐ind.), 132.22 (C_6_‐ind.), 129.46 (C_5,6_‐ph.), 129.07 (C_5_‐benz.), 128.77 (C_2_‐benz.), 128.37 (C_4_‐benz.), 124.99 (C_6_‐benz.), 123.60 (C_4_‐ph.), 123.07 (C_2_‐ph.), 122.14 (C_4_‐ind.), 119.83 (C_3a_‐ind.), 110.74 (C_7_‐ind.), 43.06 (NCH_2_), 21.45 (CH_3_), 21.06 (CH_3_). Anal. calcd. for C_24_H_23_N_5_O_3_S_2_·0.25H_2_O (511.60) C: 57.87, H: 4.76, N: 14.06. Found C: 57.55, H: 4.92, N: 14.29.

##### 4.1.1.2 1‐(4‐methylbenzyl)‐5‐methyl‐2‐oxoindolin‐3‐ylidene)‐4‐(3‐sulfamoylphenyl)thiosemicarbazide (6b)

Yellow powder (90%): m.p.: 232–237 °C. **IR (KBr) *ν***_**maks**_ **(cm** ^**−1**^ **)**: 3296, 3213 (N=H), 1689 (C=O), 1622, 1591, 1537 (C=N, C=C, N=H), 1348, 1161 (S=O), 1126 (C=S). ^**1**^ **H NMR (DMSO‐d**_**6**_ **/500 MHz) *δ* (ppm)**: 12.81 (1H, s, N_2_=H), 11.07 (1H, s, N_4_=H), 8.13 (1H, t, *J* = 2.0 Hz, H_4_‐ind.), 7.92 (1H, ddd, *J* = 8.0, 2.2, 1.1 Hz, H_2_‐ph), 7.77–7.72 (1H, m, H_6_‐ph.), 7.69–7.65 (1H, m, H_4_‐ph.), 7.64 (1H, t, *J* = 7.9 Hz, H_5_‐ph.), 7.48 (2H, s, SO_2_NH_2_), 7.31–7.25 (2H, m, H_2,6_‐benz.), 7.19 (1H, ddd, *J* = 8.1, 1.9, 0.9 Hz, H_6_‐ind.), 7.15 (2H, d, *J* = 7.9 Hz, H_3,5_‐benz.), 6.93 (1H, d, *J* = 8.1 Hz, H_7_‐ind.), 4.93 (2H, s, N=CH_2_), 2.32 (3H, s, CH_3_), 2.27 (3H, s, CH_3_). ^**13**^ **C NMR (APT) (DMSO‐d**_**6**_ **/125 MHz) *δ* ppm**: 177.05 (C=S), 161.35 (C_2_‐ind.), 144.84 (C_3_‐ph.), 141.12 (C_7a_‐ind.), 139.38 (C_1_‐ph.), 137.33 (C_1_‐benz.), 133.13 (C_4_‐benz.), 132.72 (C_5_‐ind.), 132.28 (C_3_‐ind.), 132.19 (C_6_‐ind.), 129.72 (C_3,5_‐benz.), 129.45 (C_5_‐ph.), 129.42 (C_6_‐ph.), 127.93 (C_2,6_‐benz.), 123.60 (C_4_‐ph.), 123.06 (C_2_‐ph.), 122.12 (C_4_‐ind.), 119.82 (C_3a_‐ind.), 110.78 (C_7_‐ind.), 42.84 (NCH_2_), 21.14 (CH_3_), 21.05 (CH_3_). Anal. calcd. for C_24_H_23_N_5_O_3_S_2_·0.25H_2_O (511.60) C: 57.87, H: 4.76, N: 14.06. Found C: 57.73, H: 4.85, N: 14.24.

##### 4.1.1.3 1‐(3‐fluorobenzyl)‐5‐methyl‐2‐oxoindolin‐3‐ylidene)‐4‐(3‐sulfamoylphenyl)thiosemicarbazide (6c)

Yellow powder (90%): m.p.: 230–235 °C. **IR (KBr) *ν***_**maks**_ **(cm** ^**−1**^ **)**: 3327, 3259 (N=H), 1693 (C=O), 1622, 1589, 1510, 1487 (C=N, C=C, N=H), 1330, 1153 (S=O), 1126 (C=S). ^**1**^ **H NMR (DMSO‐d**_**6**_ **/500 MHz) *δ* (ppm)**: 12.77 (1H, s, N_2_=H), 11.08 (1H, s, N_4_=H), 8.12 (1H, t, *J* = 2.0 Hz, H_4_‐ind.), 7.92 (1H, ddd, *J* = 8.1, 2.2, 1.1 Hz, H_2_‐ph.), 7.74 (1H, dt, *J* = 7.9, 1.3 Hz, H_6_‐ph.), 7.69 (1H, broad s, H_4_‐ph.), 7.64 (1H, t, *J* = 7.9 Hz, H_5_‐ph.), 7.48 (2H, s, SO_2_NH_2_), 7.40 (1H, td, *J* = 8.0, 6.1 Hz, H_5_‐benz.), 7.30–7.18 (3H, m, H_2,6_‐benz. and H_5_‐ind.), 7.17–7.09 (1H, m, H_4_‐benz.), 6.94 (1H, d, *J* = 8.1 Hz, H_7_‐ind.), 5.01 (2H, s, N=CH_2_), 2.32 (3H, s, CH_3_). ^**13**^ **C NMR (APT) (DMSO‐d**_**6**_ **/125 MHz) *δ* ppm**: 177.05 (C=S), 162.78 (d, *J* = 242.5 Hz, C_3_‐benz.), 161.44 (C_2_‐ind.), 144.84 (C_3_‐ph.), 141.00 (C_7a_‐ind.), 139.39 (C_1_‐ph.), 139.12 (d, *J* = 7.5 Hz, C_1_‐benz.), 132.80 (C_5_‐ind.), 132.34 (C_3_‐ind.), 132.20 (C_6_‐ind.), 131.19 (d, *J* = 8.7 Hz, C_5_‐benz.), 129.45 (C_5,6_‐ph.), 123.89 (d, *J* = 2.5 Hz, C_6_‐benz.), 123.60 (C_4_‐ph.), 123.07 (C_2_‐ph.), 122.18 (C_4_‐ind.), 119.94 (C_3a_‐ind.), 114.93 (d, *J* = 20.0 Hz, C_4_‐benz.), 114.76 (d, *J* = 22.5 Hz, C_2_‐benz.), 110.62 (C_7_‐ind.), 42.56 (NCH_2_), 21.06 (CH_3_). Anal. calcd. for C_23_H_20_FN_5_O_3_S_2_·0.25H_2_O (506.56) C: 55.02, H: 4.12, N: 13.95. Found C: 54.76, H: 4.08, N: 14.16.

##### 4.1.1.4 1‐(4‐fluorobenzyl)‐5‐methyl‐2‐oxoindolin‐3‐ylidene)‐4‐(3‐sulfamoylphenyl)thiosemicarbazide (6d)

Yellow powder (92%): m.p.: 235–240 °C. **IR (KBr) *ν***_**maks**_ **(cm** ^**−1**^ **)**: 3344, 3309, 3261 (N=H), 1681 (C=O), 1620, 1600, 1552, 1508 (C=N, C=C, N=H), 1336, 1155 (S=O), 1126 (C=S). ^**1**^ **H NMR (DMSO‐d**_**6**_ **/500 MHz) *δ* (ppm)**: 12.79 (1H, s, N_2_=H), 11.08 (1H, s, N_4_=H), 8.12 (1H, t, *J* = 2.0 Hz, H_4_‐ind.), 7.92 (1H, ddd, *J* = 8.0, 2.2, 1.1 Hz, H_2_‐ph.), 7.74 (1H, dt, *J* = 7.9, 1.3 Hz, H_6_‐ph.), 7.68 (1H, broad s, H_4_‐ph.), 7.64 (1H, t, *J* = 7.9 Hz, H_5_‐ph.), 7.50–7.42 (4H, m, SO_2_NH_2_, H_2,6_‐benz.), 7.24–7.14 (3H, m, H_5_‐ind., H_3,5_‐benz.), 6.97 (1H, d, *J* = 8.1 Hz, H_7_‐ind.), 4.97 (2H, s, N=CH_2_), 2.32 (3H, s, CH_3_). ^**13**^ **C NMR (APT) (DMSO‐d**_**6**_ **/125 MHz) *δ* ppm**: 177.05 (C=S), 162.05 (d, *J* = 242.5 Hz, C_4_‐benz.), 161.38 (C_2_‐ind.), 144.84 (C_3_‐ph.), 140.99 (C_7a_‐ind.), 139.38 (C_1_‐ph.), 132.79 (C_5_‐ind.), 132.43 (d, *J* = 2.5 Hz, C_1_‐benz.), 132.28 (C_3_‐ind.), 132.20 (C_6_‐ind.), 130.10 (d, *J* = 7.5 Hz, C_2,6_‐benz.), 129.45 (C_5_‐ph.), 129.42 (C_6_‐ph.), 123.60 (C_4_‐ph.), 123.06 (C_2_‐ph.), 122.17 (C_4_‐ind.), 119.88 (C_3a_‐ind.), 116.00 (d, *J* = 21.2 Hz, C_3,5_‐benz.), 110.68 (C_7_‐ind.), 42.36 (NCH_2_), 21.05 (CH_3_). Anal. calcd. for C_23_H_20_FN_5_O_3_S_2_ (497.56) C: 55.52, H: 4.05, N: 14.08. Found C: 55.14, H: 4.52, N: 14.01.

##### 4.1.1.5 1‐(3‐chlorobenzyl)‐5‐methyl‐2‐oxoindolin‐3‐ylidene)‐4‐(3‐sulfamoylphenyl)thiosemicarbazide (6e)

Yellow powder (88%): m.p.: 240–245 °C. **IR (KBr) *ν***_**maks**_ **(cm** ^**−1**^ **)**: 3332, 3259 (N=H), 1691 (C=O), 1624, 1597, 1514, 1487 (C=N, C=C, N=H), 1330, 1151 (S=O), 1126 (C=S). ^**1**^ **H NMR (DMSO‐d**_**6**_ **/500 MHz) *δ* (ppm)**: 12.76 (1H, s, N_2_=H), 11.07 (1H, s, N_4_=H), 8.12 (1H, t, *J* = 2.0 Hz, H_4_‐ind.), 7.92 (1H, ddd, *J* = 8.1, 2.2, 1.1 Hz, H_2_‐ph.), 7.75 (1H, dt, *J* = 7.9, 1.3 Hz, H_6_‐ph.), 7.69 (1H, d, *J* = 1.8 Hz, H_4_‐ph.), 7.64 (1H, t, *J* = 7.9 Hz, H_5_‐ph.), 7.50 (1H, d, *J* = 2.1 Hz, H_2_‐benz.), 7.48 (2H, s, SO_2_NH_2_), 7.42–7.33 (3H, m, C_3,4,5_=H‐benz.), 7.24–7.18 (1H, m, C_5_=H‐ind.), 6.95 (1H, d, *J* = 8.1 Hz, H_7_‐ind.), 5.00 (2H, s, N=CH_2_), 2.32 (3H, s, CH_3_). ^**13**^ **C NMR (APT) (DMSO‐d**_**6**_ **/125 MHz) *δ* ppm**: 177.04 (C=S), 161.45 (C_2_‐ind.), 144.84 (C_3_‐ph.), 140.97 (C_7a_‐ind.), 139.39 (C_1_‐ph.), 138.78 (C_1_‐benz.), 133.83 (C_3_‐benz.), 132.82 (C_5_‐ind.), 132.34 (C_3_‐ind.), 132.22 (C_6_‐ind.), 131.05 (C_5_‐benz.), 129.45 (C_5,6_‐ph.), 128.12 (C_2_‐benz.), 127.74 (C_4_‐benz.), 126.56 (C_6_‐benz.), 123.60 (C_4_‐ph.), 123.07 (C_2_‐ph.), 122.19 (C_4_‐ind.), 119.95 (C_3a_‐ind.), 110.60 (C_7_‐ind.), 42.50 (NCH_2_), 21.06 (CH_3_). Anal. calcd. for C_23_H_20_ClN_5_O_3_S_2_ (514.01) C: 53.74, H: 3.92, N: 13.63. Found C: 53.38, H: 4.22, N: 13.60.

##### 4.1.1.6 1‐(4‐chlorobenzyl)‐5‐methyl‐2‐oxoindolin‐3‐ylidene)‐4‐(3‐sulfamoylphenyl)thiosemicarbazide (6f)

Yellow powder (86%): m.p.: 220–224 °C. **IR (KBr) *ν***_**maks**_ **(cm** ^**−1**^ **)**: 3327, 3255 (N=H), 1693 (C=O), 1624, 1598, 1514, 1489 (C=N, C=C, N=H), 1332, 1155 (S=O), 1126 (C=S). ^**1**^ **H NMR (DMSO‐d**_**6**_ **/500 MHz) *δ* (ppm)**: 12.78 (1H, s, N_2_=H), 11.08 (1H, s, N_4_=H), 8.12 (1H, t, *J* = 2.0 Hz, H_4_‐ind.), 7.91 (1H, ddd, *J* = 8.1, 2.1, 1.1 Hz, H_2_‐ph.), 7.74 (1H, dt, *J* = 7.9, 1.3 Hz, H_6_‐ph.), 7.68 (1H, broad s, H_4_‐ph.), 7.64 (1H, t, *J* = 7.9 Hz, H_5_‐ph.), 7.48 (2H, s, SO_2_NH_2_), 7.46–7.38 (4H, m, H_2,3,5,6_‐benz.), 7.23–7.18 (1H, m, H_5_‐ind.), 6.94 (1H, d, *J* = 8.1 Hz, H_7_‐ind.), 4.98 (2H, s, N=CH_2_), 2.32 (3H, s, CH_3_). ^**13**^ **C NMR (APT) (DMSO‐d**_**6**_ **/125 MHz) *δ* ppm**: 177.04 (C=S), 161.40 (C_2_‐ind.), 144.84 (C_3_‐ph.), 140.95 (C_7a_‐ind.), 139.37 (C_1_‐ph.), 135.26 (C_1_‐benz.), 132.82 (C_5_‐ind.), 132.73 (C_4_‐benz.), 132.25 (C_3_‐ind.), 132.21 (C_6_‐ind.), 129.86 (C_3,5_‐benz.), 129.46 (C_5_‐ph.), 129.43 (C_6_‐ph.), 129.13 (C_2,6_‐benz.), 123.60 (C_4_‐ph.), 123.06 (C_2_‐ph.), 122.18 (C_4_‐ind.), 119.89 (C_3a_‐ind.), 110.66 (C_7_‐ind.), 42.40 (NCH_2_), 21.05 (CH_3_). Anal. calcd. for C_23_H_20_ClN_5_O_3_S_2_ (514.01) C: 53.74, H: 3.92, N: 13.63. Found C: 53.39, H: 4.03, N: 13.70.

##### 4.1.1.7 1‐(3‐methylbenzyl)‐5‐chloro‐2‐oxoindolin‐3‐ylidene)‐4‐(3‐sulfamoylphenyl)thiosemicarbazide (6g)

Orange powder (92%): m.p.: 228–232 °C. **IR (KBr) *ν***_**maks**_ **(cm** ^**−1**^ **)**: 3329, 3304, 3253 (N=H), 1687 (C=O), 1612, 1598, 1537 (C=N, C=C, N=H), 1332, 1153 (S=O), 1120 (C=S). ^**1**^ **H NMR (DMSO‐d**_**6**_ **/500 MHz) *δ* (ppm)**: 12.64 (1H, s, N_2_=H), 11.14 (1H, s, N_4_=H), 8.12 (1H, t, *J* = 2.0 Hz, H_2_‐ph.), 7.95 (1H, d, *J* = 2.2 Hz, H_4_‐ind.), 7.91 (1H, ddd, *J* = 8.0, 2.2, 1.1 Hz, H_6_‐ph.), 7.76 (1H, dt, *J* = 8.0, 1.3 Hz, H_4_‐ph.), 7.66 (1H, t, *J* = 7.9 Hz, H_5_‐ph.), 7.49 (2H, s, SO_2_NH_2_), 7.44 (1H, dd, *J* = 8.5, 2.3 Hz, H_6_‐ind.), 7.28–7.16 (3H, m, H_2,5,6_‐benz.), 7.11 (1H, d, *J* = 7.3 Hz, H_4_‐benz.), 7.05 (1H, d, *J* = 8.5 Hz, H_7_‐ind.), 4.96 (2H, s, N=CH_2_), 2.28 (3H, s, CH_3_). ^**13**^ **C NMR (APT) (DMSO‐d**_**6**_ **/125 MHz) *δ* ppm**: 177.02 (C=S), 161.12 (C_2_‐ind.), 144.92 (C_3_‐ph.), 141.95 (C_7a_‐ind.), 139.20 (C_1_‐ph.), 138.48 (C_1_‐benz.), 135.77 (C_3_‐benz.), 131.04 (C_6_‐ind.), 130.94 (C_3_‐ind.), 129.56 (C_5_‐ph.), 129.45 (C_6_‐ph.), 129.09 (C_5_‐benz.), 128.84 (C_2_‐benz.), 128.38 (C_4_‐benz.), 127.86 (C_3a_‐ind.), 124.99 (C_6_‐benz.), 123.76 (C_4_‐ph.), 123.07 (C_2_‐ph.), 121.76 (C_5_‐ind.), 121.36 (C_4_‐ind.), 112.45 (C_7_‐ind.), 43.15 (NCH_2_), 21.44 (CH_3_). Anal. calcd. for C_23_H_20_ClN_5_O_3_S_2_·0.25H_2_O (523.01) C: 53.28, H: 3.99, N: 13.51. Found C: 3.96, H: 13.44, N: 12.86.

##### 4.1.1.8 1‐(4‐methylbenzyl)‐5‐chloro‐2‐oxoindolin‐3‐ylidene)‐4‐(3‐sulfamoylphenyl)thiosemicarbazide (6h)

Orange powder (88%): m.p.: 206–212 °C. **IR (KBr) *ν***_**maks**_ **(cm** ^**−1**^ **)**: 3313, 3234 (N=H), 1691 (C=O), 1612, 1593, 1548 (C=N, C=C, N=H), 1334, 1155 (S=O), 1143 (C=S). ^**1**^ **H NMR (DMSO‐d**_**6**_ **/500 MHz) *δ* (ppm)**: 12.65 (1H, s, N_2_=H), 11.14 (1H, s, N_4_=H), 8.11 (1H, t, *J* = 2.0 Hz, H_4_‐ind.), 7.96–7.88 (2H, m, H_2,6_‐ph.), 7.75 (1H, dt, *J* = 7.9, 1.4 Hz, H_4_‐ph.), 7.65 (1H, t, *J* = 8.0 Hz, H_5_‐ph.), 7.49 (2H, s, SO_2_NH_2_), 7.44 (1H, dd, *J* = 8.4, 2.3 Hz, H_6_‐ind.), 7.29 (2H, d, *J* = 7.8 Hz, H_2,6_‐benz.), 7.16 (2H, d, *J* = 7.8 Hz, H_3,5_‐benz.), 7.05 (1H, d, *J* = 8.5 Hz, H_7_‐ind.), 4.96 (2H, s, N=CH_2_), 2.27 (3H, s, CH_3_). ^**13**^ **C NMR (APT) (DMSO‐d**_**6**_ **/125 MHz) *δ* ppm**: 177.02 (C=S), 161.11 (C_2_‐ind.), 144.91 (C_3_‐ph.), 141.89 (C_7a_‐ind.), 139.20 (C_1_‐ph.), 137.42 (C_1_‐benz.), 132.81 (C_4_‐benz.), 131.01 (C_6_‐ind.), 130.93 (C_3_‐ind.), 129.75 (C_3,5_‐benz.), 129.55 (C_5_‐ph.), 129.44 (C_6_‐ph.), 127.94 (C_2,6_‐benz.), 127.84 (C_5_‐ind.), 123.75 (C_4_‐ph.), 123.06 (C_2_‐ph.), 121.75 (C_3a_‐ind.), 121.35 (C_4_‐ind.), 112.50 (C_7_‐ind.), 42.93 (NCH_2_), 21.14 (CH_3_). Anal. calcd. for C_23_H_20_ClN_5_O_3_S_2_·H_2_O (532.01) C: 51.92, H: 4.17, N: 13.16. Found C: 51.56, H: 4.17, N: 13.08.

##### 4.1.1.9 1‐(3‐fluorobenzyl)‐5‐chloro‐2‐oxoindolin‐3‐ylidene)‐4‐(3‐sulfamoylphenyl)thiosemicarbazide (6i)

Yellow powder (91%): m.p.: 202–204 °C. **IR (KBr) *ν***_**maks**_ **(cm** ^**−1**^ **)**: 3334, 3224 (N=H), 1689 (C=O), 1614, 1593, 1483, 1465, 1445 (C=N, C=C, N=H), 1336, 1159 (S=O), 1124 (C=S). ^**1**^ **H NMR (DMSO‐d**_**6**_ **/400 MHz) *δ* (ppm)**: 12.61 (1H, s, N_2_=H), 11.13 (1H, s, N_4_=H), 8.10 (1H, t, *J* = 2.0 Hz, H_2_‐ph.), 7.94 (1H, d, *J* = 2.2 Hz, H_4_‐ind.), 7.89 (1H, d, *J* = 2.2 Hz, H_6_‐ph.), 7.75 (1H, dt, *J* = 7.9, 1.3 Hz, H_4_‐ph.), 7.65 (1H, dt, *J* = 7.9, 1.3 Hz, H_5_‐ph.), 7.48 (2H, s, SO_2_NH_2_), 7.46 (1H, d, *J* = 7.8 Hz, H_6_‐ind.), 7.45–7.10 (4H, m, benz‐H), 7.05 (1H, d, *J =* 8.4 Hz, H_7_‐ind.), 5.03 (2H, s, N=CH_2_‐ind.). ^**13**^ **C NMR (APT) (DMSO‐d**_**6**_ **/125 MHz) *δ* ppm**: 177.02 (C=S), 162.80 (d, *J* = 242.5 Hz, C_3_‐benz.), 161.20 (C_2_‐ind.), 144.91 (C_3_‐ph.), 141.80 (C_7a_‐ind.), 139.20 (C_1_‐ph.), 138.80 (d, *J* = 7.5 Hz, C_1_‐benz.), 131.20 (d, *J* = 8.7 Hz, C_5_‐benz.), 131.02 (C_6_‐ind.), 131.02 (C_3_‐ind.), 129.55 (C_5_‐ph.), 129.46 (C_6_‐ph.), 127.91 (C_5_‐ind.), 123.92 (d, *J* = 1.2 Hz, C_6_‐benz.), 123.76 (C_4_‐ph.), 123.07 (C_2_‐ph.), 121.89 (C_3a_‐ind.), 121.39 (C_4_‐ind.), 114.99 (d, *J* = 20.8 Hz, C_4_‐benz.), 114.77 (d, *J* = 21.25 Hz, C_2_‐benz.), 112.35 (C_7_‐ind.), 42.64 (NCH_2_). Anal. calcd. for C_22_H_17_ClFN_5_O_3_S_2_·H_2_O (536.00) C: 49.29, H: 3.94, N: 13.06. Found C: 49.51, H: 3.61, N: 13.13.

##### 4.1.1.10 1‐(4‐fluorobenzyl)‐5‐chloro‐2‐oxoindolin‐3‐ylidene)‐4‐(3‐sulfamoylphenyl)thiosemicarbazide (6j)

Orange powder (79%): m.p.: 217–224 °C. **IR (KBr) *ν***_**maks**_ **(cm** ^**−1**^ **)**: 3398, 3307, 3246 (N=H), 1695 (C=O), 1612, 1593, 1548, 1508, 1465 (C=N, C=C, N=H), 1340, 1151 (S=O), 1151 (C=S). ^**1**^ **H NMR (DMSO‐d**_**6**_ **/400 MHz) *δ* (ppm)**: 12.61 (1H, s, N_2_=H), 11.13 (1H, s, N_4_=H), 8.10 (1H, t, *J* = 2.0 Hz, H_2_‐ph.), 7.94 (1H, d, *J = *2.2 Hz, H_4_‐ind.), 7.89 (1H, d, *J* = 8.0 Hz, H_6_‐ph.), 7.74 (1H, d, *J* = 7.8 Hz, H_4_‐ph.), 7.64 (1H, t, *J* = 7.9 Hz, H_5_‐ph.), 7.47 (2H, s, SO_2_NH_2_), 7.43–7.37 (3H, m, H_6_‐ind., H_3,5_‐benz.), 7.18 (2H, (t, *J* = 8.8 Hz, H_2,6_‐benz.), 7.09 (1H, d, *J* = 8.5 Hz, H_7_‐ind.), 4.99 (2H, s, N=CH_2_). ^**13**^ **C NMR (APT) (DMSO‐d**_**6**_ **/125 MHz) *δ* ppm**: 177.01 (C=S), 162.06 (d, *J* = 242.5 Hz, C_4_‐benz.), 161.14 (C_2_‐ind.), 144.91 (C_3_‐ph.), 141.78 (C_7a_‐ind.), 139.19 (C_1_‐ph.), 132.11 (d, *J* = 3.7 Hz, C_1_‐benz.), 131.01 (C_6_‐ind.), 130.94 (C_3_‐ind.), 130.12 (d, *J* = 8.7 Hz, C_2_,_6_‐benz.), 129.55 (C_5_‐ph.), 129.45 (C_6_‐ph.), 127.90 (C_5_‐ind.), 123.76 (C_4_‐ph.), 123.07 (C_2_‐ph.), 121.82 (C_3a_‐ind.), 121.39 (C_4_‐ind.), 115.97 (d, *J* = 21.2 Hz, C_3_,_5_‐benz.), 112.40 (C_7_‐ind.), 42.46 (NCH_2_). Anal. calcd. for C_22_H_17_ClFN_5_O_3_S_2_ (517.98) C: 51.01, H: 3.31, N: 13.52. Found C: 50.84, H: 3.45, N: 13.51.

##### 4.1.1.11 1‐(3‐chlorobenzyl)‐5‐chloro‐2‐oxoindolin‐3‐ylidene)‐4‐(3‐sulfamoylphenyl)thiosemicarbazide (6k)

Yellow powder (81%): m.p.: 190–195 °C. **IR (KBr) *ν***_**maks**_ **(cm** ^**−1**^ **)**: 3397, 3335, 3258 (N=H), 1691 (C=O), 1612, 1598, 1579, 1516, 1467 (C=N, C=C, N=H), 1336, 1159 (S=O), 1150 (C=S). ^**1**^ **H NMR (DMSO‐d**_**6**_ **/400 MHz) *δ* (ppm)**: 12.58 (1H, s, N_2_=H), 11.11 (1H, s, N_4_=H), 8.08 (1H, t, *J* = 2.0 Hz, H_2_‐ph.), 7.93 (1H, d, *J* = 2.2, H_4_‐ind.), 7.89 (1H, d, *J* = 8.0, H_6_‐ph.), 7.72 (1H, dt, *J* = 8.0, 1.3 Hz, H_4_‐ph.), 7.62 (1H, t, *J* = 7.9 Hz, H_5_‐ph.), 7.49 (1H, s, H_2_‐benz.), 7.44 (2H, s, SO_2_NH_2_), 7.42 (1H, d, *J* = 8.4 Hz, H_6_‐ind.), 7.35 (3H, s, H_4_, H_5_, H_6_‐benz.), 7.04 (1H, d, *J* = 8.5 Hz, H_7_‐ind.), 5.00 (2H, s, N=CH_2_). ^**13**^ **C NMR (APT) (DMSO‐d**_**6**_ **/125 MHz) *δ* ppm**: 177.01 (C=S), 161.21 (C_2_‐ind.), 144.92 (C_3_‐ph.), 141.78 (C_7a_‐ind), 139.20 (C_1_‐ph.), 138.47 (C_1_‐benz.), 133.87 (C_3_‐benz._,_ C_3_‐ind.), 131.05 (C_5_‐benz., C_6_‐ind.), 129.55 (C_5_‐ph.), 129.45 (C_6_‐ph.), 128.19 (C_2_‐benz.), 127.93 (C_5_‐ind.), 127.76 (C_4_‐benz.), 126.58 (C_6_‐benz.), 123.76 (C_4_‐ph.), 123.07 (C_2_‐ph.), 121.90 (C_3a_‐ind.), 121.41 (C_4_‐ind.), 112.33 (C_7_‐ind.), 42.60 (NCH_2_). Anal. calcd. for C_22_H_17_Cl_2_N_5_O_3_S_2_ (534.44) C: 49.44, H: 3.21, N: 13.10. Found C: 48.83, H: 3.29, N: 13.16.

##### 4.1.1.12 1‐(4‐chlorobenzyl)‐5‐chloro‐2‐oxoindolin‐3‐ylidene)‐4‐(3‐sulfamoylphenyl)thiosemicarbazide (6l)

Orange powder (90%): m.p.: 240–243 °C. **IR (KBr) *ν***_**maks**_ **(cm** ^**−1**^ **)**: 3335, 3273 (N=H), 1678 (C=O), 1612, 1593, 1541, 1485 (C=N, C=C, N=H), 1333, 1163 (S=O), 1149 (C=S). ^**1**^ **H NMR (DMSO‐d**_**6**_ **/400 MHz) *δ* (ppm)**: 12.63 (1H, s, N_2_=H), 11.08 (1H, s, N_4_=H), 7.95 (1H, t, *J* = 2.0 Hz, H_4_‐ind.), 7.88 (4H, s, N_4_=C_6_H_4_), 7.45 (1H, dt, *J* = 7.8, 1.4 Hz, H_6_‐ind.), 7.44–7.41 (4H, m, benz. C=H), 7.40 (2H, s, SO_2_NH_2_), 7.06 (1H, d, *J* = 8.5 Hz, H_7_‐ind.), 5.00 (2H, s, N=CH_2_). ^**13**^ **C NMR (APT) (DMSO‐d**_**6**_ **/125 MHz) *δ* ppm**: 177.01 (C=S), 161.15 (C_2_‐ind.), 144.92 (C_3_‐ph.), 141.74 (C_7a_‐ind.), 139.19 (C_1_‐ph.), 134.94 (C_1_‐benz.), 132.82 (C_4_‐benz.), 131.02 (C_6_‐ind.), 130.91 (C_3_‐ind.), 129.88 (C_3,5_‐benz.), 129.55 (C_5_‐ph.), 129.44 (C_6_‐ph.), 129.14 (C_2,6_‐benz.), 127.94 (C_5_‐ind.), 123.77 (C_4_‐ph.), 123.07 (C_2_‐ph.), 121.83 (C_3a_‐ind.), 121.40 (C_4_‐ind.), 112.38 (C_7_‐ind.), 42.51 (NCH_2_). Anal. calcd. for C_22_H_17_Cl_2_N_5_O_3_S_2_ (534.43) C: 49.44, H: 3.21, N: 13.10. Found C: 48.86, H: 3.26, N: 13.52.

### 4.2 CA Inhibitory Activity

#### 4.2.1 Purification of hCA I and II Isoenzymes

Human blood was collected from the blood center at Ataturk University, stored at 4 °C and used within 2–3 days. Erythrocytes were separated by centrifugation at 3000 × g for 20 min, and the plasma and buffy coat were carefully removed prior to purifying hCA I and II via affinity chromatography. The remaining erythrocytes were washed twice with 0.9% NaCl solution, discarding the supernatant each time. Hemolysis was then carried out by adding distilled water at 0 °C, and the mixture was stirred for 30 min at 4 °C. The hemolysate was centrifuged at 15,000 × g for 30 min to separate the cell membranes, and the pH was adjusted to 8.7 using solid Tris before applying it to the column [31]. The affinity gel was prepared on a Sepharose‐4B matrix, which was first activated with CNBr, followed by covalent attachment of L‐tyrosine. Sulfanilamide was subsequently coupled to tyrosine via a diazotization reaction to serve as the ligand. The hemolysate was then applied to a Sepharose‐4B–L‐tyrosine–sulfanilamide affinity column equilibrated with 25 mM Tris‐HCl/0.1 M Na_2_SO_4_ (pH 8.7), and the column was washed with 25 mM Tris‐HCl/22 mM Na_2_SO_4_ (pH 8.7). hCA I and II were eluted using 1 M NaCl/25 mM Na_2_HPO_4_ (pH 6.3) and 0.1 M CH_3_COONa/0.5 M NaClO_4_ (pH 5.6), respectively. All procedures were performed at 4 °C [32]. hCA IX and XII were commercially obtained and dissolved immediately before use.

#### 4.2.2 Determination of CA Inhibitory Activity

Activity measurements were performed based on the hydratase activity of the enzyme. Briefly, the hydratase activity of hCA was determined by monitoring the enzyme‐catalyzed hydration of CO_2_ in 10 mM HEPES buffer (pH 7.4) containing 0.1 M Na_2_SO_4_, using 0.2 mM phenol red as a pH indicator. The inhibitory effects of the compounds **6a–l** on the hydratase activities of hCA I, II, IX, and XII isoenzymes were evaluated. Stock solutions of all compounds were prepared at a concentration of 10 mM in a DMSO–distilled water mixture, and five or seven different inhibitor concentrations were used to determine inhibition constants. Compound samples and enzymes were incubated at room temperature for 10 min to allow formation of the enzyme–inhibitor (*E*–*I*) complex, if applicable. Subsequently, the substrate and indicator were added to the reaction medium, and measurements were performed. The inhibition of hCA I, II, IX, and XII hydratase activity by compounds **6a–l** was assessed. Triplicate measurements were carried out for each inhibitor concentration, and the results were expressed as mean values. IC_50_ values for each compound exhibiting inhibition were calculated using % activity–inhibitor concentration plots. *K* *_i_* were calculated using the Cheng–Prusoff equation by applying nonlinear least‐squares methods [29, 33, 34].

### 4.3 Docking Studies

#### 4.3.1 Molecular Modeling Studies

Molecular modeling studies of the compounds were performed using the Schrödinger Suite 2025‐2, and Maestro was employed for visualization of the obtained results [35].

#### 4.3.2 Preparation of Protein Structures

The crystal structures of hCA IX and hCA XII were processed using the Protein Preparation Wizard. In cases where the enzymes consisted of multiple subunits, only chain A was retained together with the catalytic zinc ion and the cocrystallized ligand in the active site, while the other subunits were removed prior to subsequent analyses [36]. Missing hydrogen atoms and incomplete residue side chains were added and refined using Prime [37]. Water molecules located more than 5 Å away from the cocrystallized ligand were removed. The protonation states and tautomeric forms of ionizable residues were assigned at physiological pH (7.4) using PROPKA. Furthermore, the N‐ and C‐termini of the protein chains were capped. Finally, the prepared structures were subjected to energy minimization employing the OPLS4 force field. For the current study, crystal structures of hCA IX PDB ID: 8Q18 and hCA XII PDB ID: 4KP5 were used [38, 39].

#### 4.3.3 Grid Generation

Receptor grids of proteins were generated using the Receptor Grid Generation module, with the center of the grid defined by the centroid of the cognate ligand. The binding site was defined by a grid box with a 15 Å radius surrounding the cocrystallized ligand [40].

#### 4.3.4 Ligand Preparation

All synthesized compounds, along with the cocrystallized ligands, were processed using the LigPrep module with the OPLS4 force field. Possible tautomeric states and stereoisomers were generated using Epik at physiological pH (7.4) [41, 42].

#### 4.3.5 Docking Studies

Molecular docking studies were initiated by retrieving the target enzymes from the Protein Data Bank (PDB), namely PDB ID: 8Q18 for hCA IX and PDB ID: 4KP5 for hCA XII. The hydroxyl and thiol groups of Thr, Ser, Cys, and Tyr residues located 5.5 Å from the ligand (those not involved in hydrogen bonding networks or disulfide bond formation) were allowed to rotate to facilitate potential interactions with the docked ligands. All ligands were docked using standard precision mode with flexible ligand sampling using Glide module [40]. For each ligand, 10 poses were generated, and the highest‐scoring conformations were visually evaluated and subsequently refined through postdocking energy minimization. Other docking parameters were maintained at their default. To validate the docking protocol, cognate ligands were redocked and RMSD values were found to be below 2 Å, indicating the reliability of the docking procedure.

#### 4.3.6 MD Simulations

Binding modes of the inhibitors were further examined by MD simulations carried out using Desmond [43]. The selected protein–ligand complexes were placed at the center of an orthorhombic simulation box with periodic boundary conditions, maintaining a minimum distance of 20 Å from the box edges. The systems were solvated using the TIP3P water model, and appropriate counter ions (NaCl) were introduced to ensure overall charge neutrality. All simulations were conducted employing the OPLS4 force field. MD simulations were performed for 100 ns under NPT ensemble conditions. Also, temperature (300 K) and pressure (1.01325 bar) were controlled using the Nose–Hoover thermostat and Martyna–Tobias–Klein barostat. Trajectories were saved at intervals of 10 ps and analyzed using the Simulation Interaction Diagram tool available in the Schrödinger suite.

#### 4.3.7 Calculation of Pharmacokinetic Parameters

Compounds **6a–l** were examined in terms of RO5 rules and in silico drug‐ability criteria on Qikprop application [30, 44].

## Conflicts of Interest

The authors declare no conflicts of interest.

## Supporting information

Supplementary Material

## Data Availability

The data that supports the findings of this study are available in the Supporting Information of this article.
