# Supplementary material for: Synthesis, Moleculer Modeling, and Carbonic Anhydrase Inhibitor Effects of Novel 1‐(1‐benzyl‐2‐oxoindolin‐3‐ylidene)‐4‐(3‐sulfamoylphenyl)thiosemicarbazides
Source: ChemMedChem. 2026 Oct 12;21(19):e70467. doi: 10.1002/cmdc.70467 (PMC13655631; doi:10.1002/cmdc.70467)
Supplement: Supplementary file 1 [file CMDC-21-e70467-s001.pdf]

Synthesis, moleculer modeling and carbonic anhydrase inhibitor effects of novel 1-(1-benzyl-2-oxoindolin-3-ylidene)-4-(3-sulfamoylphenyl)thiosemicarbazides

Çağla Begüm Apaydın[1], Tuğçe Cinek[2], Hayrunnisa Nadaroğlu[3], Nilgün Karalı[1]

[1] Department of Pharmaceutical Chemistry, Faculty of Pharmacy, Istanbul University, Fatih, 34452, Istanbul, Turkey

[2] Department of Pharmaceutical Chemistry, Faculty of Pharmacy, Istanbul Health and Technology University, Istanbul, 34275, Turkey

[3] Department of Food Technology, Vocational College of Technical Sciences, Atatürk University, Erzurum, Türkiye

(Supplementary Material)

1-(3-Methylbenzyl-5-methyl-2-oxoindolin-3-ylidene)-4-(3-sulfamoylphenyl)thiosemicarbazide (6a)

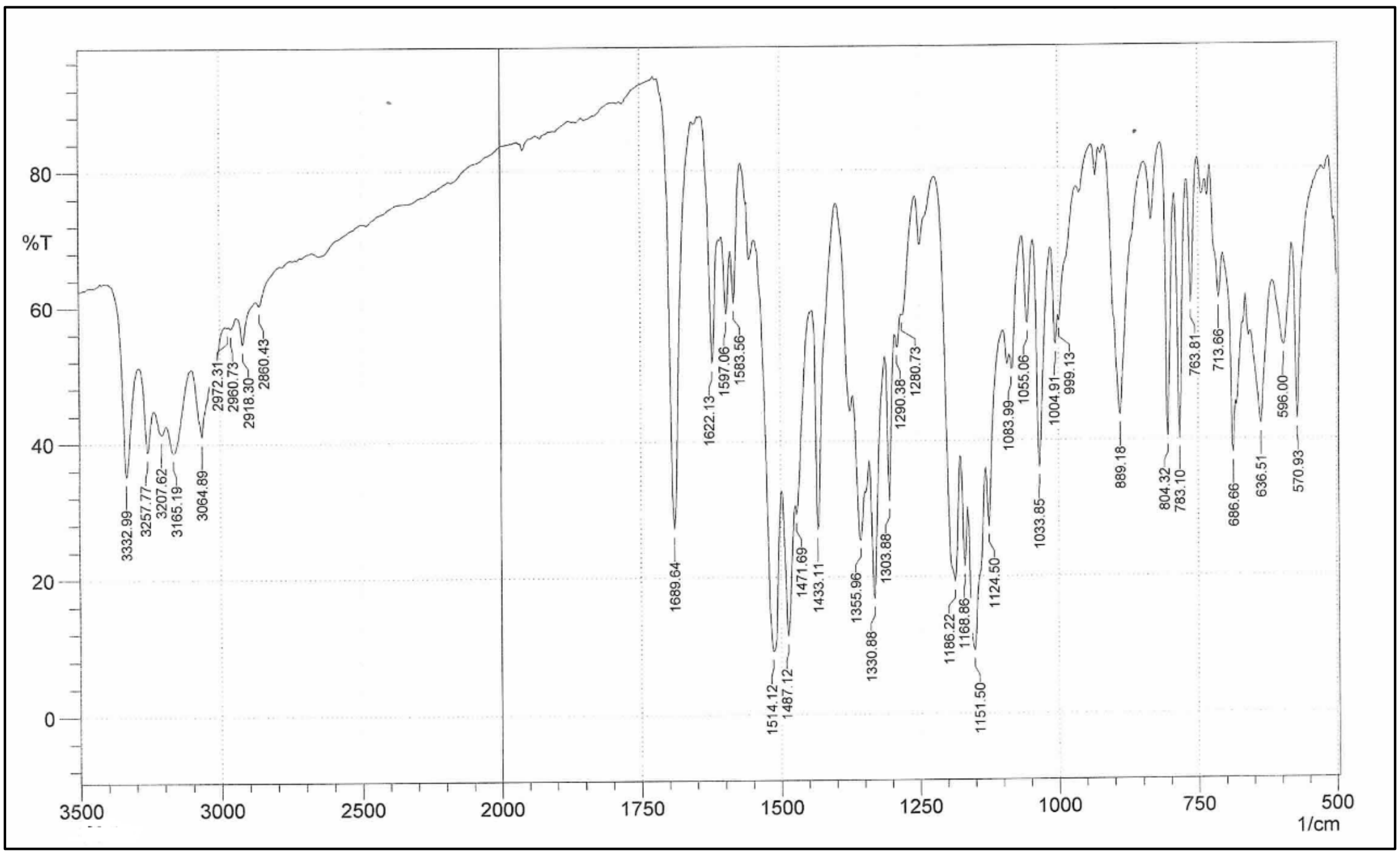

Figure S1: IR spectrum of compound **6a**

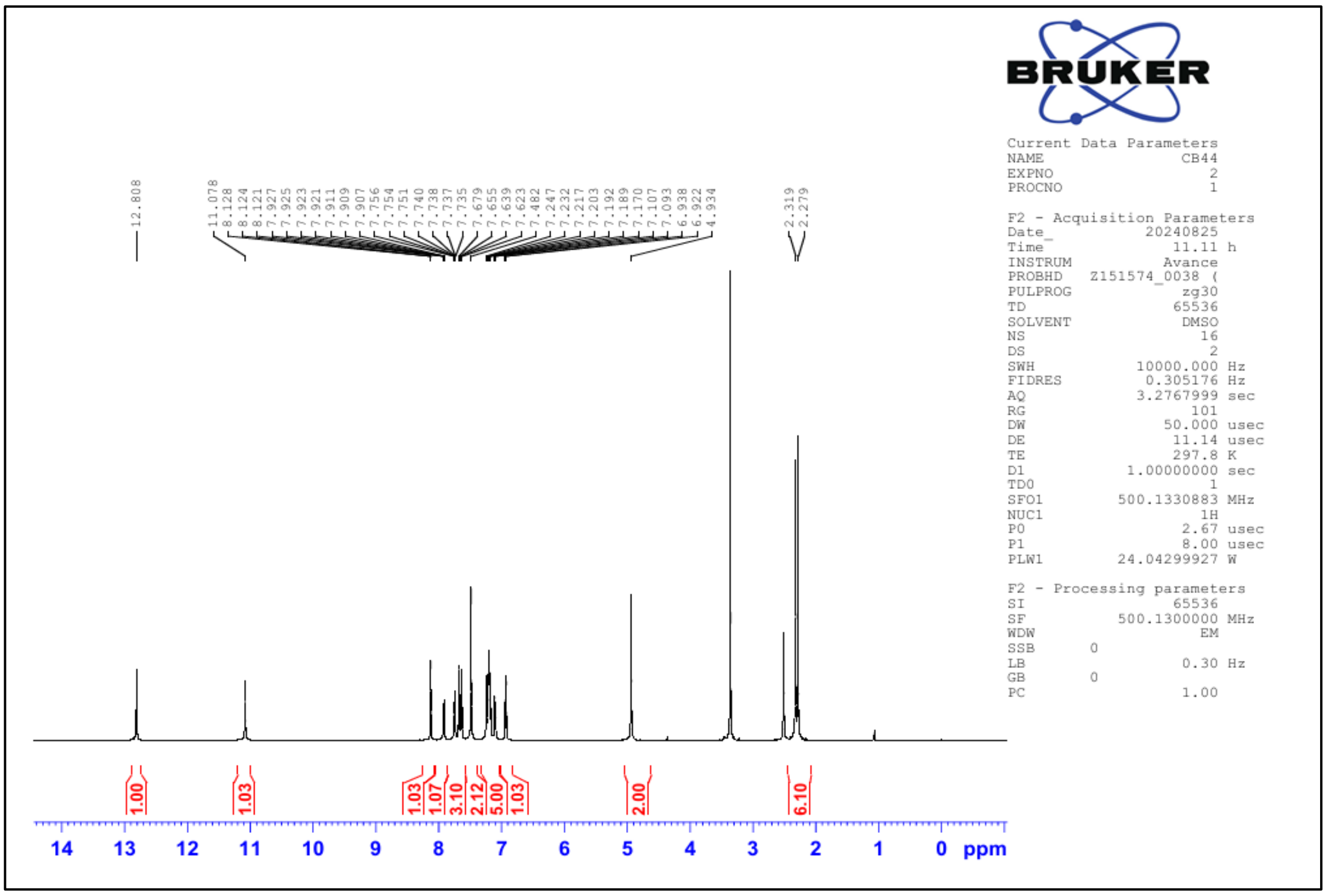

Figure S2: ^{1}H NMR of compound **6a**

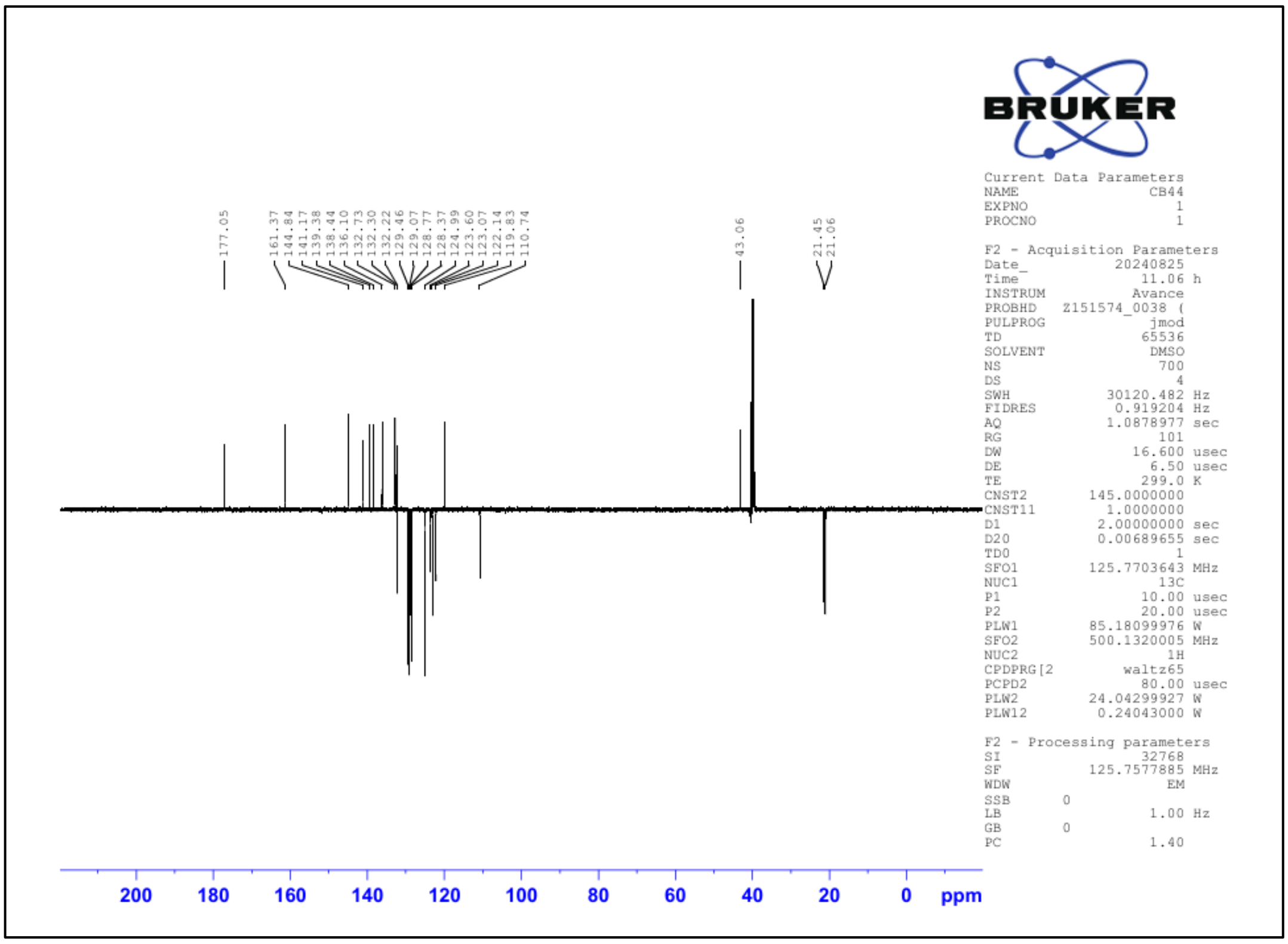

Figure S3: ^{13}C NMR of compound **6a** (APT)

1-(4-Methylbenzyl)-5-methyl-2-oxoindolin-3-ylidene)-4-(3-sulfamoylphenyl)thiosemicarbazide (6b)

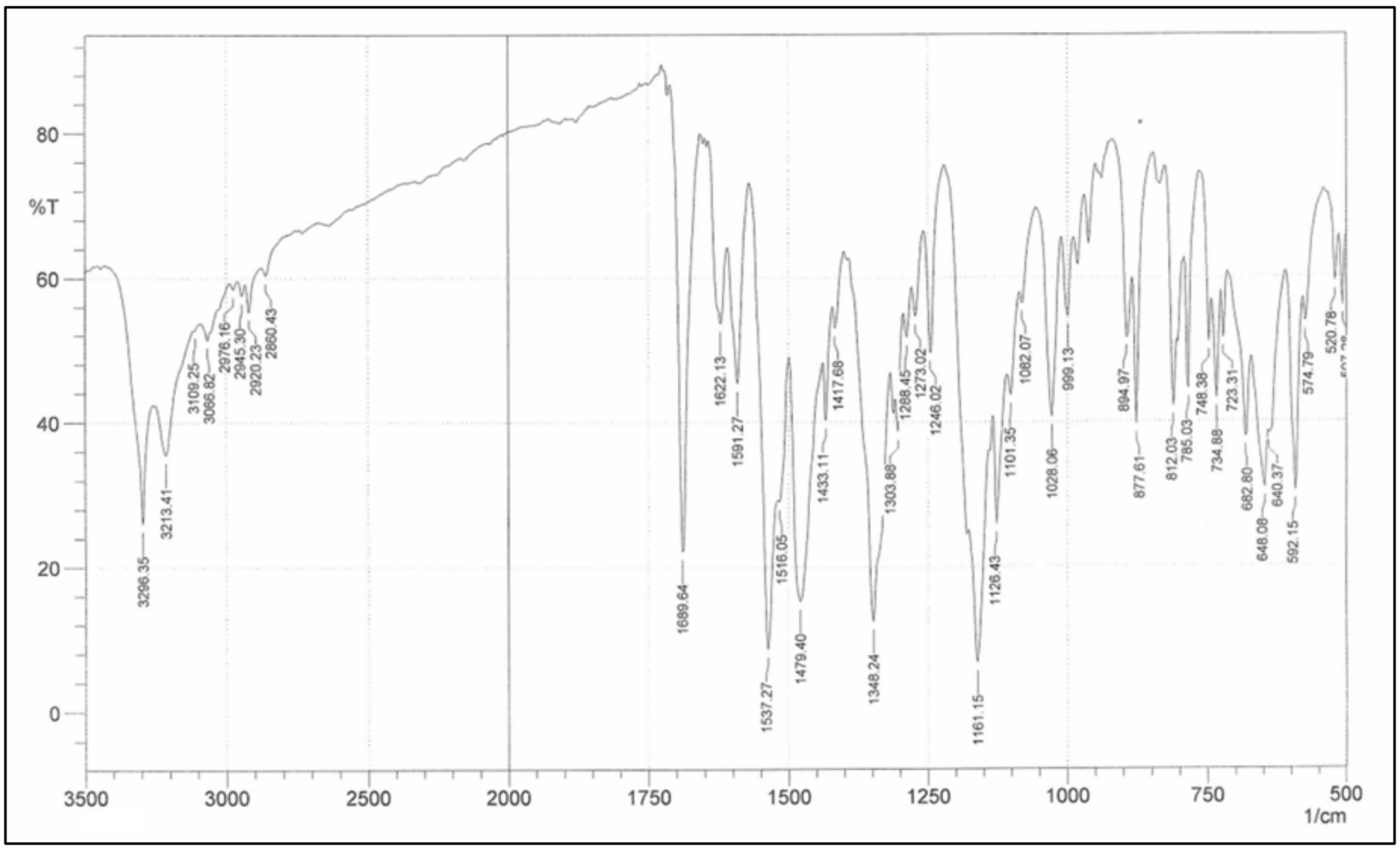

Figure S4: IR spectrum of compound **6b**

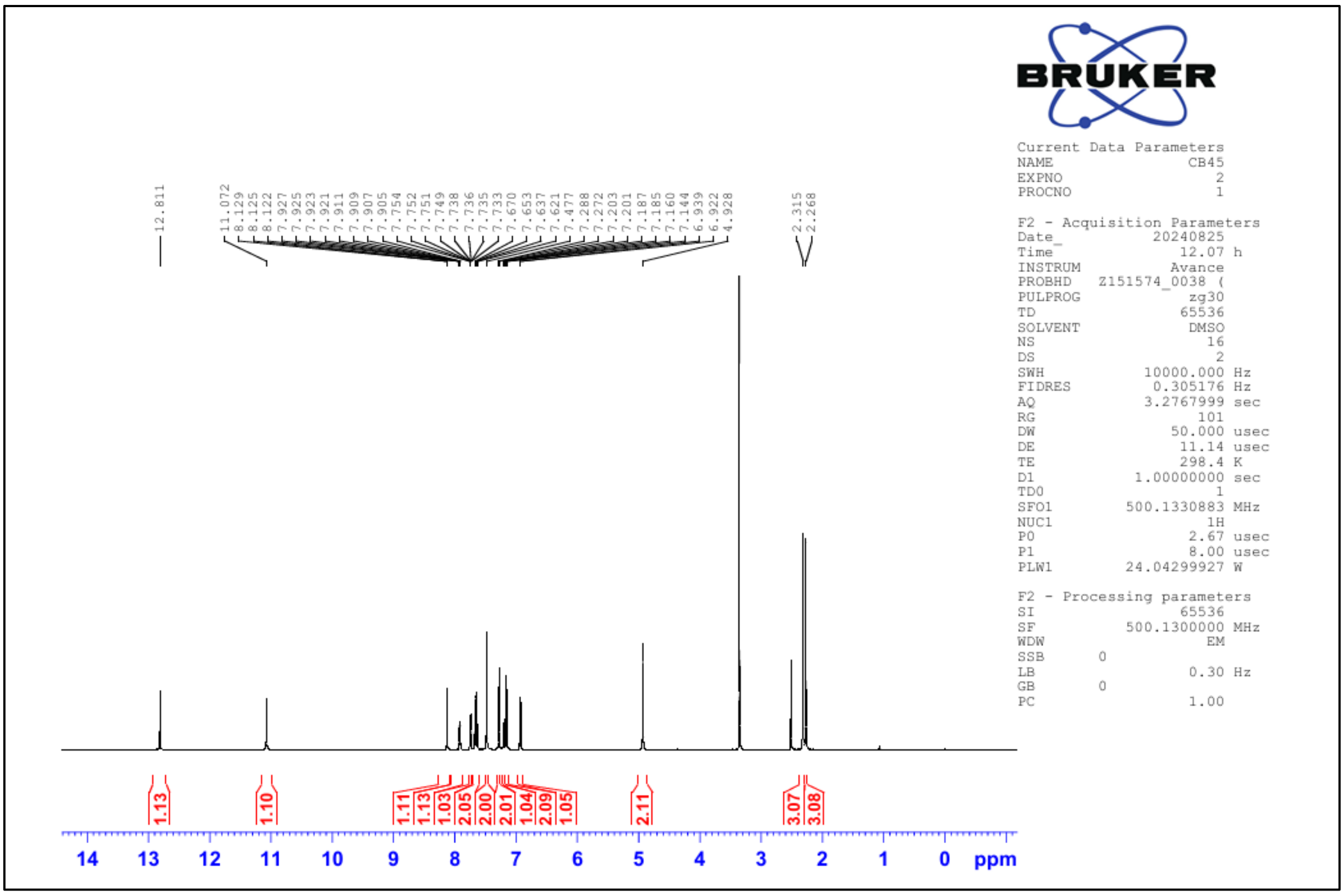

Figure S5: ^{1}H NMR of compound **6b**

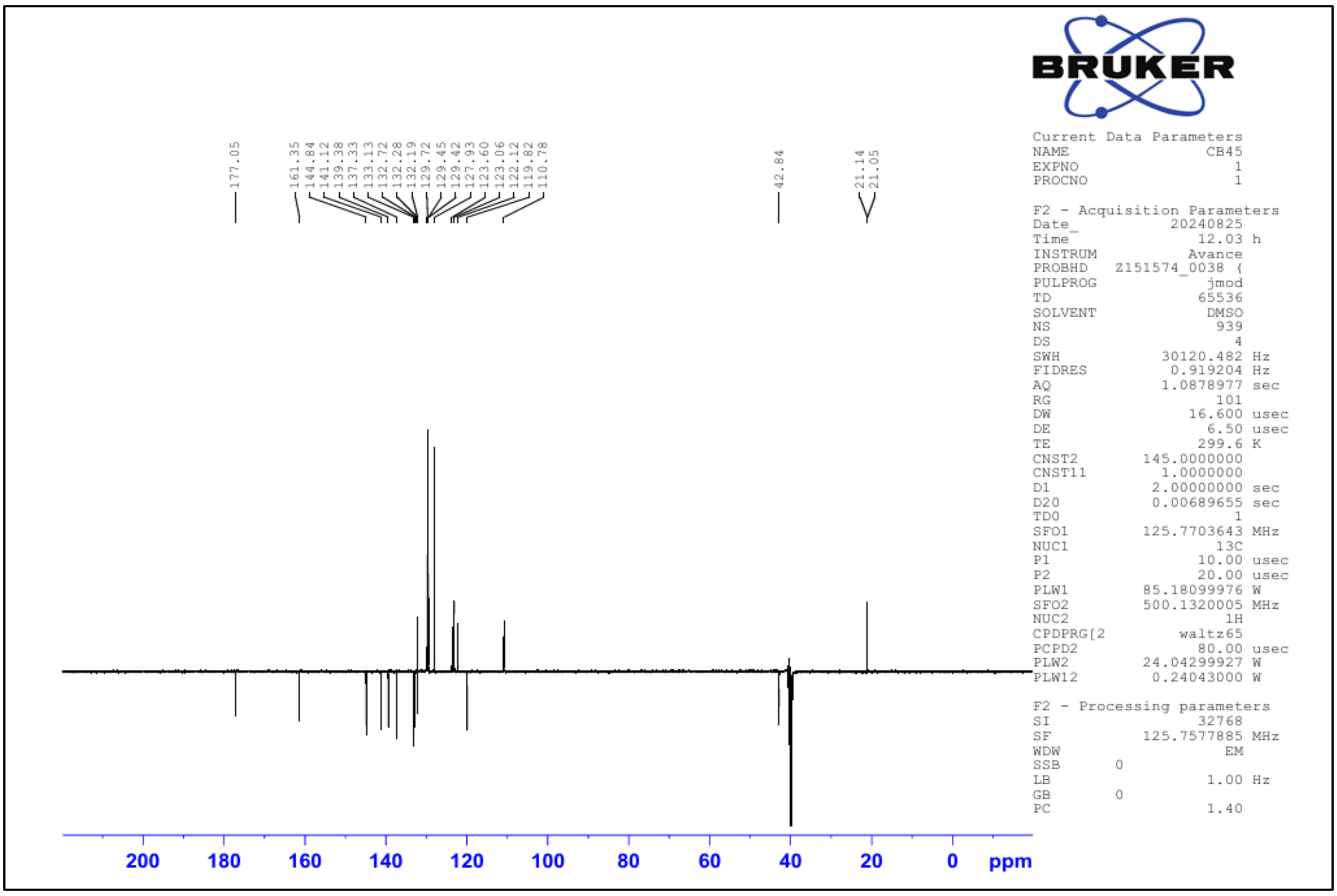

Figure S6: ^{13}C NMR of compound **6b** (APT)

1-(3-Fluorobenzyl)-5-methyl-2-oxoindolin-3-ylidene)-4-(3-sulfamoylphenyl)thiosemicarbazide (6c)

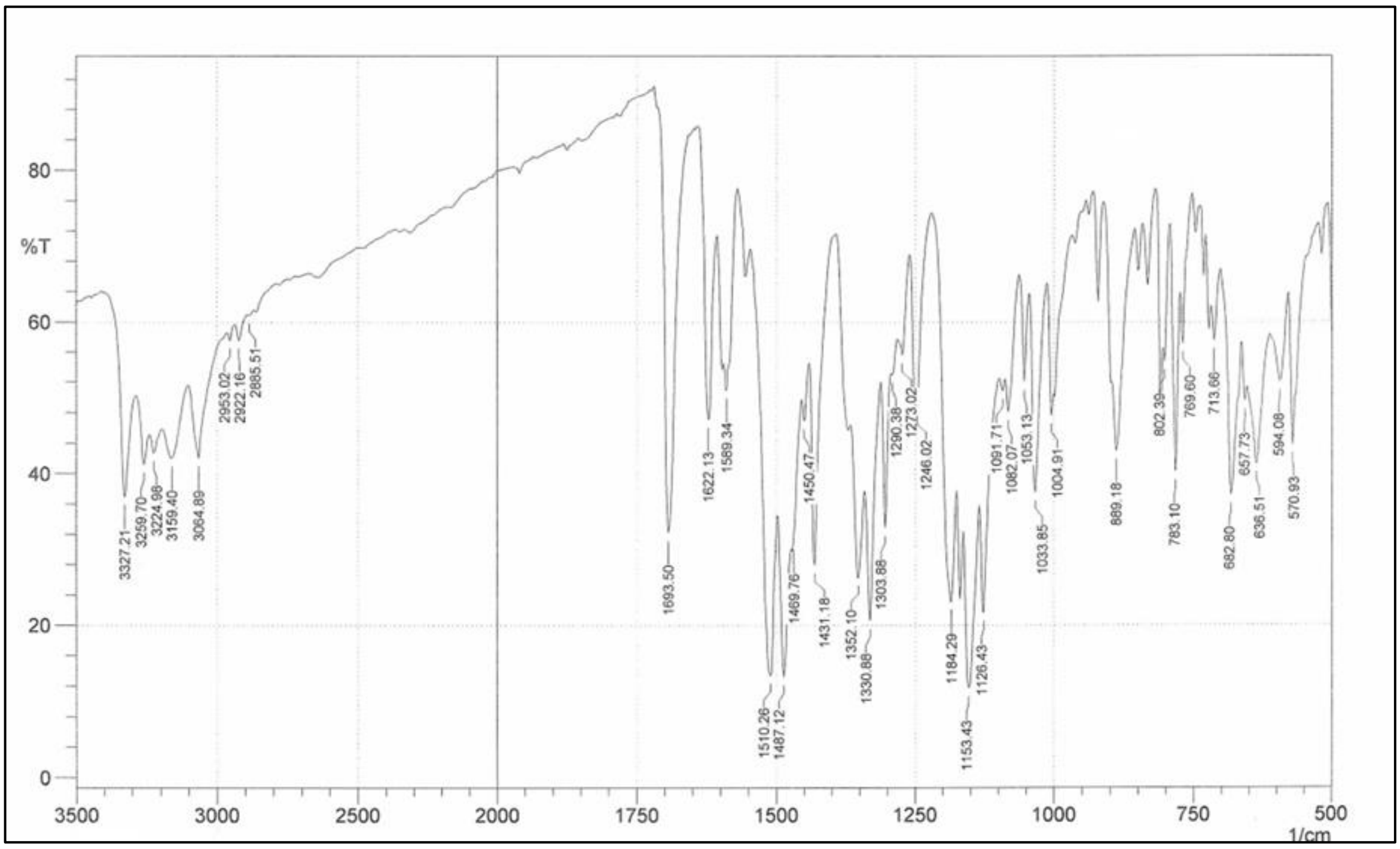

Figure S7: IR spectrum of compound **6c**

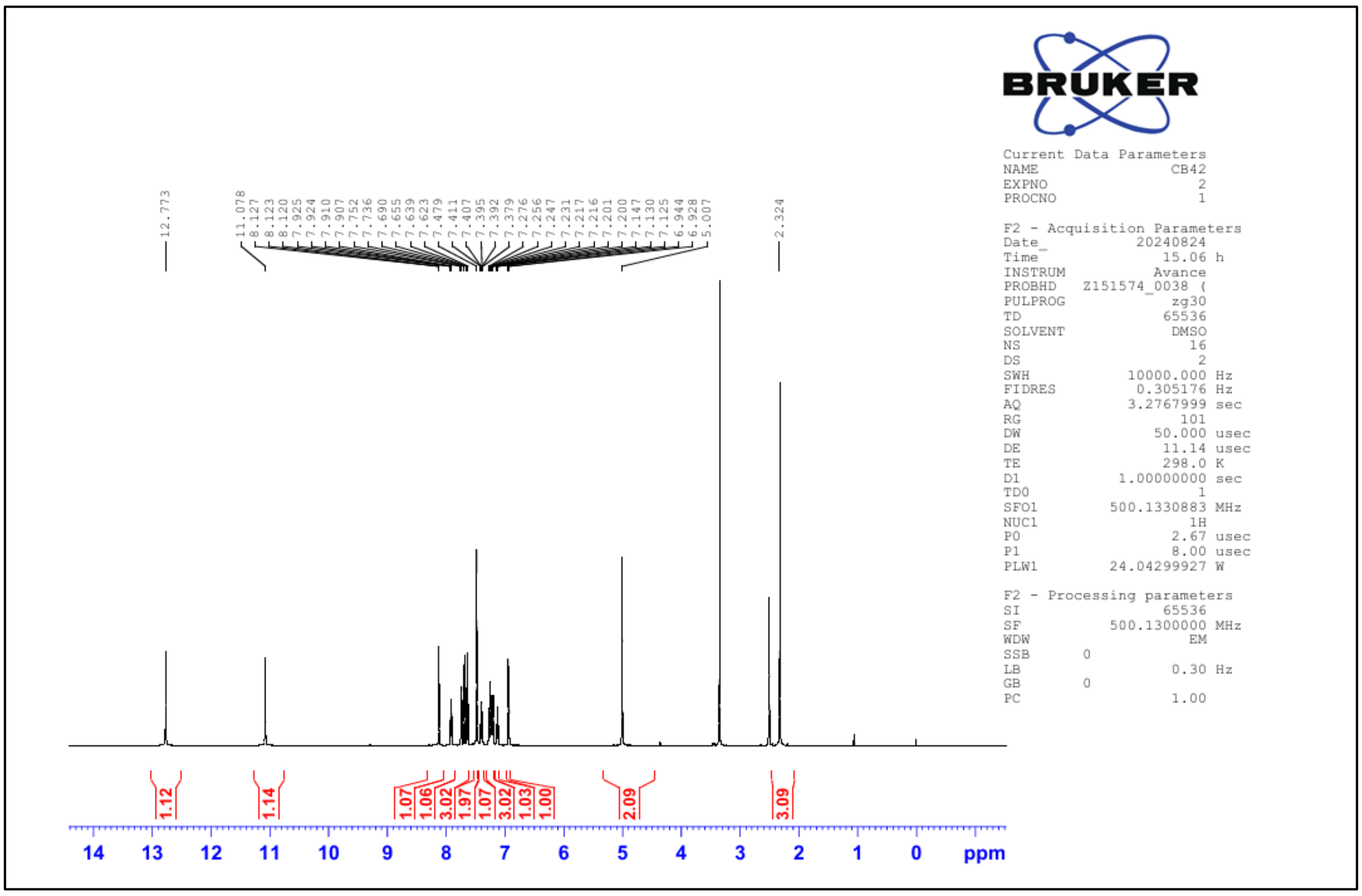

Figure S8: ^{1}H NMR of compound **6c**

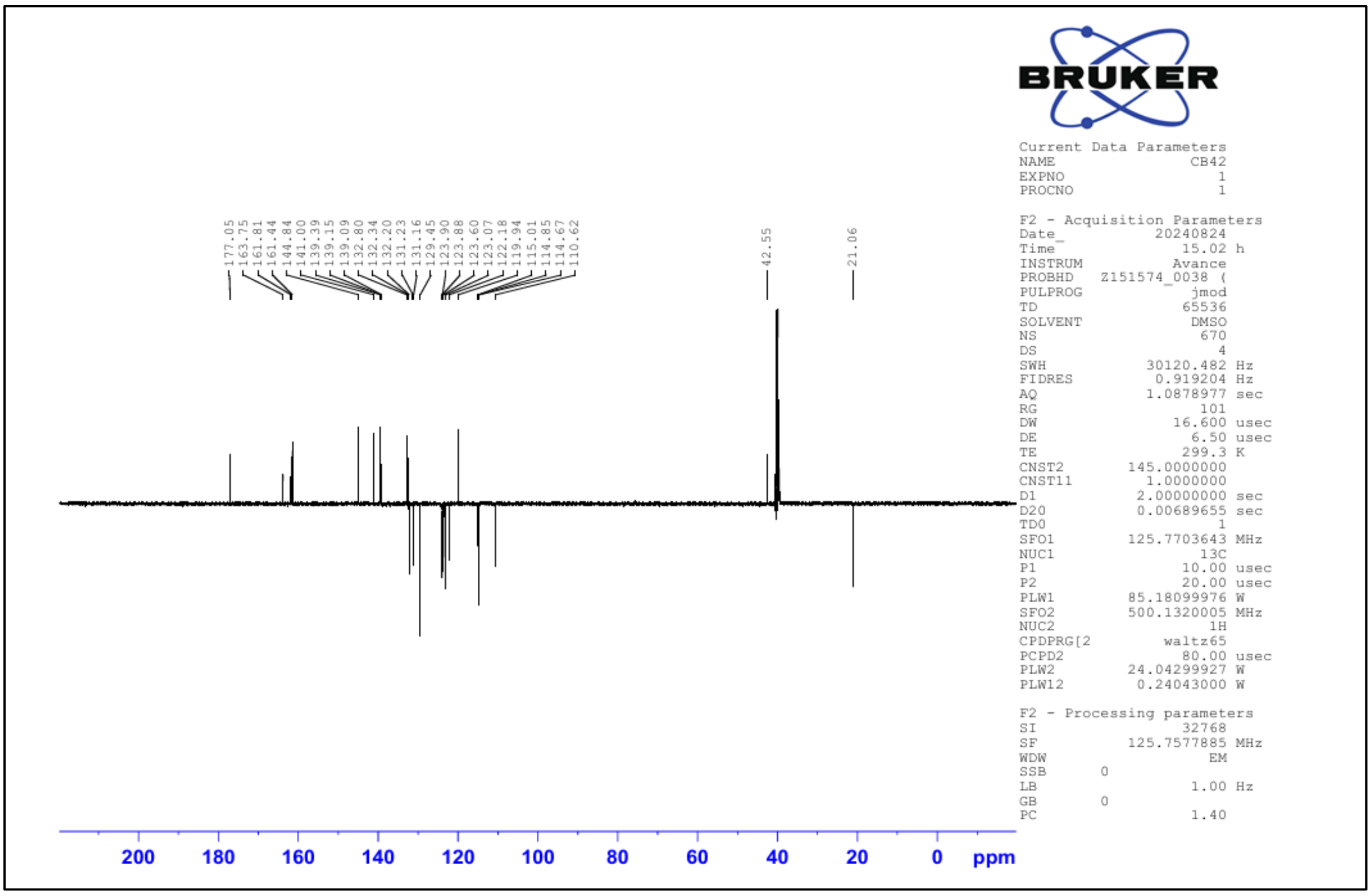

Figure S9: ^{13}C NMR of compound **6c** (APT)

1-(4-Fluorobenzyl)-5-methyl-2-oxoindolin-3-ylidene)-4-(3-sulfamoylphenyl)thiosemicarbazide (6d)

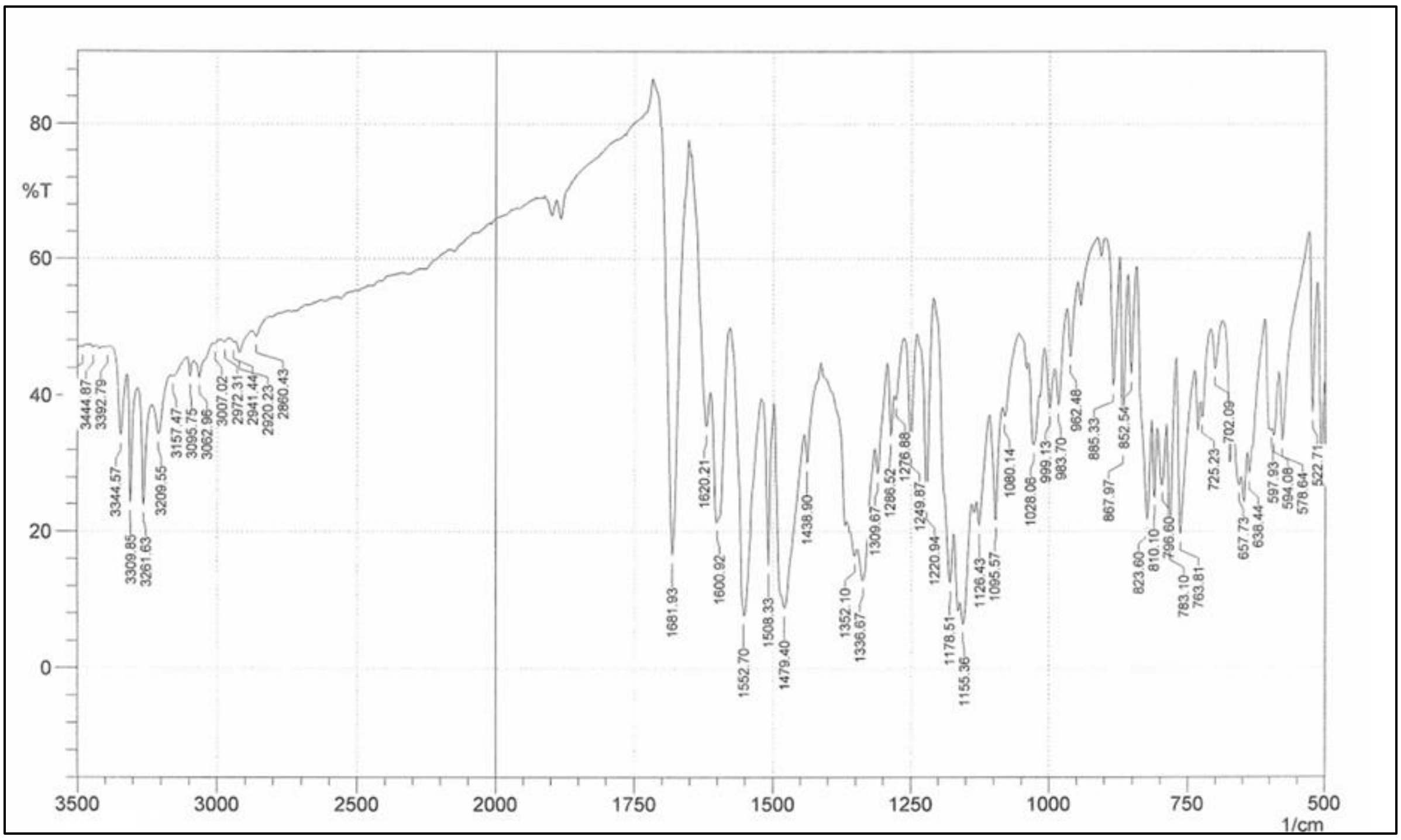

Figure S10: IR spectrum of compound **6d**

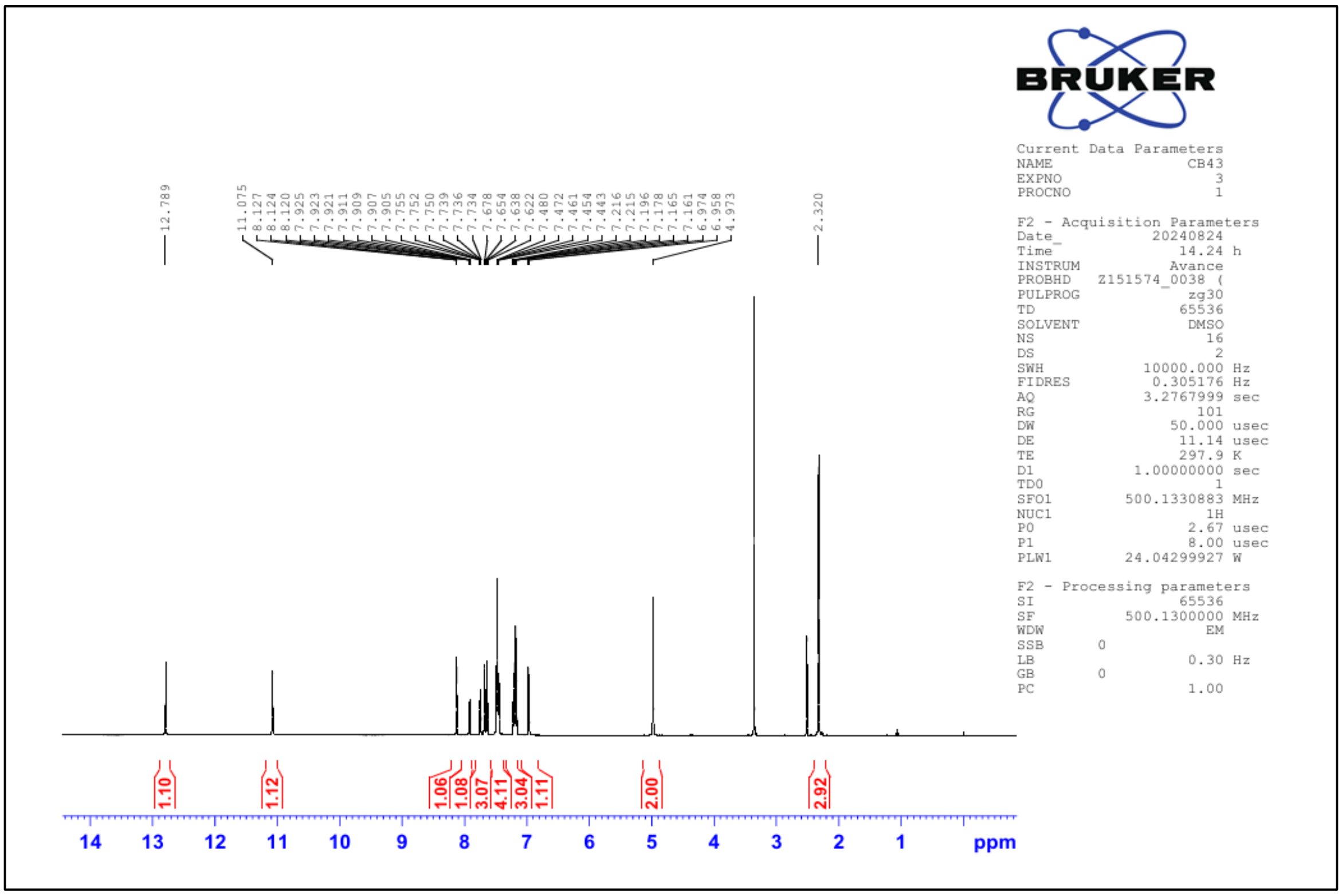

Figure S11: ^{1}H NMR of compound **6d**

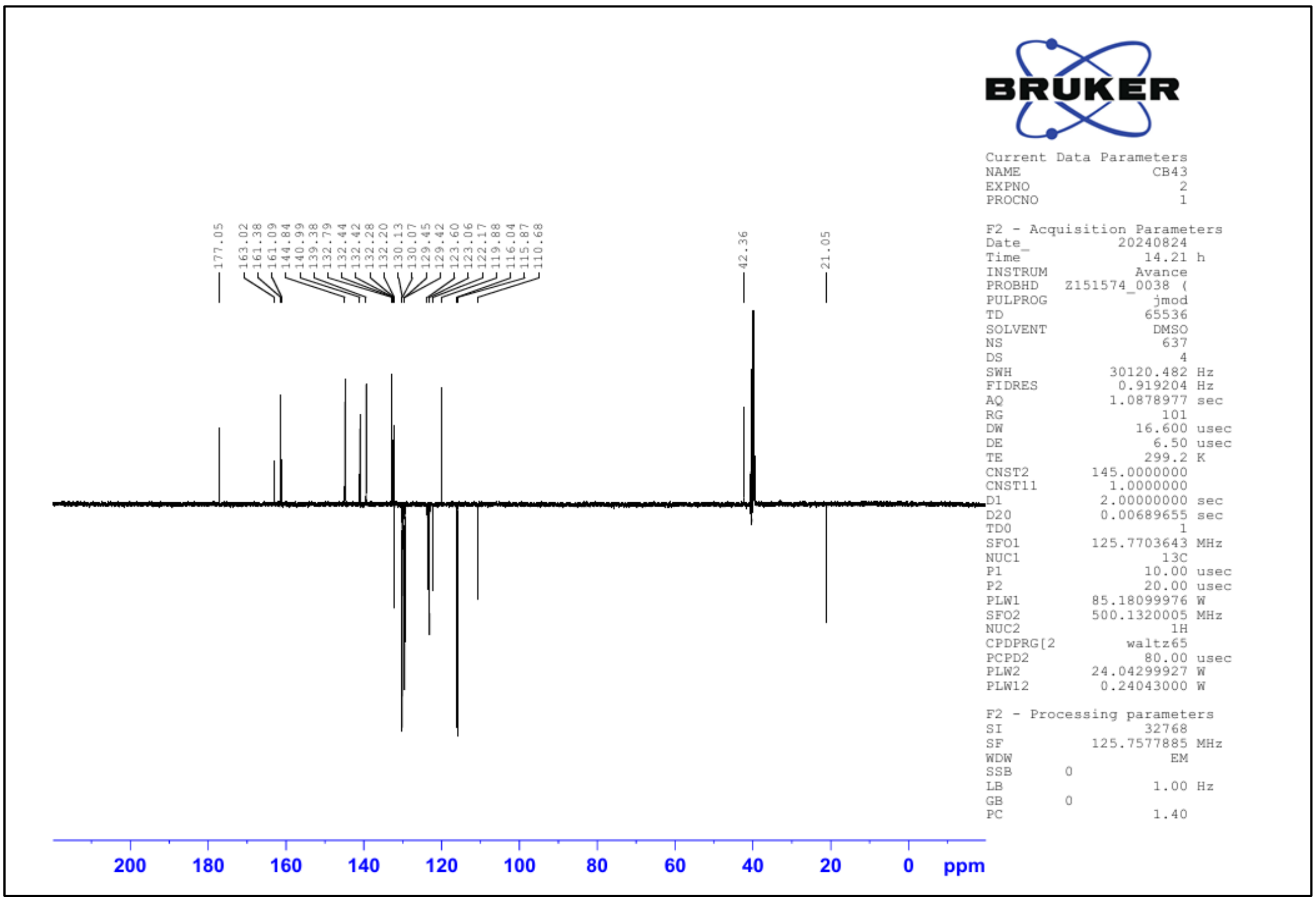

Figure S12: ^{13}C NMR of compound **6d** (APT)

1-(3-Chlorobenzyl)-5-methyl-2-oxoindolin-3-ylidene)-4-(3-sulfamoylphenyl)thiosemicarbazide (6e)

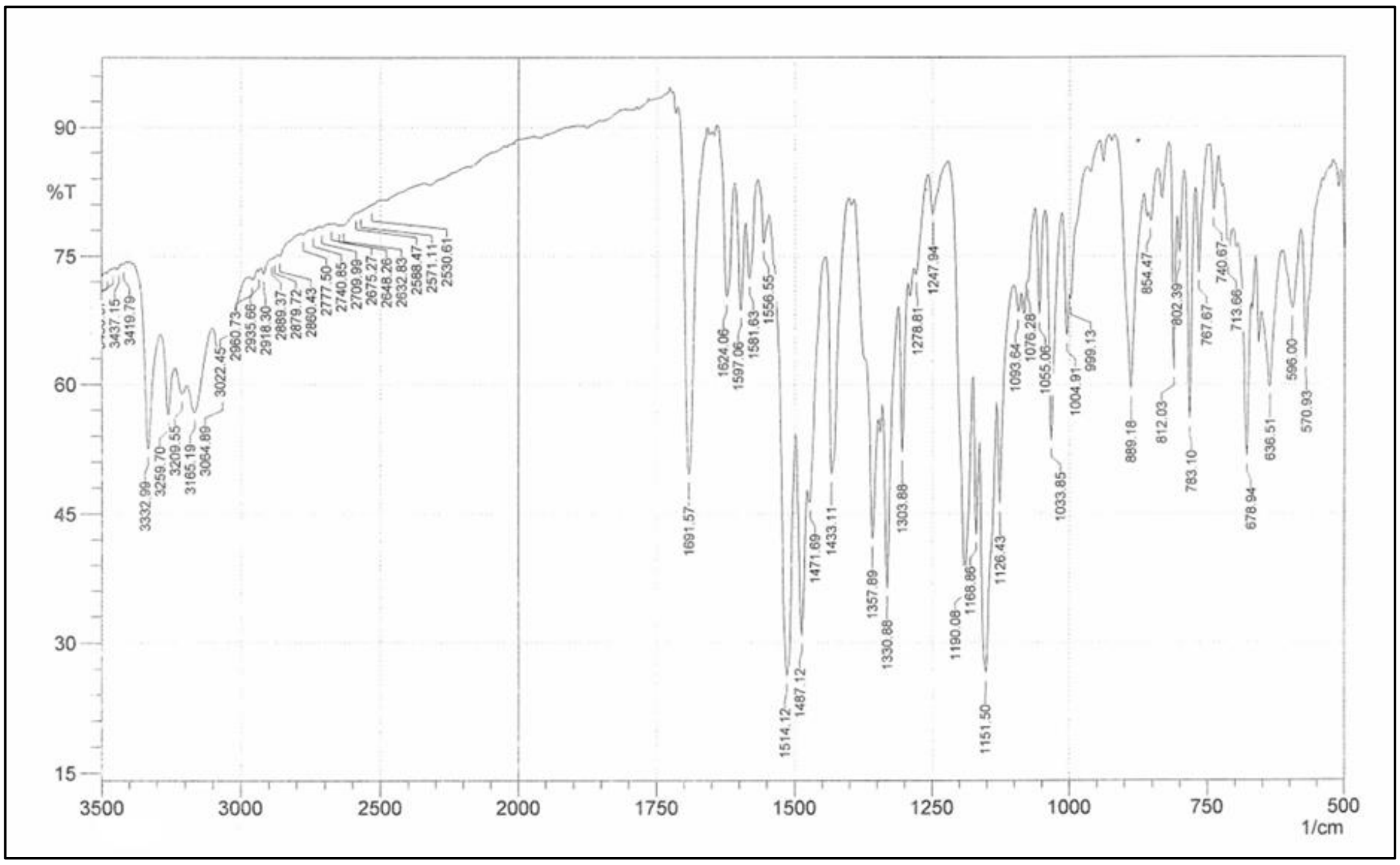

Figure S13: IR spectrum of compound **6e**

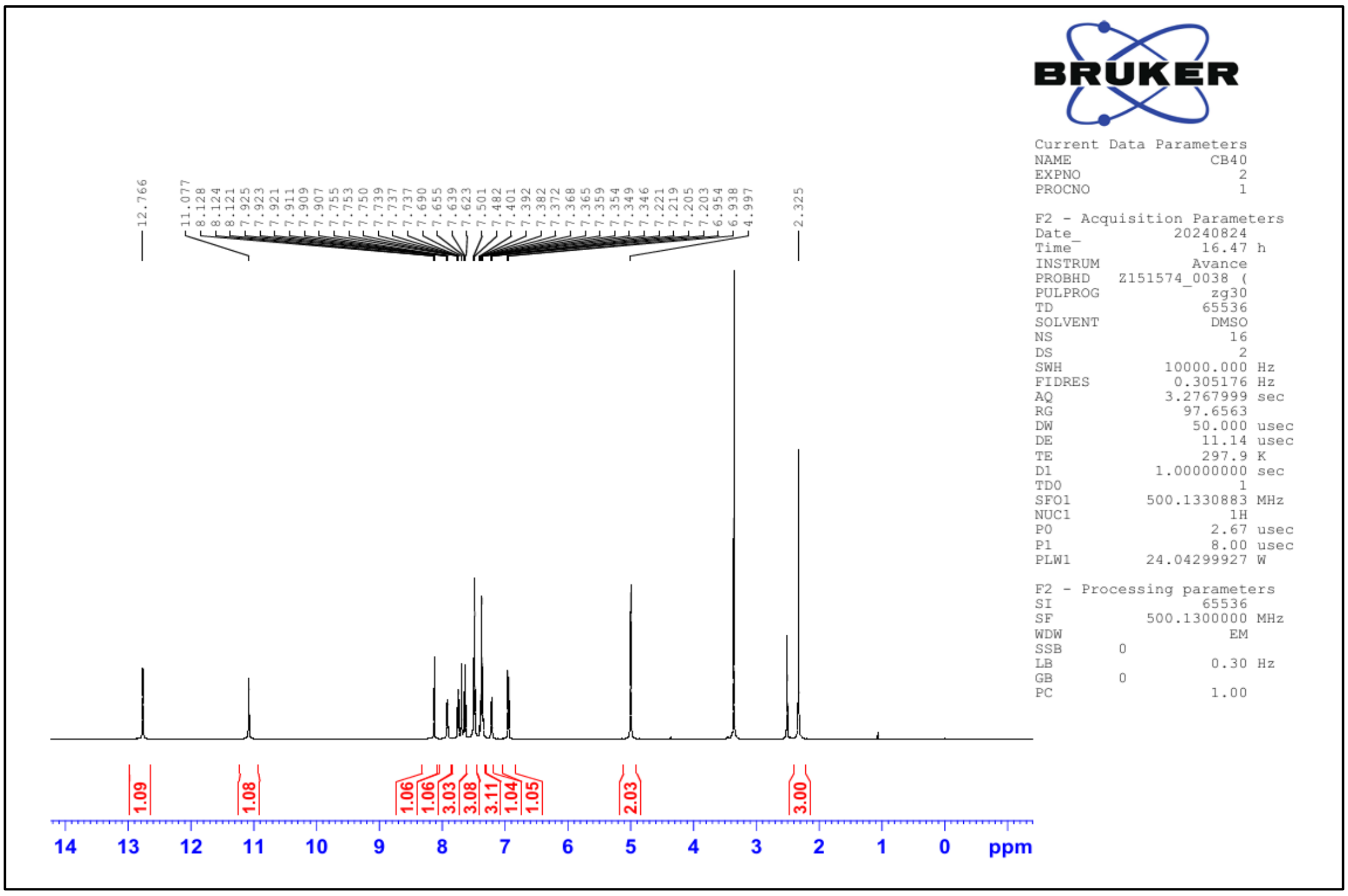

Figure S14: ^{1}H NMR of compound **6e**

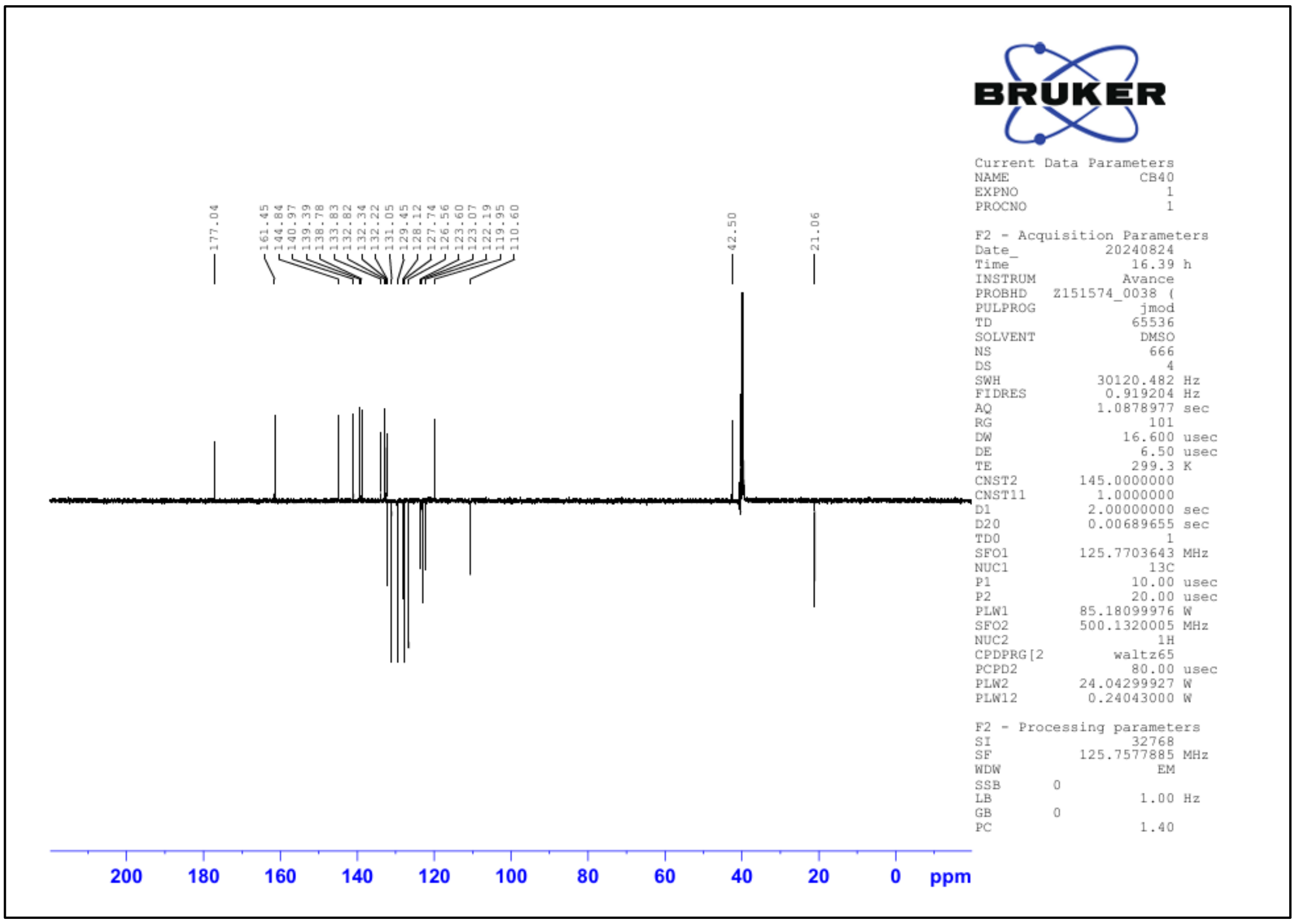

Figure S15: ^{13}C NMR of compound **6e** (APT)

1-(4-Chlorobenzyl)-5-methyl-2-oxoindolin-3-ylidene)-4-(3-sulfamoylphenyl)thiosemicarbazide (6f)

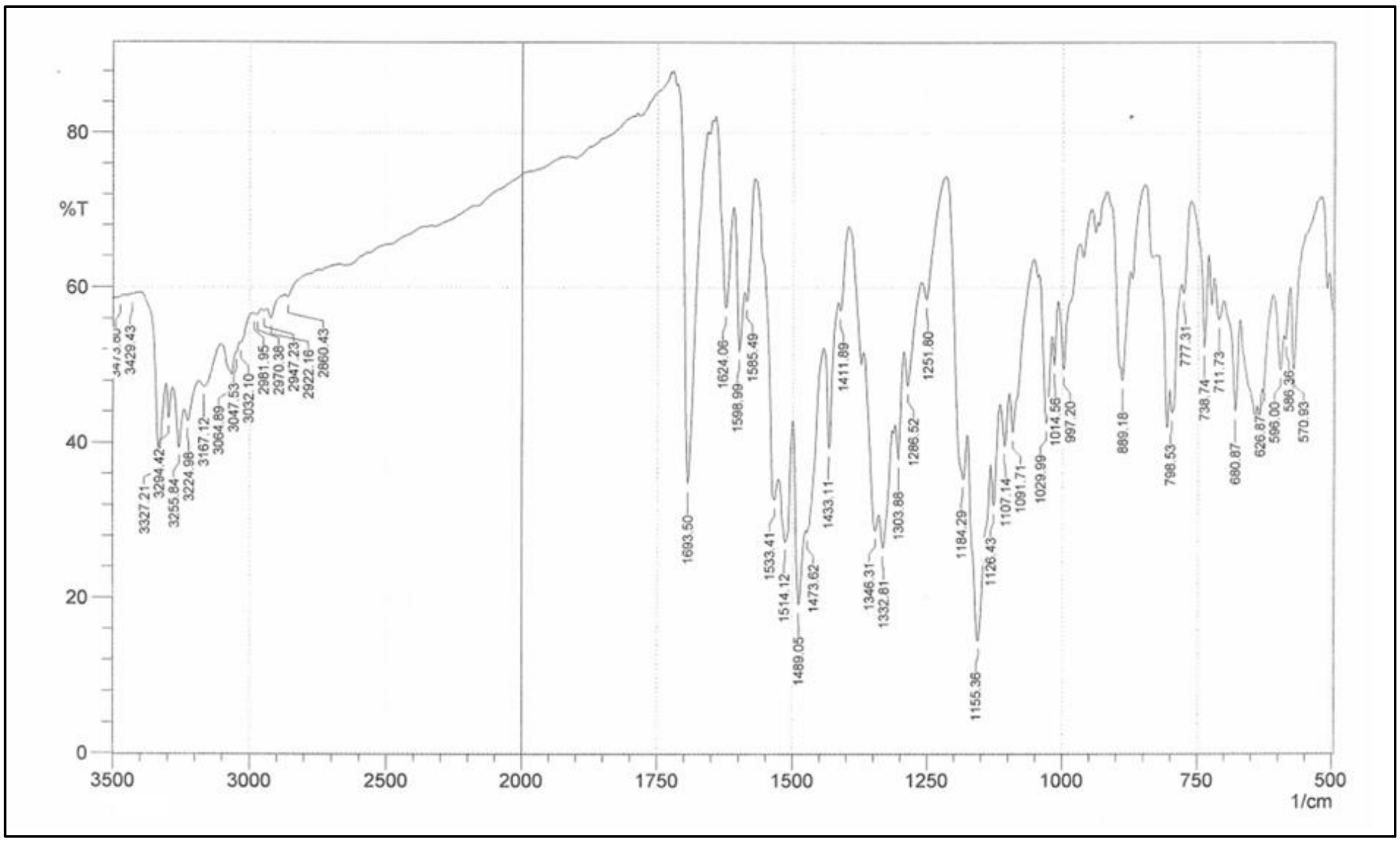

Figure S16: IR spectrum of compound **6f**

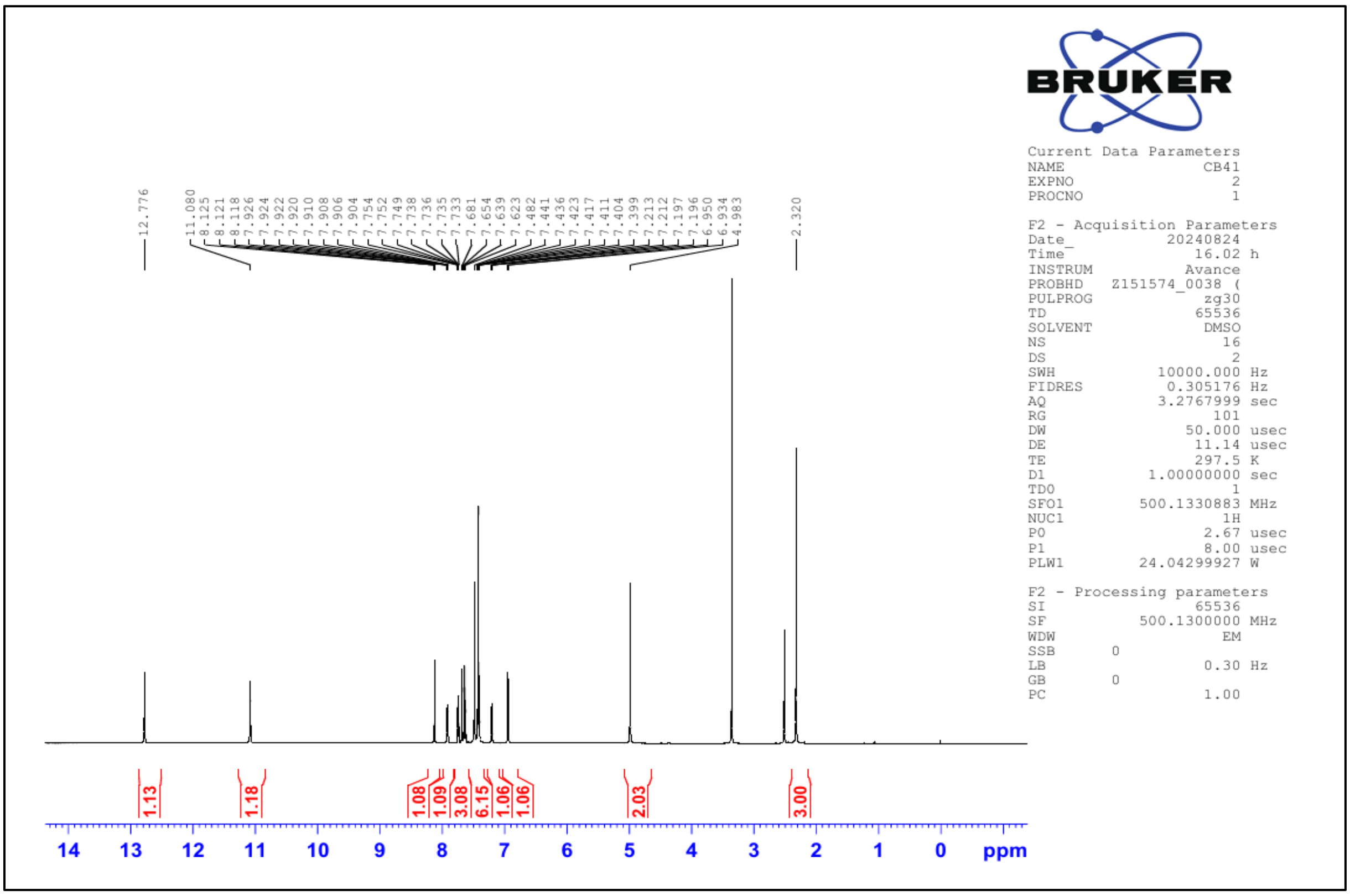

Figure S17: ^{1}H NMR of compound **6f**

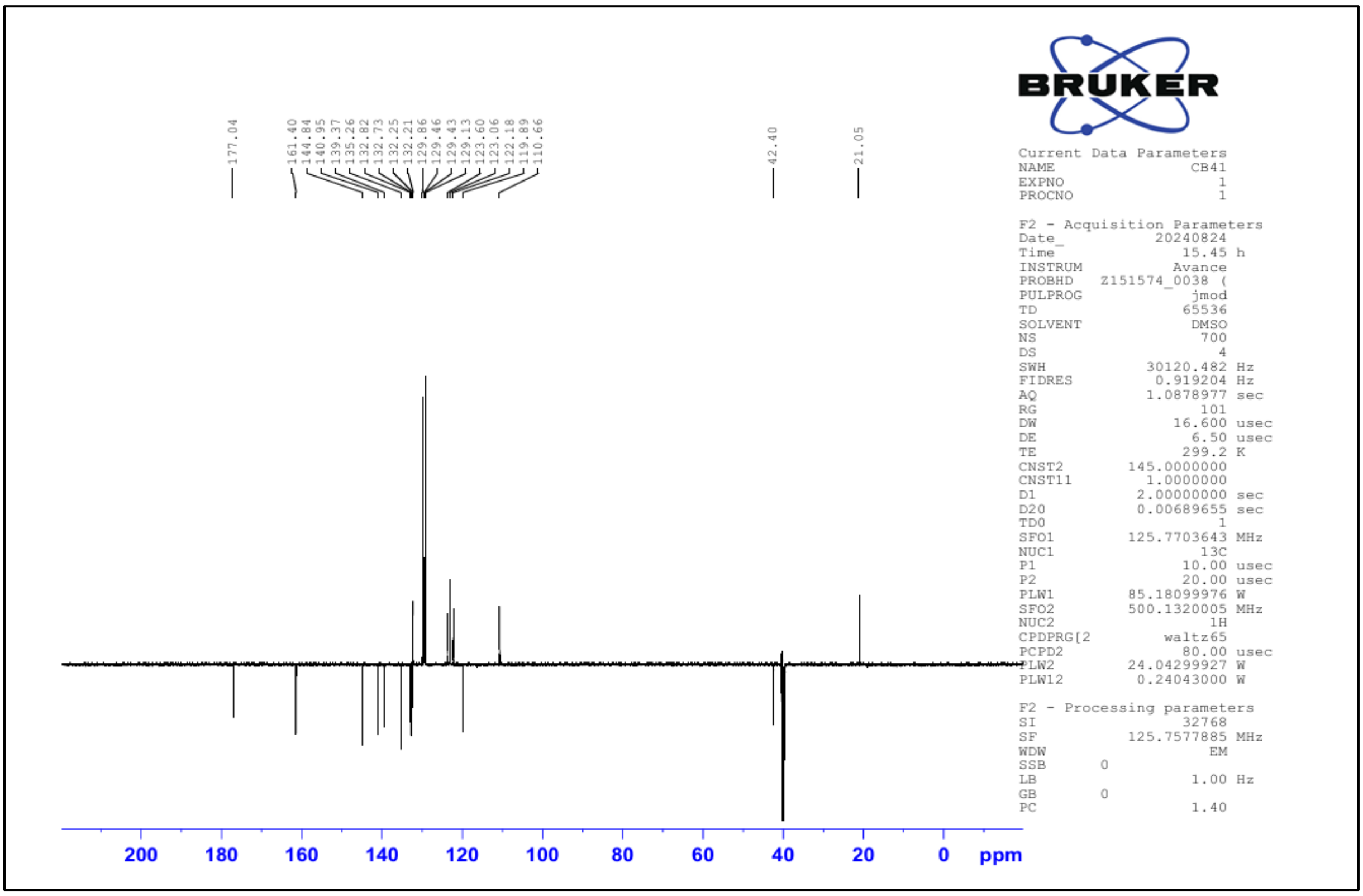

Figure S18: ^{13}C NMR of compound **6f** (APT)

1-(3-Methylbenzyl)-5-chloro-2-oxoindolin-3-ylidene)-4-(3-sulfamoylphenyl)thiosemicarbazide (6g)

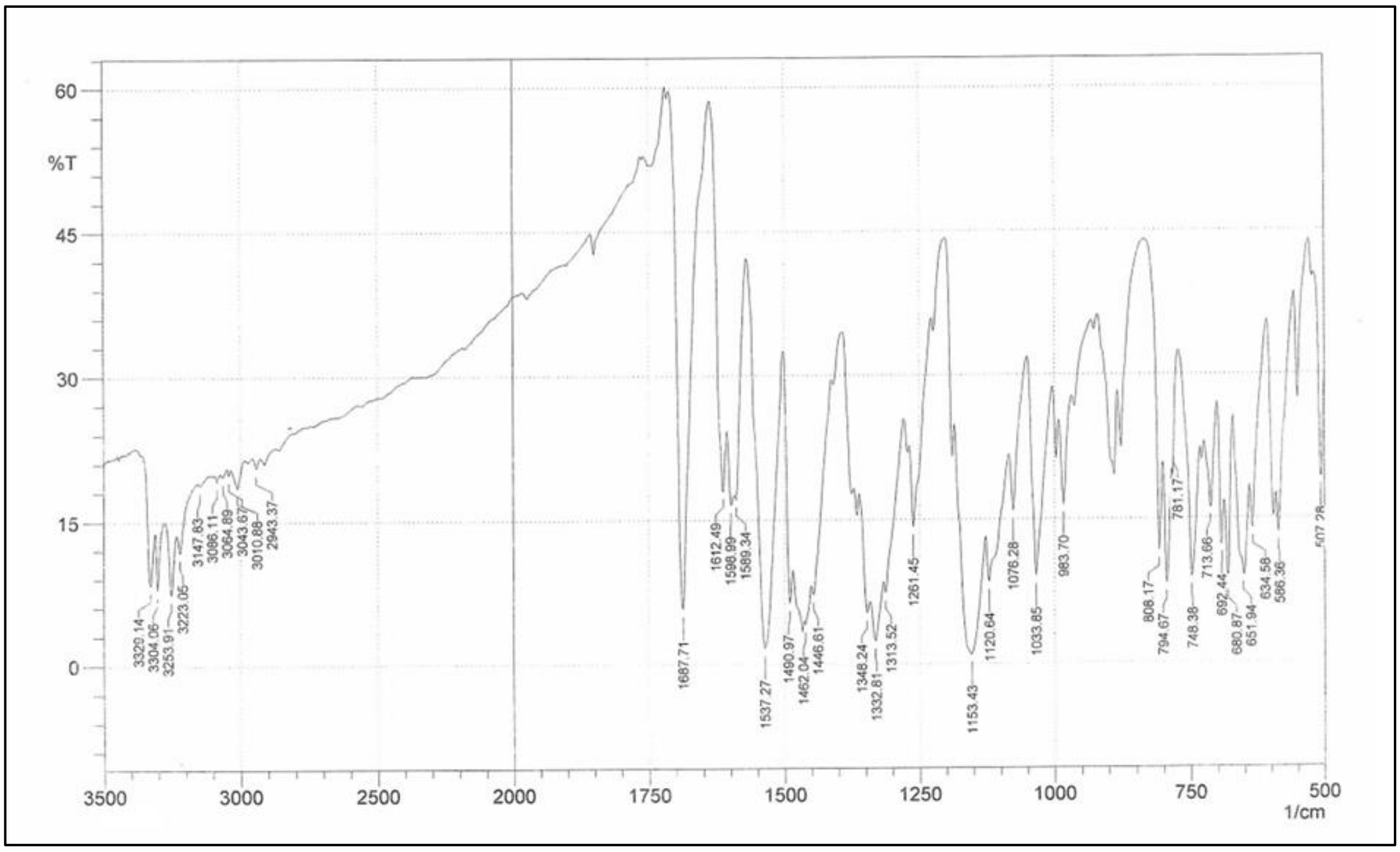

Figure S19: IR spectrum of compound **6g**

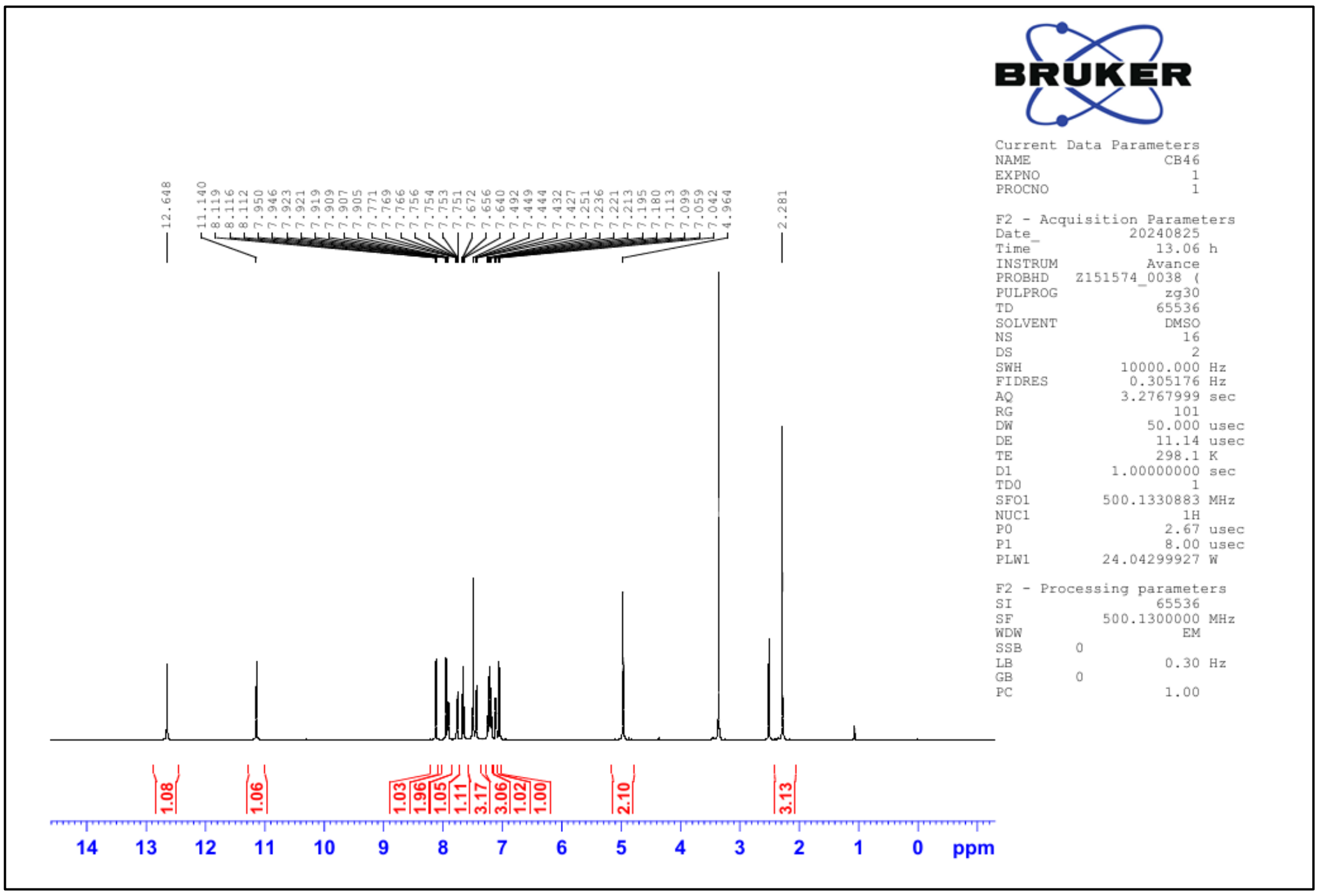

Figure S20: ^{1}H NMR of compound **6g**

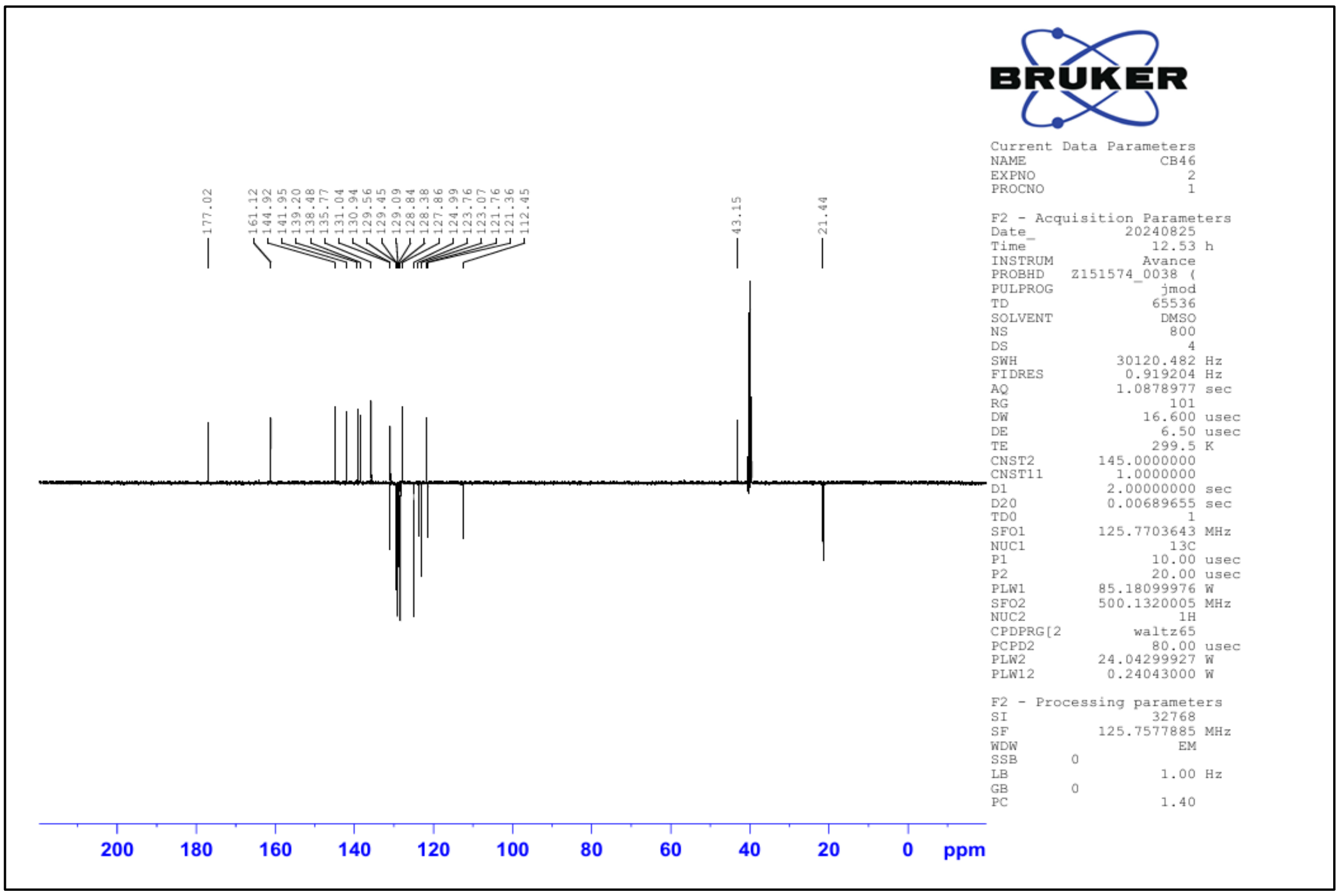

Figure S21: ^{13}C NMR of compound **6g** (APT)

1-(4-Methylbenzyl)-5-chloro-2-oxoindolin-3-ylidene)-4-(3-sulfamoylphenyl)thiosemicarbazide (6h)

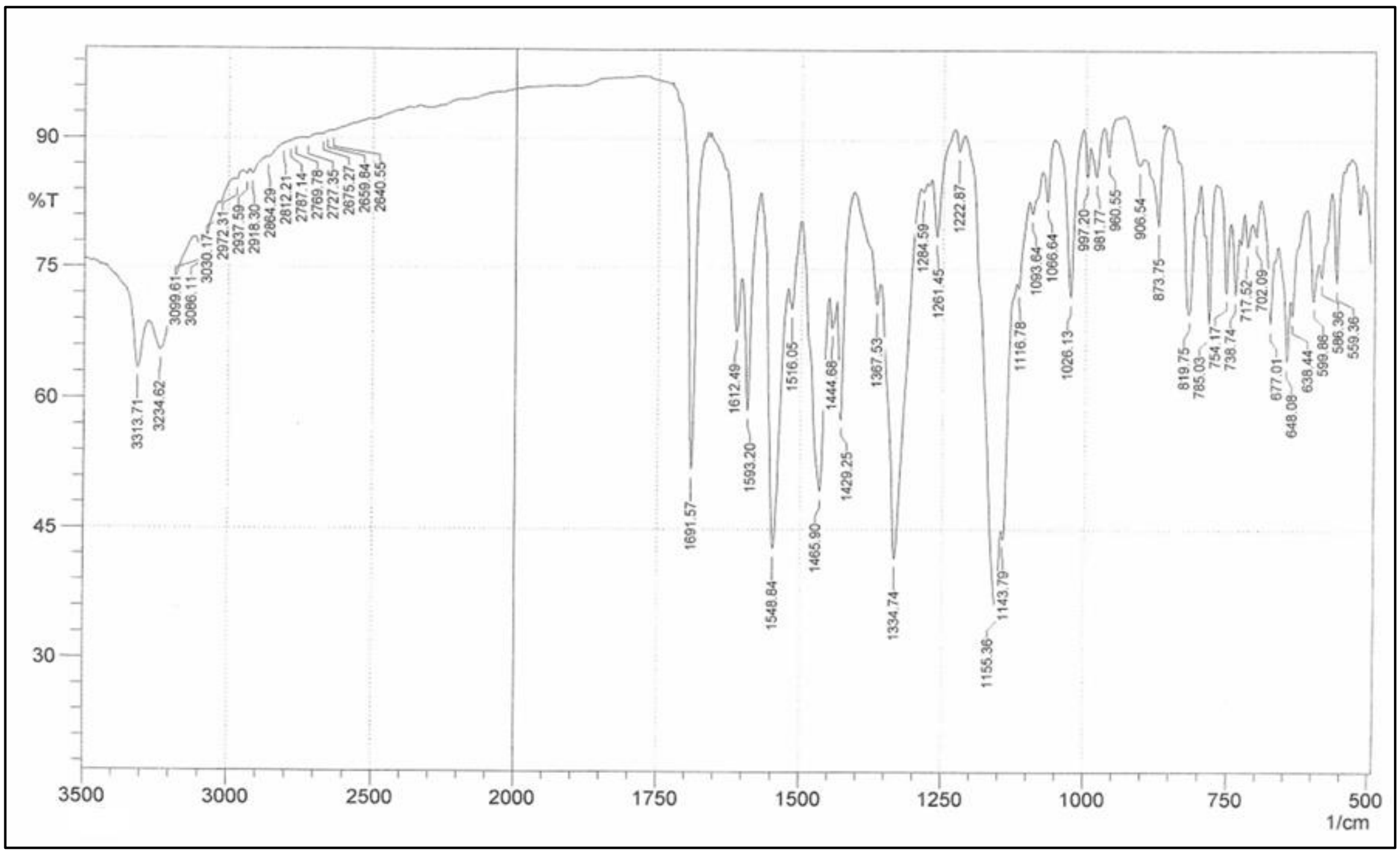

Figure S22: IR spectrum of compound **6h**

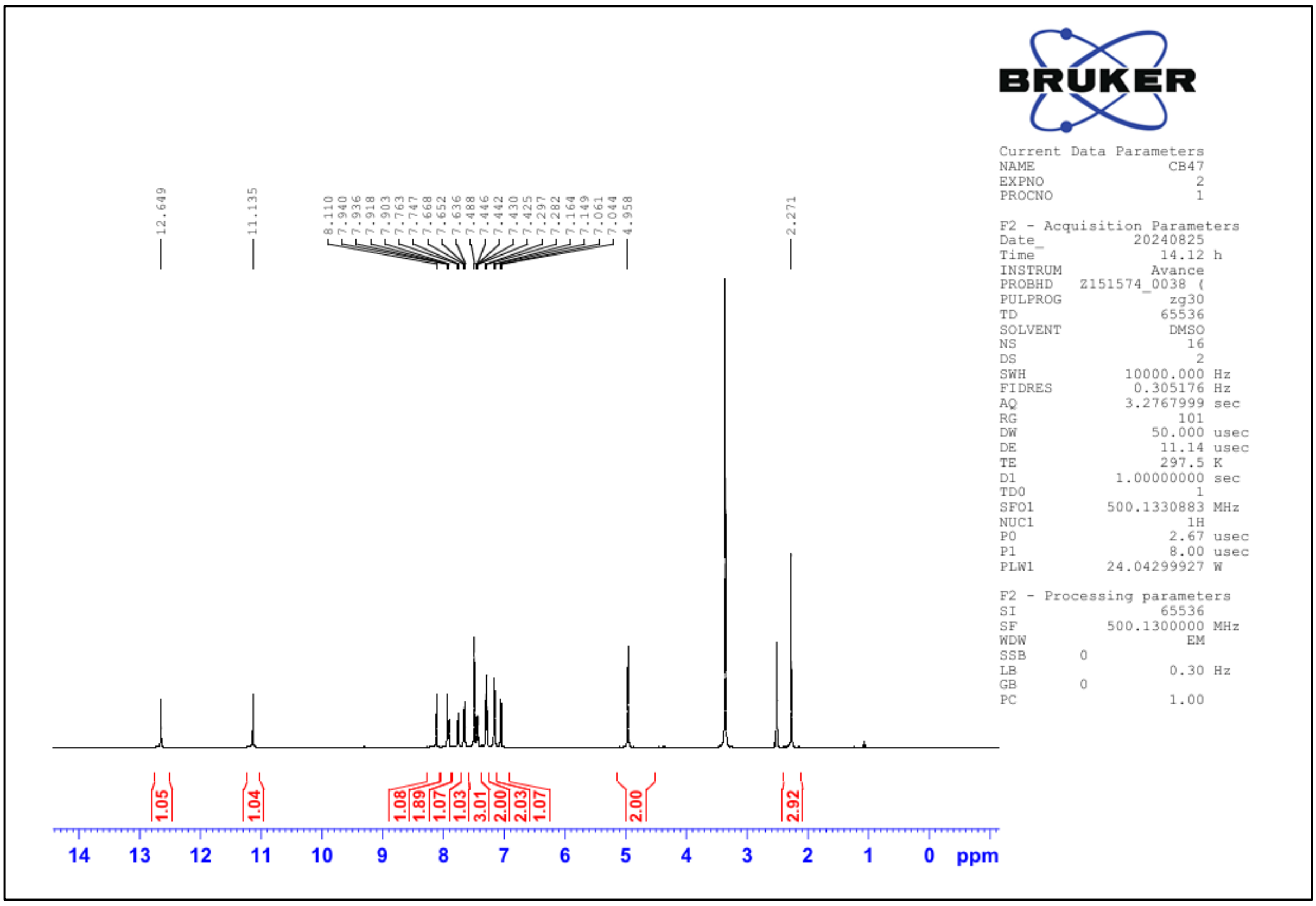

Figure S23: ^{1}H NMR of compound **6h**

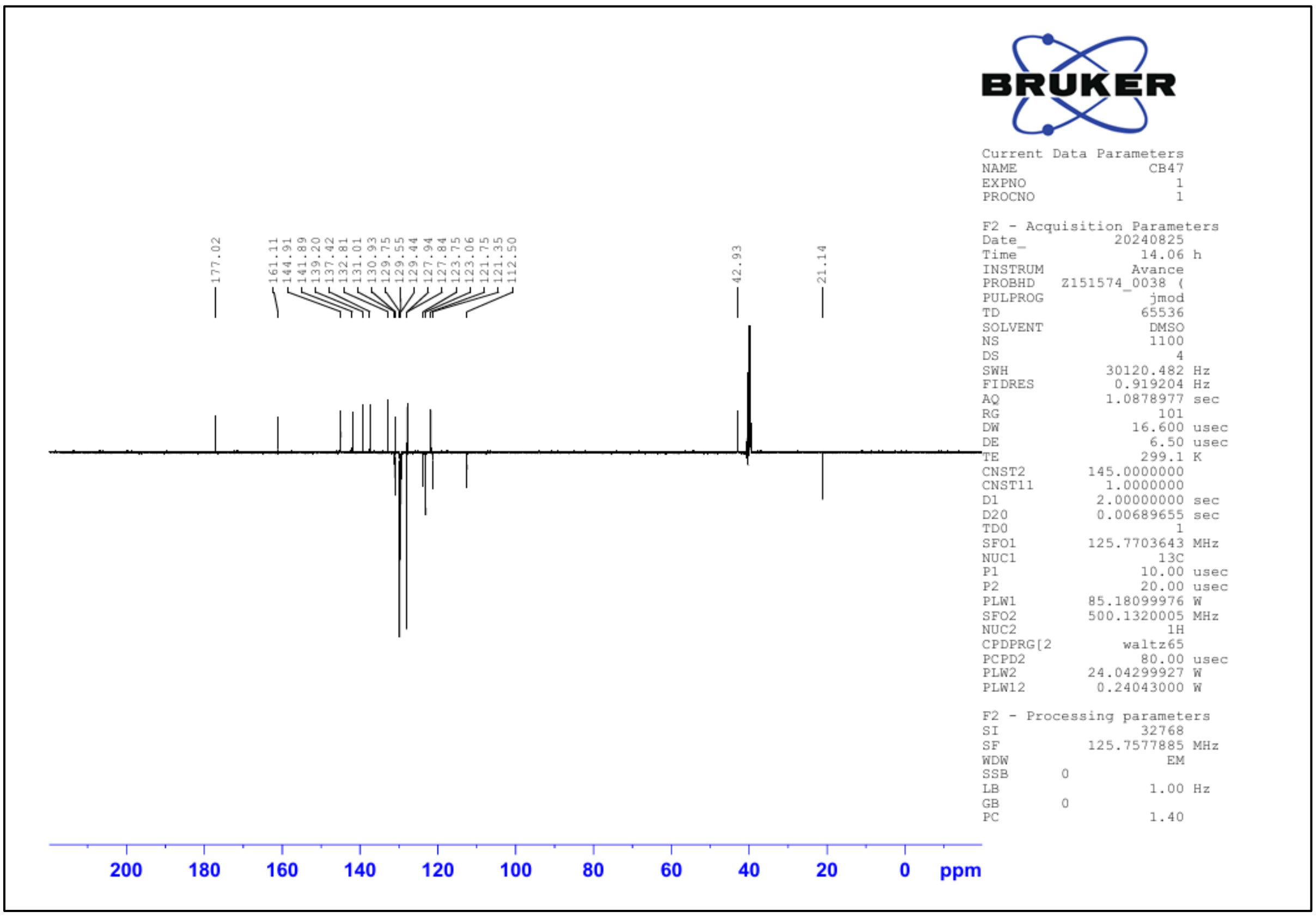

Figure S24: ^{13}C NMR of compound **6h** (APT)

1-(3-Fluorobenzyl)-5-chloro-2-oxoindolin-3-ylidene)-4-(3-sulfamoylphenyl)thiosemicarbazide (6i)

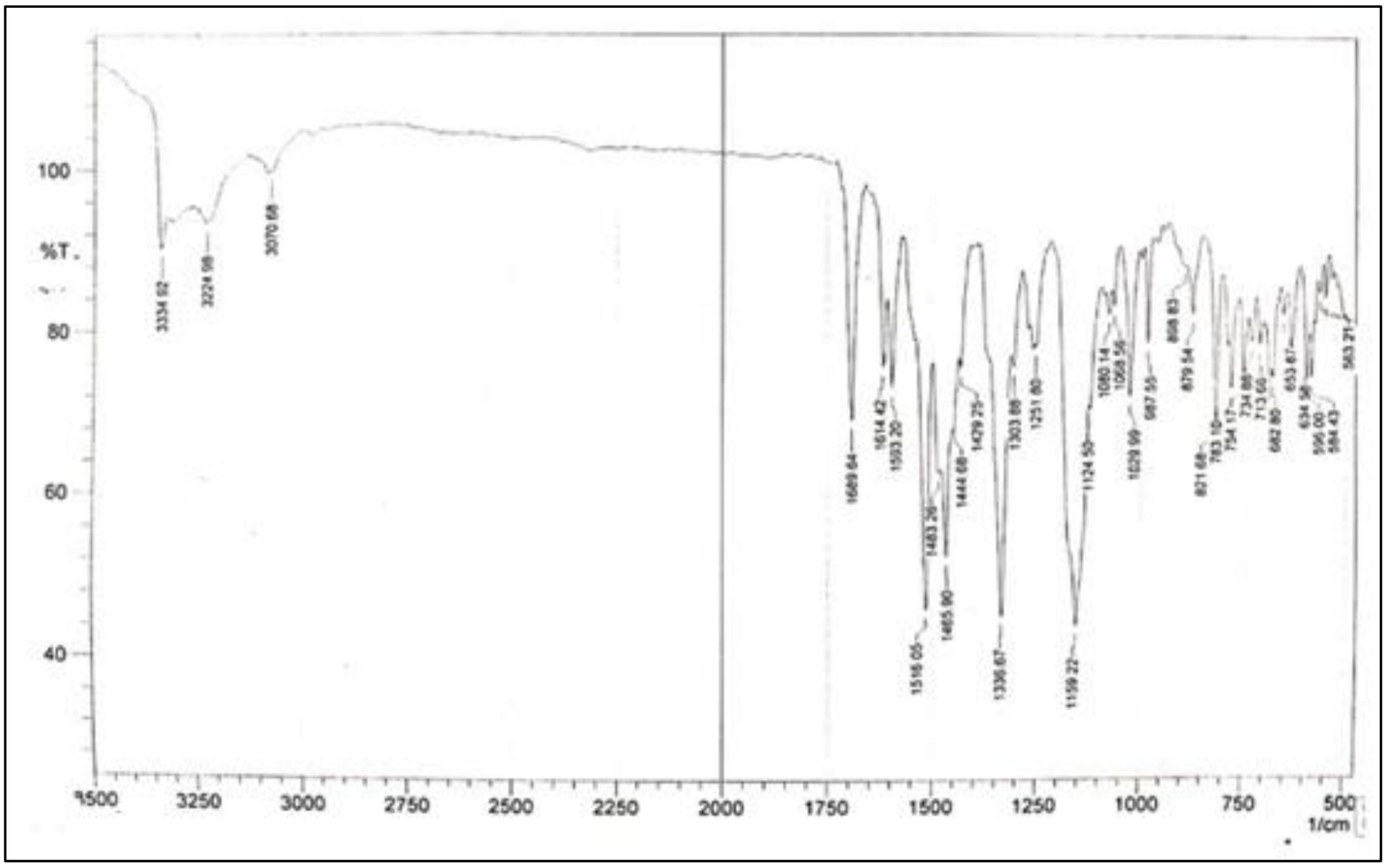

Figure S25: IR spectrum of compound **6i**

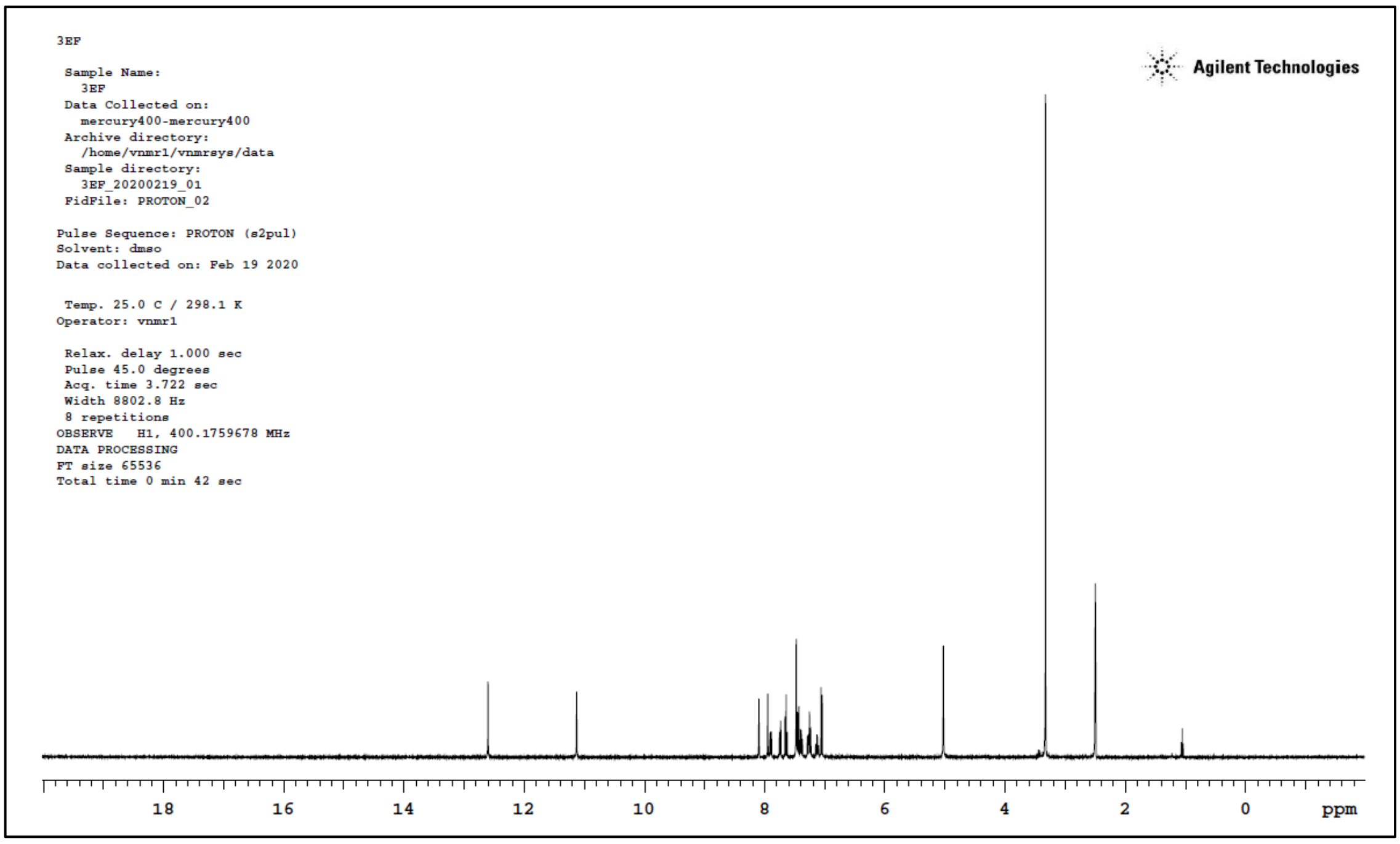

Figure S26: ^{1}H NMR of compound **6i**

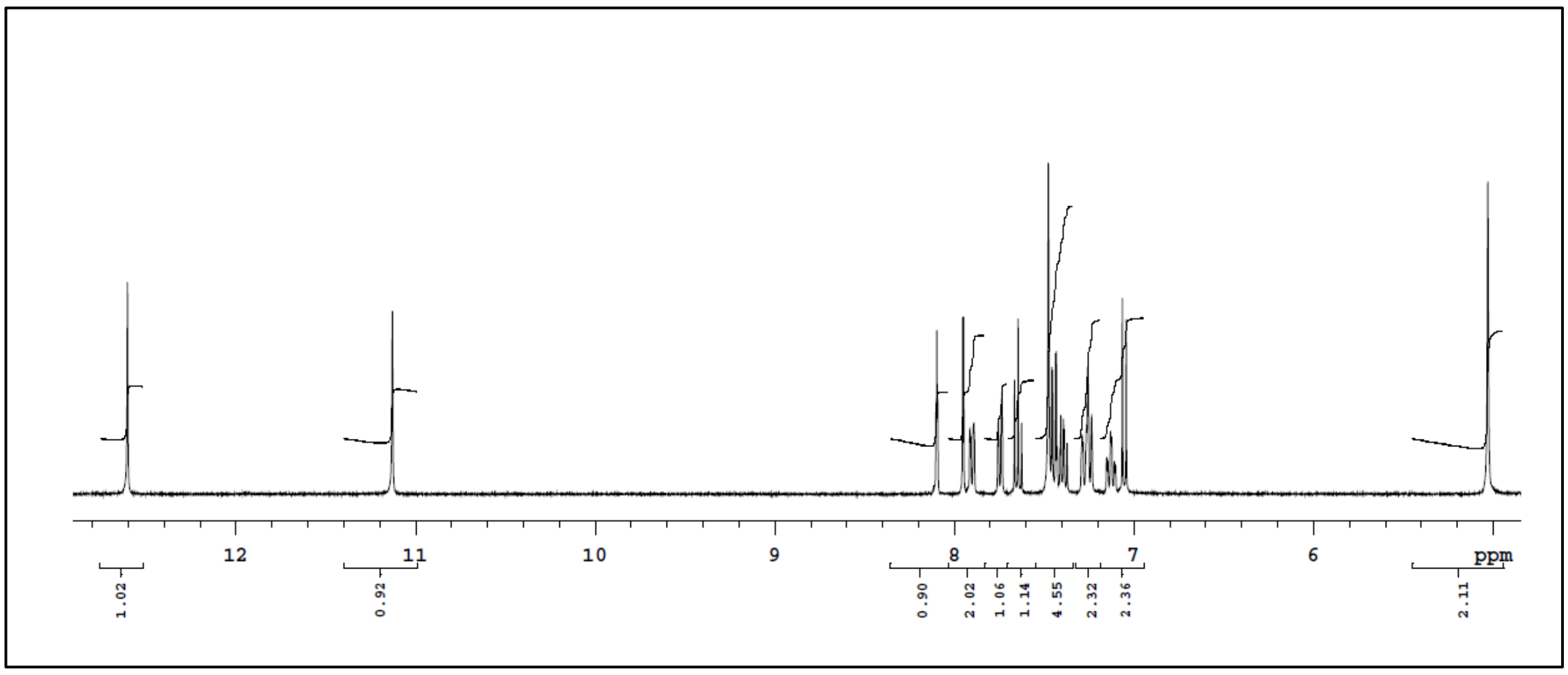

Figure S27: ^{1}H NMR of compound **6i** (6.0 – 13.0 ppm)

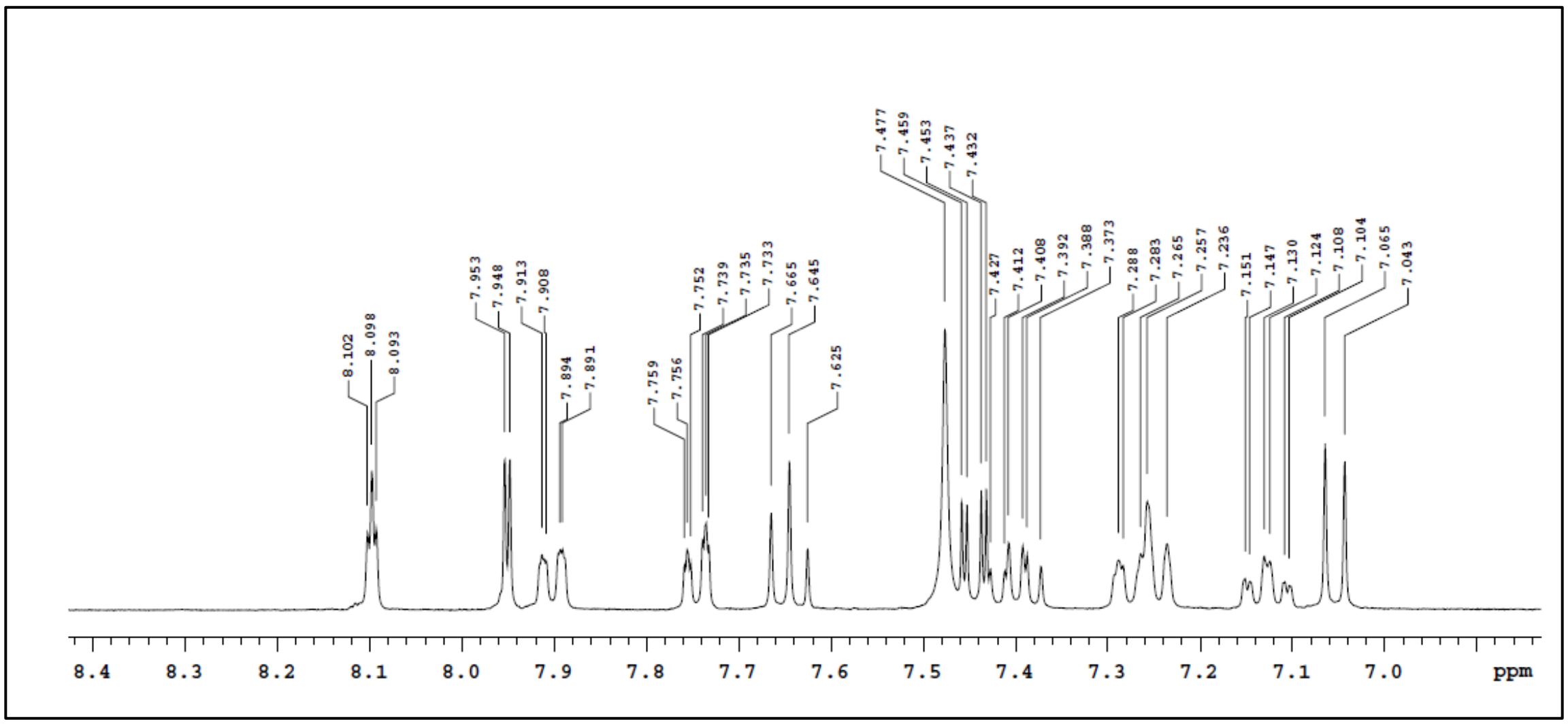

Figure S28: ^{1}H NMR of compound **6i** (6.9 – 8.4 ppm)

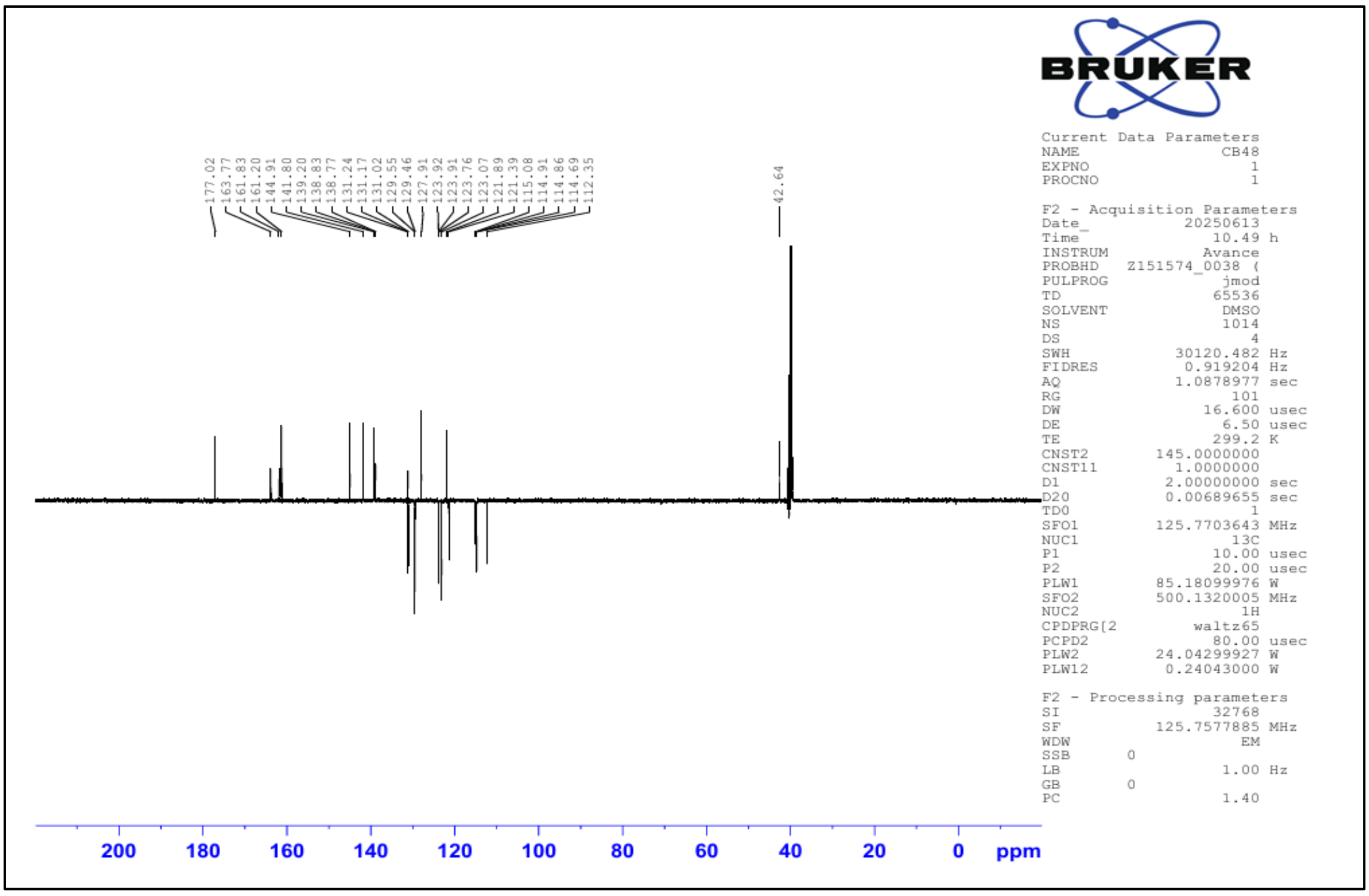

Figure S29: ^{13}C NMR of compound **6i** (APT)

1-(4-Fluorobenzyl)-5-chloro-2-oxoindolin-3-ylidene)-4-(3-sulfamoylphenyl)thiosemicarbazide (6j)

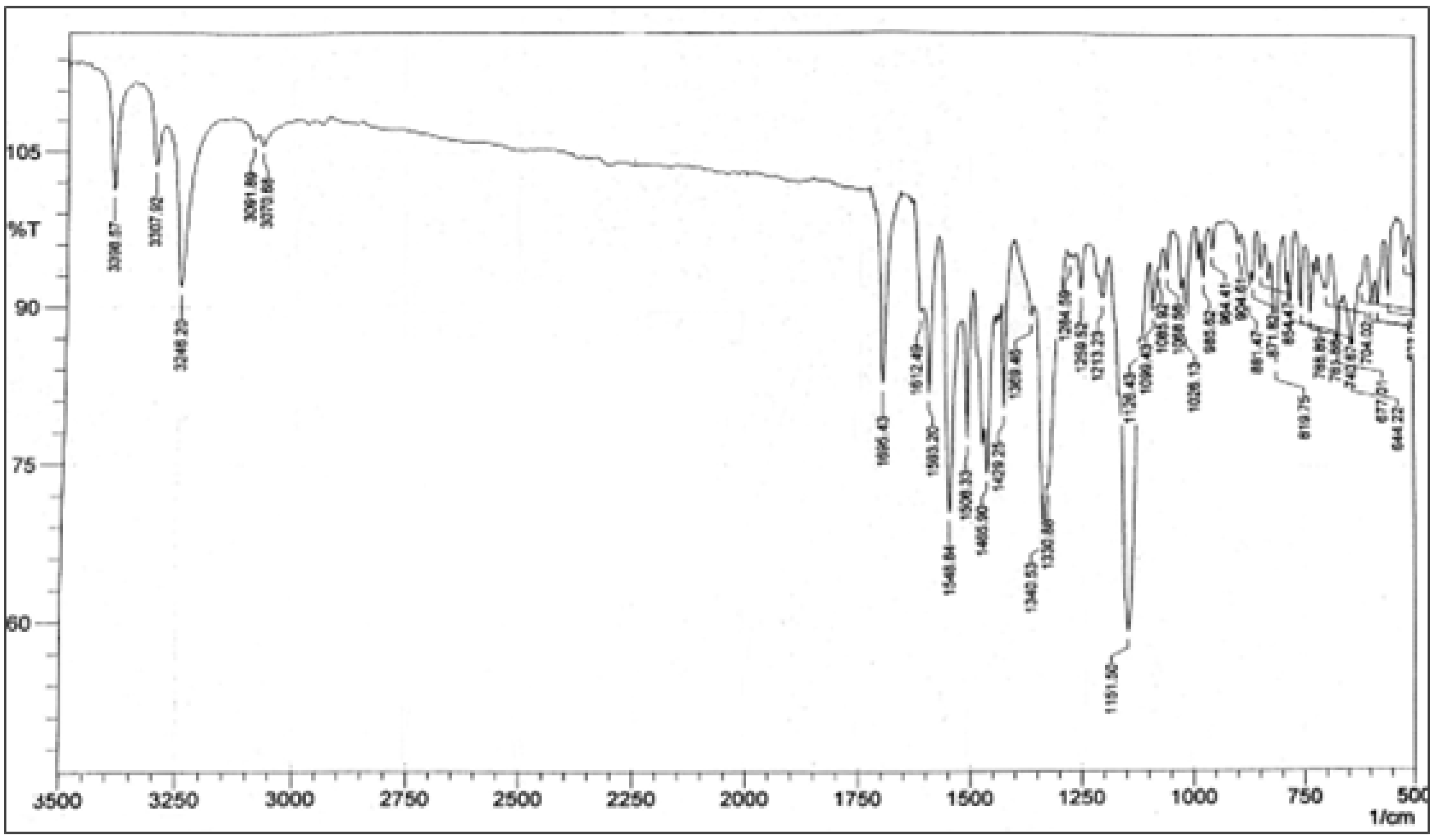

Figure S30: IR spectrum of compound **6j**

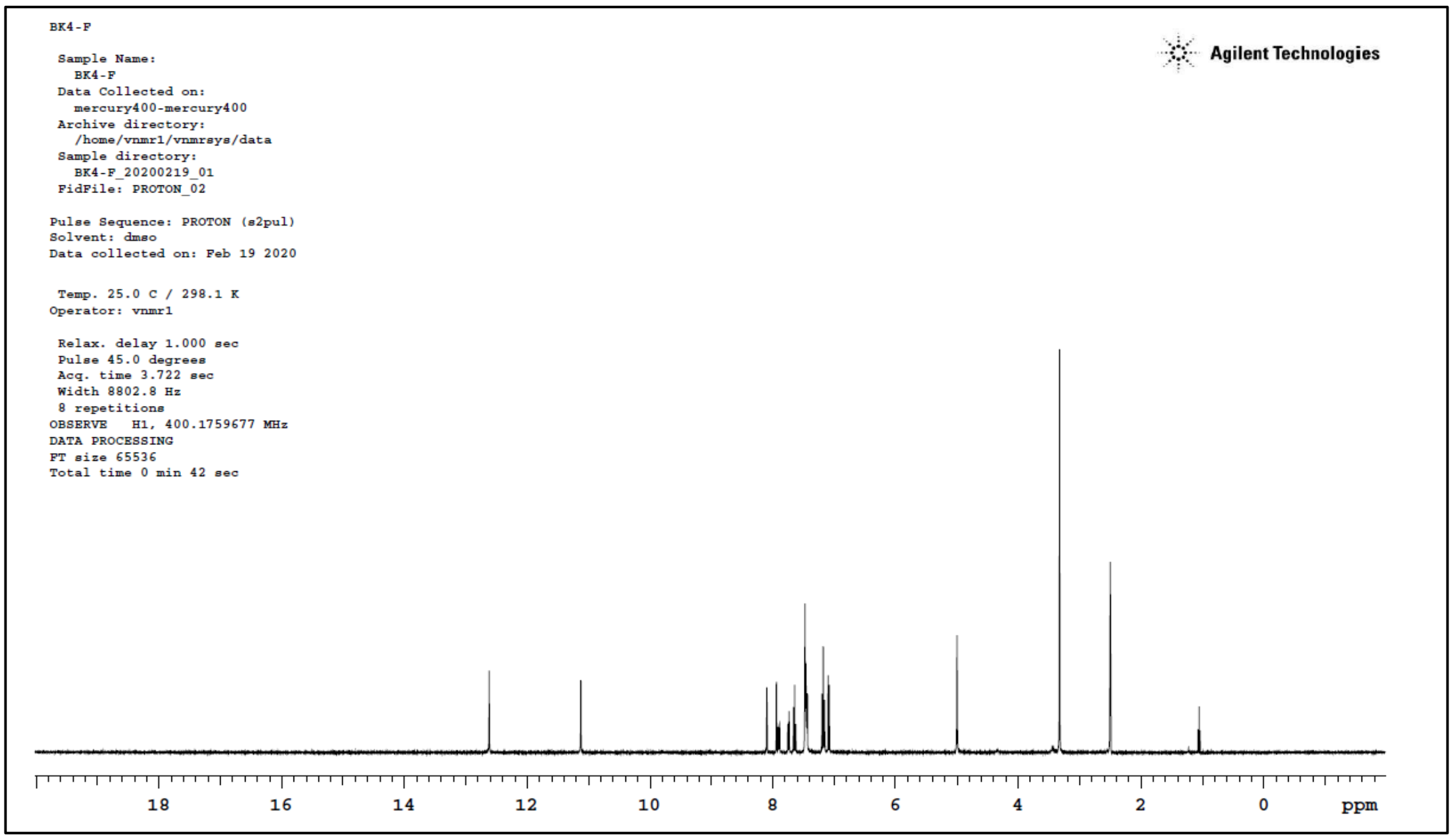

Figure S31: ^{1}H NMR of compound **6j**

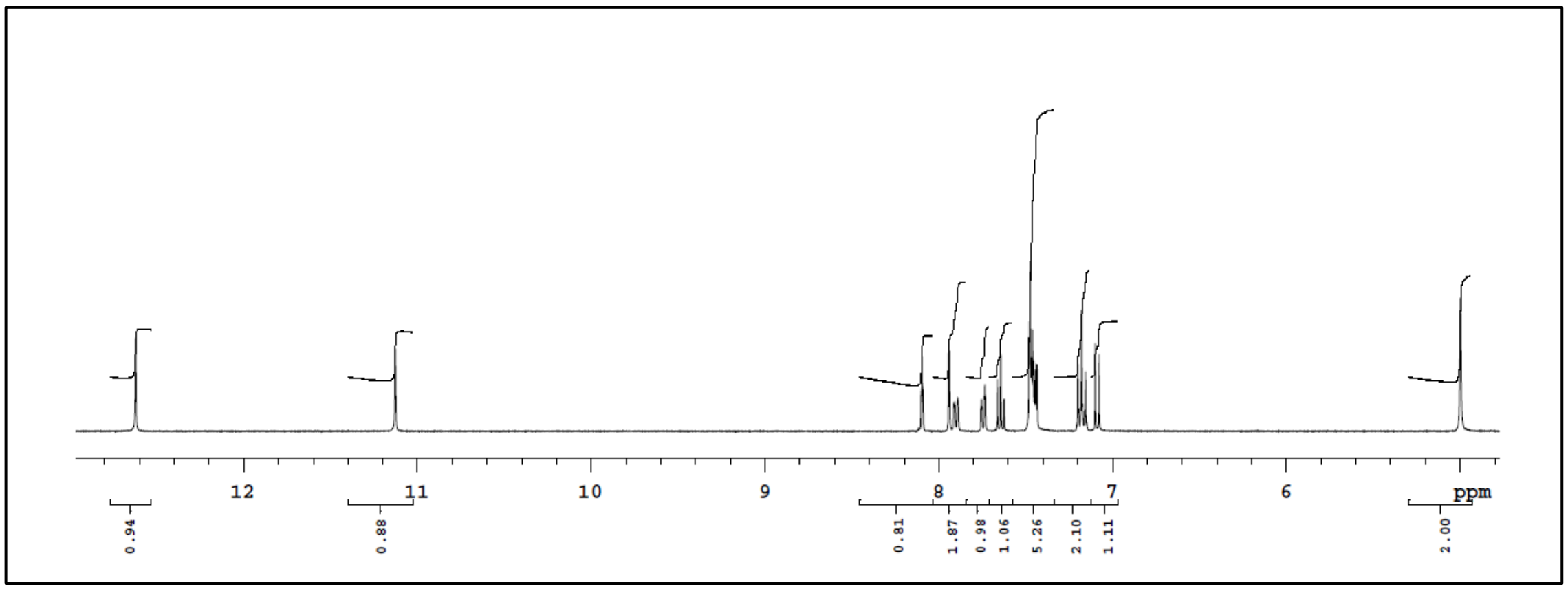

Figure S32: ^{1}H NMR of compound **6j** (5.0 – 13.0 ppm)

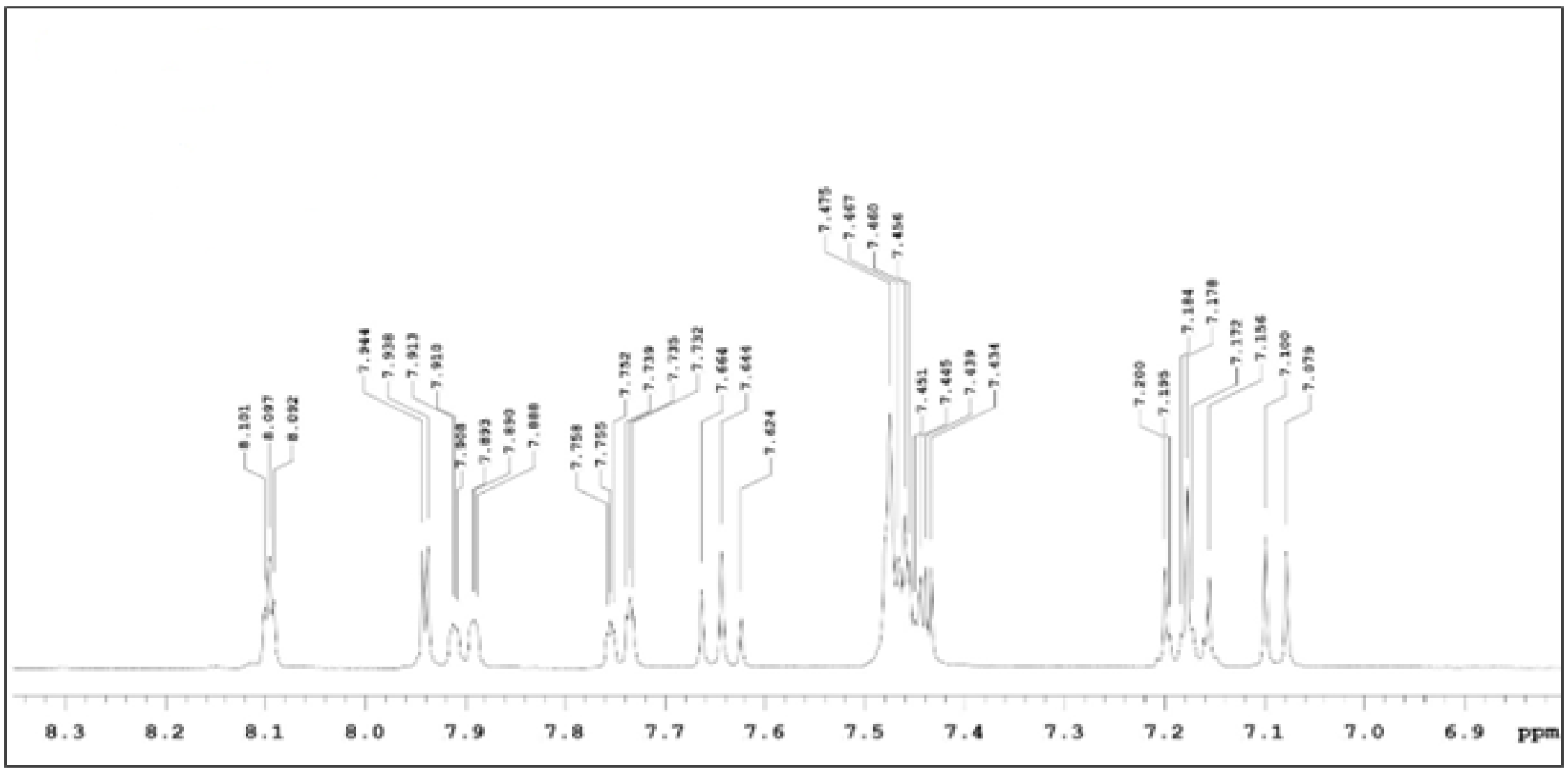

Figure S33: ^{1}H NMR of compound **6j** (6.9 – 8.3 ppm)

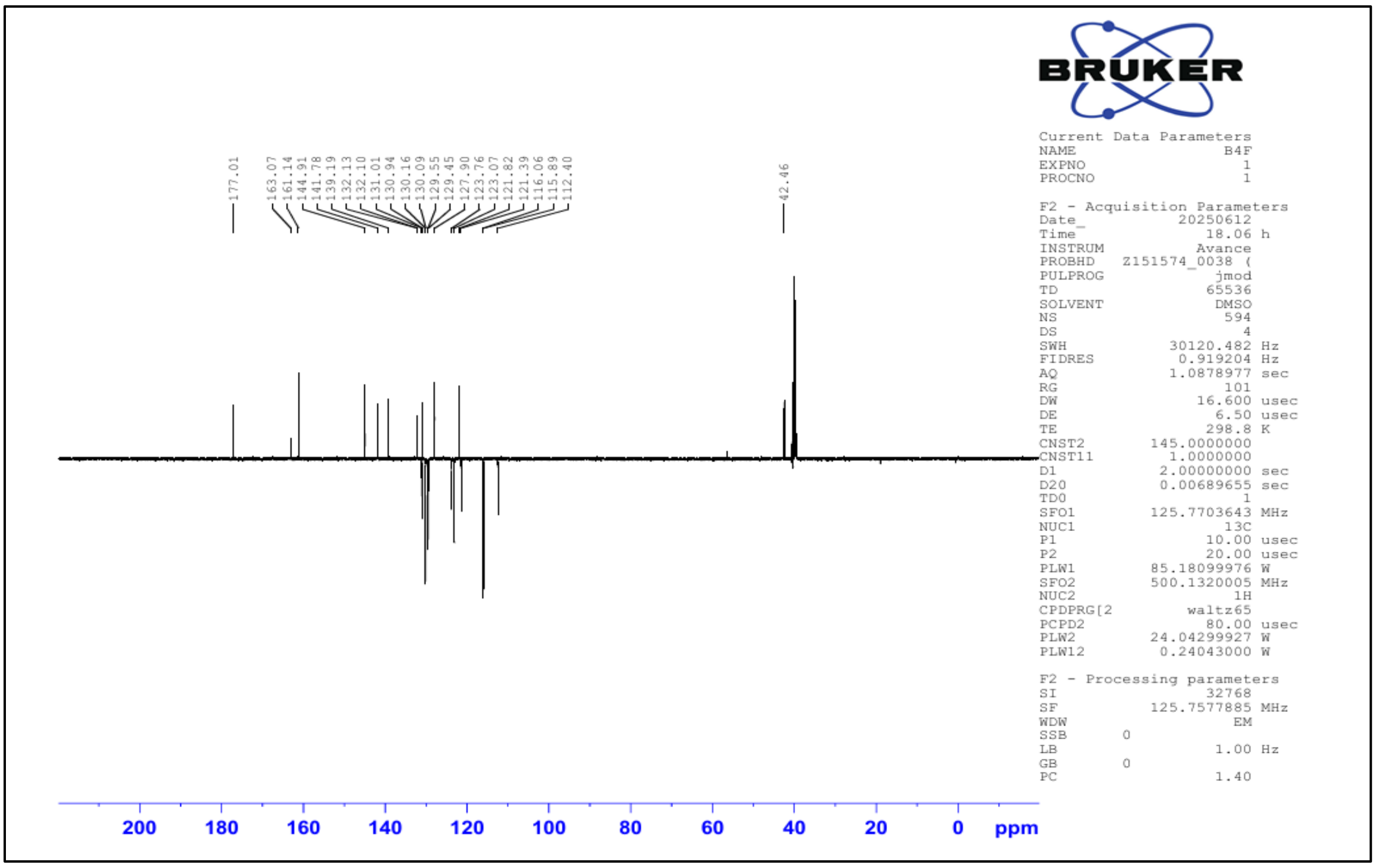

Figure S34: ^{13}C NMR of compound **6j** (APT)

1-(3-Chlorobenzyl)-5-chloro-2-oxoindolin-3-ylidene)-4-(3-sulfamoylphenyl)thiosemicarbazide (6k)

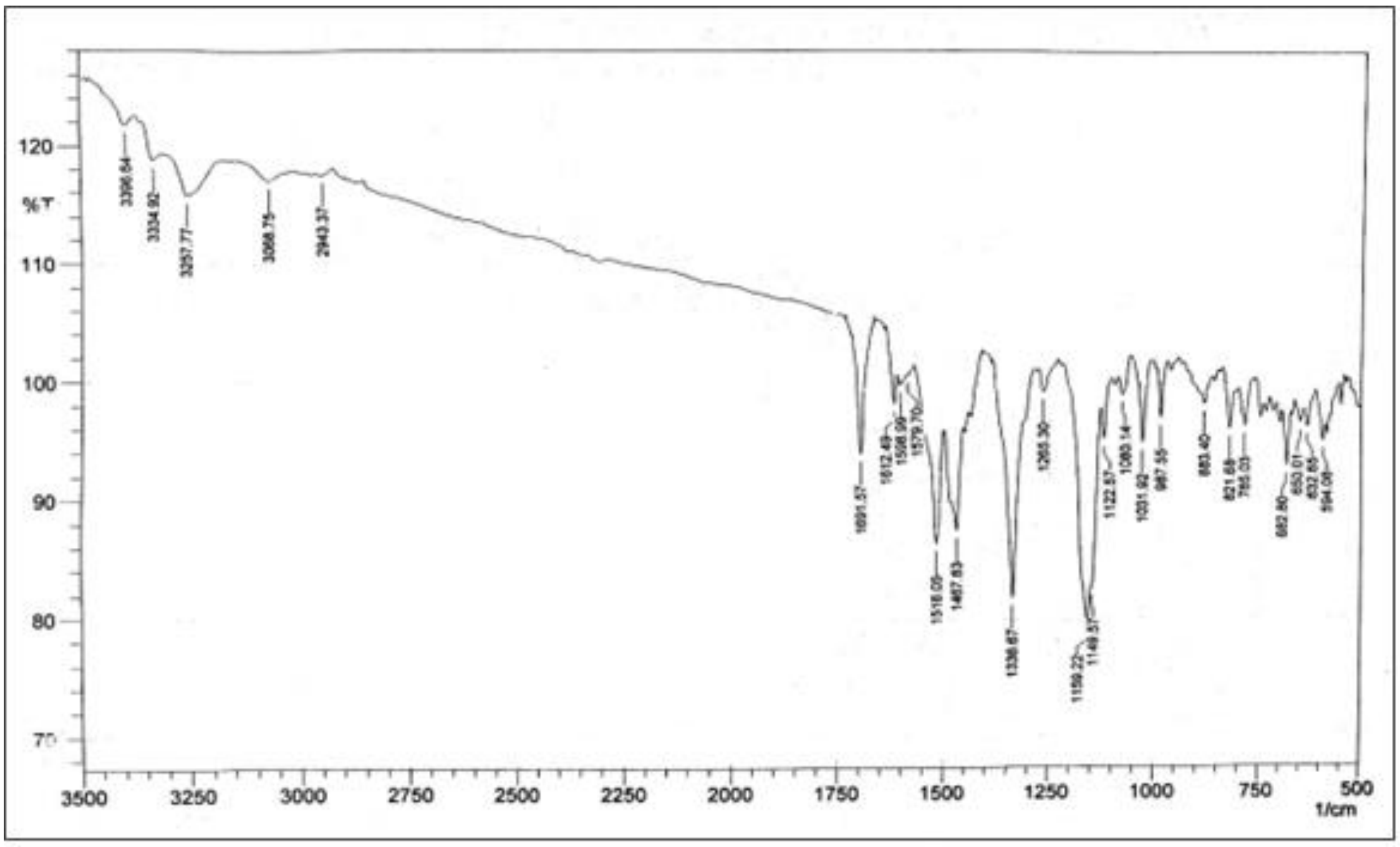

Figure S35: IR spectrum of compound **6k**

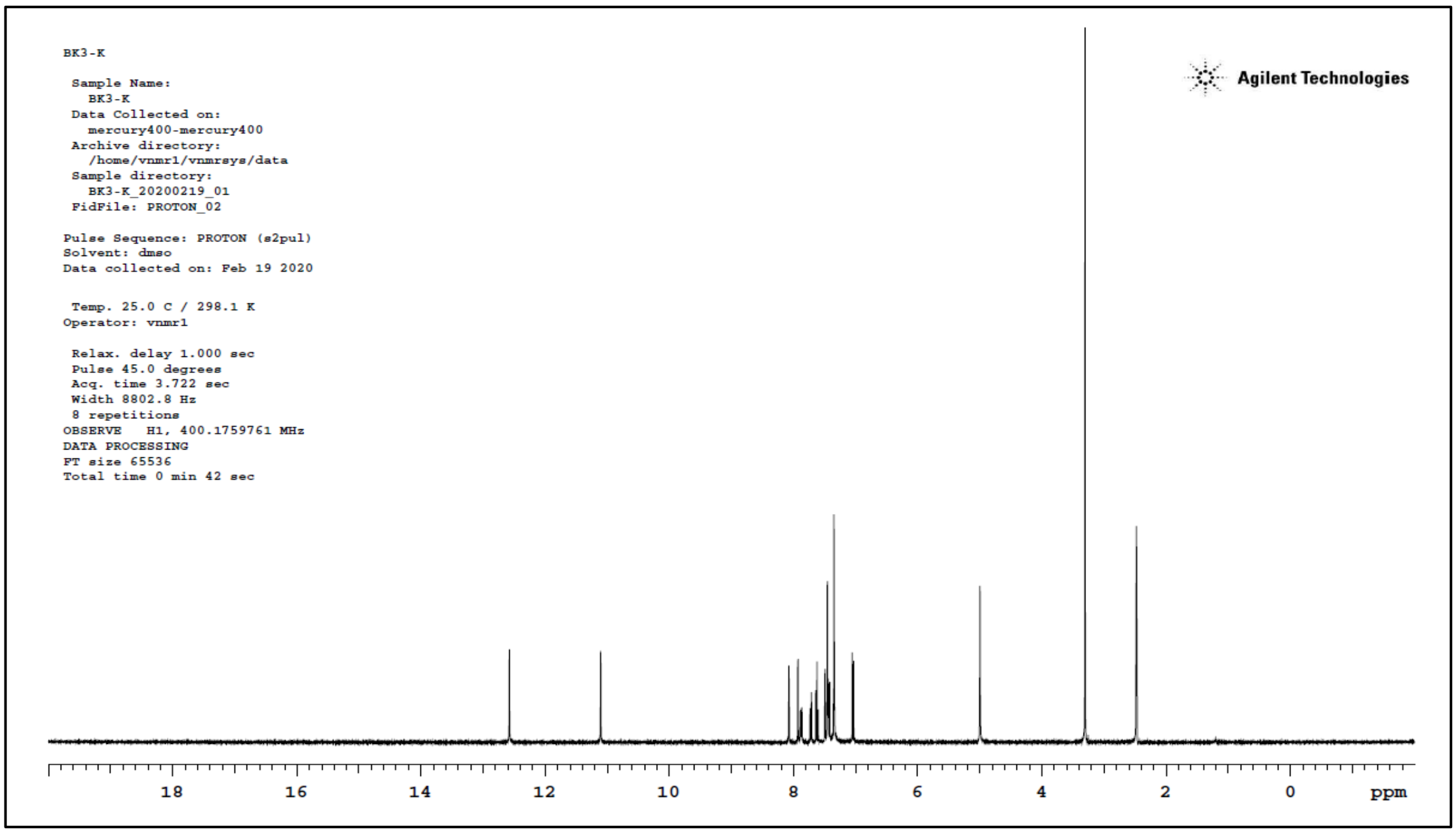

Figure S36: ^{1}H NMR of compound **6k**

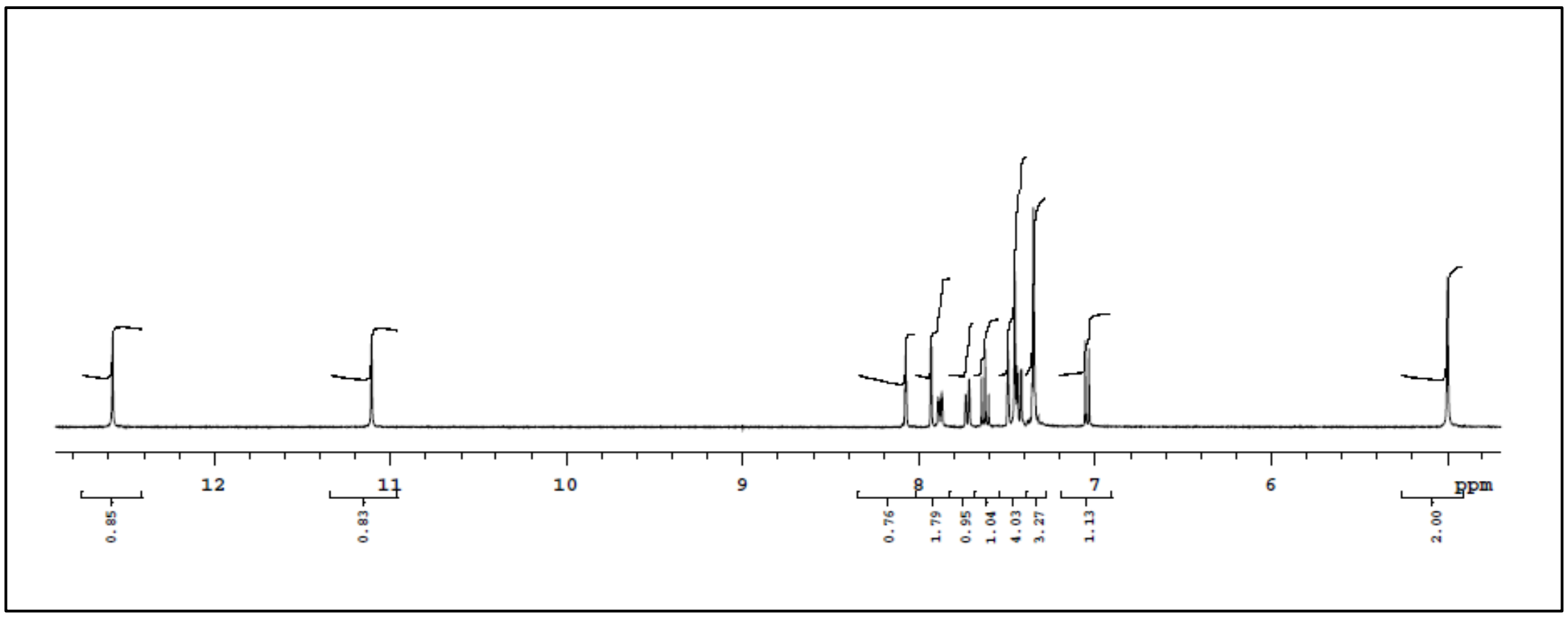

Figure S37: ^{1}H NMR of compound **6k** (5.5 – 13.0 ppm)

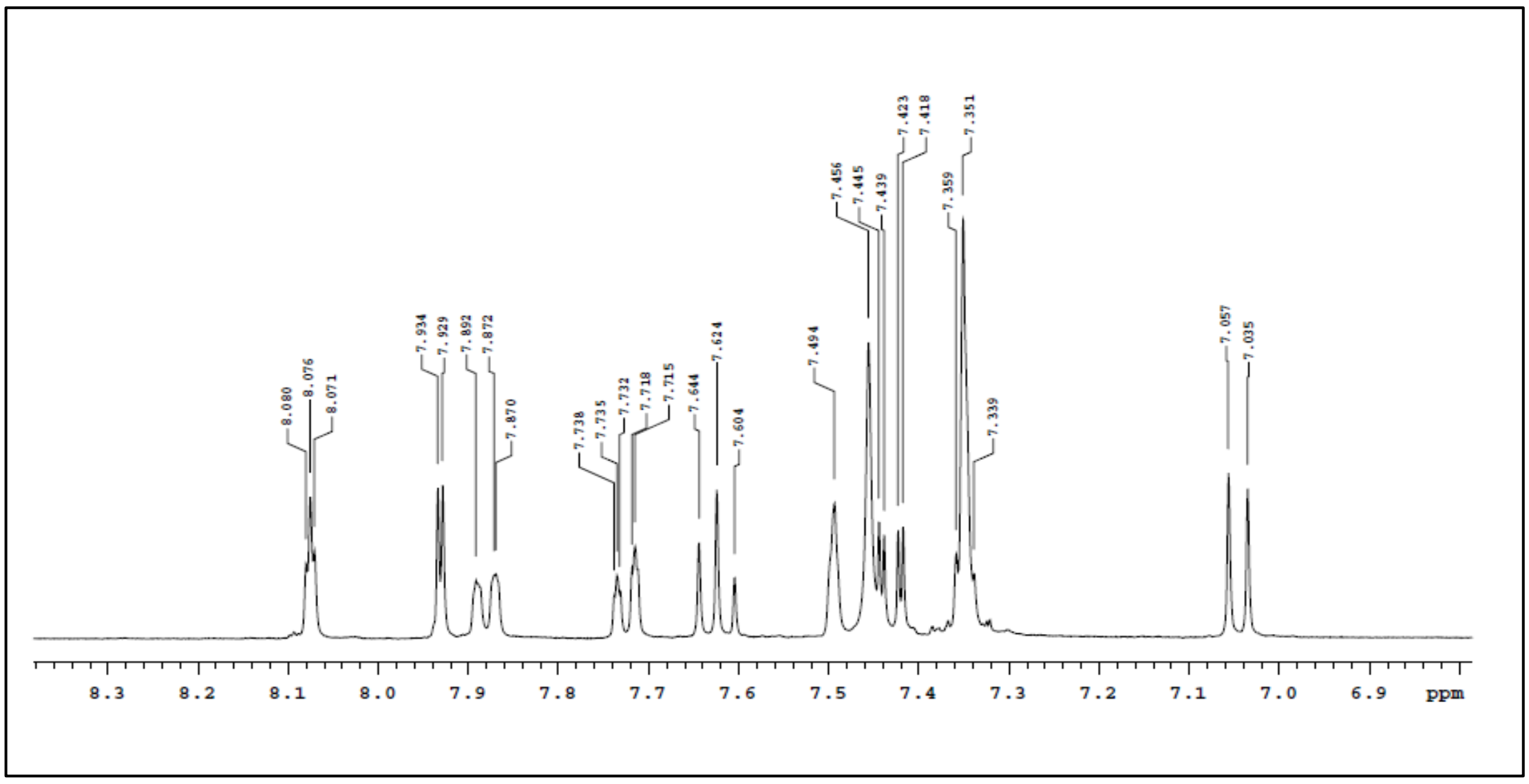

Figure S38: ^{1}H NMR of compound **6k** (6.9 – 8.3 ppm)

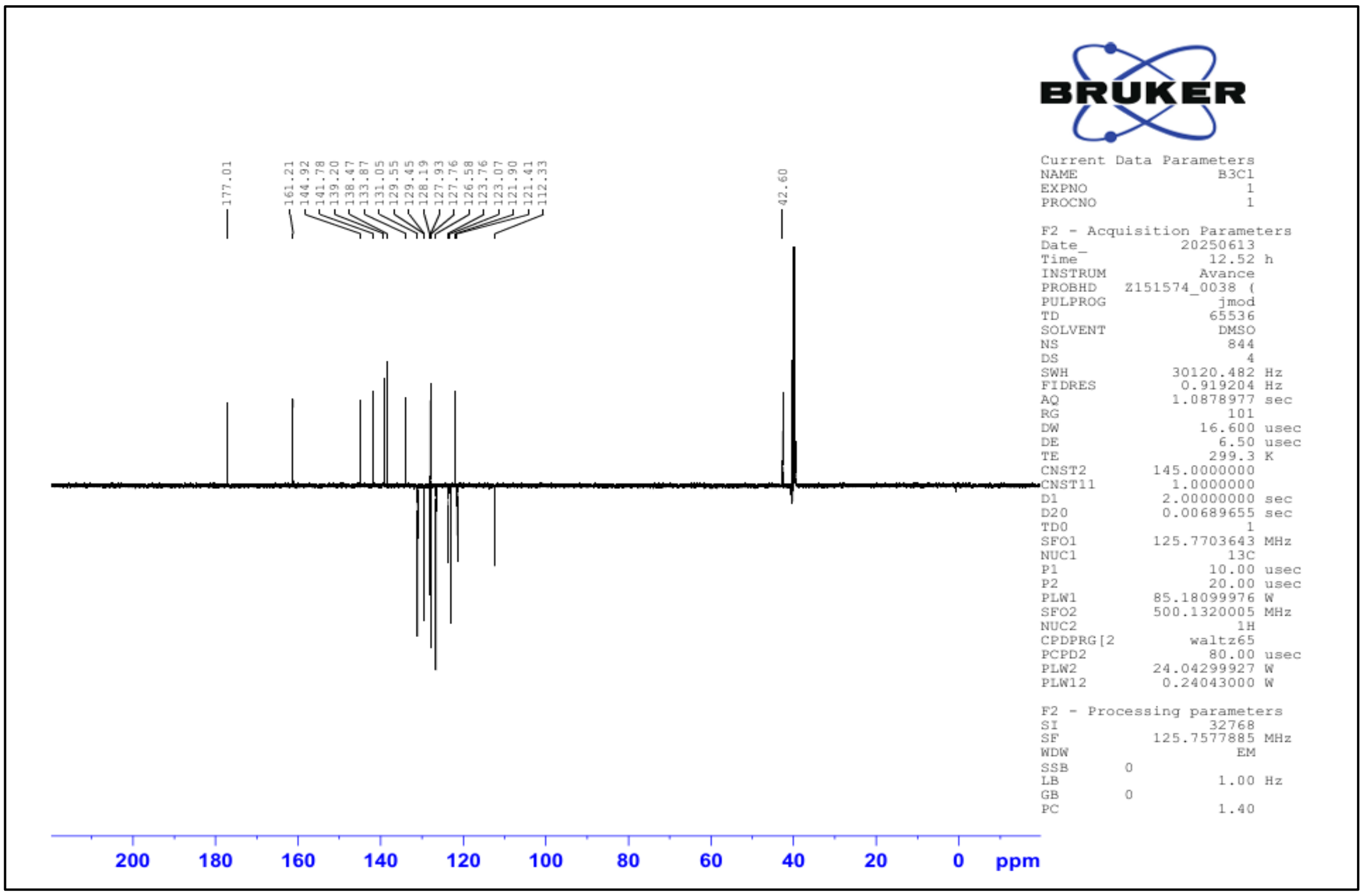

Figure S39: ^{13}C NMR of compound **6k** (APT)

1-(4-Chlorobenzyl)-5-chloro-2-oxoindolin-3-ylidene)-4-(3-sulfamoylphenyl)thiosemicarbazide (6l)

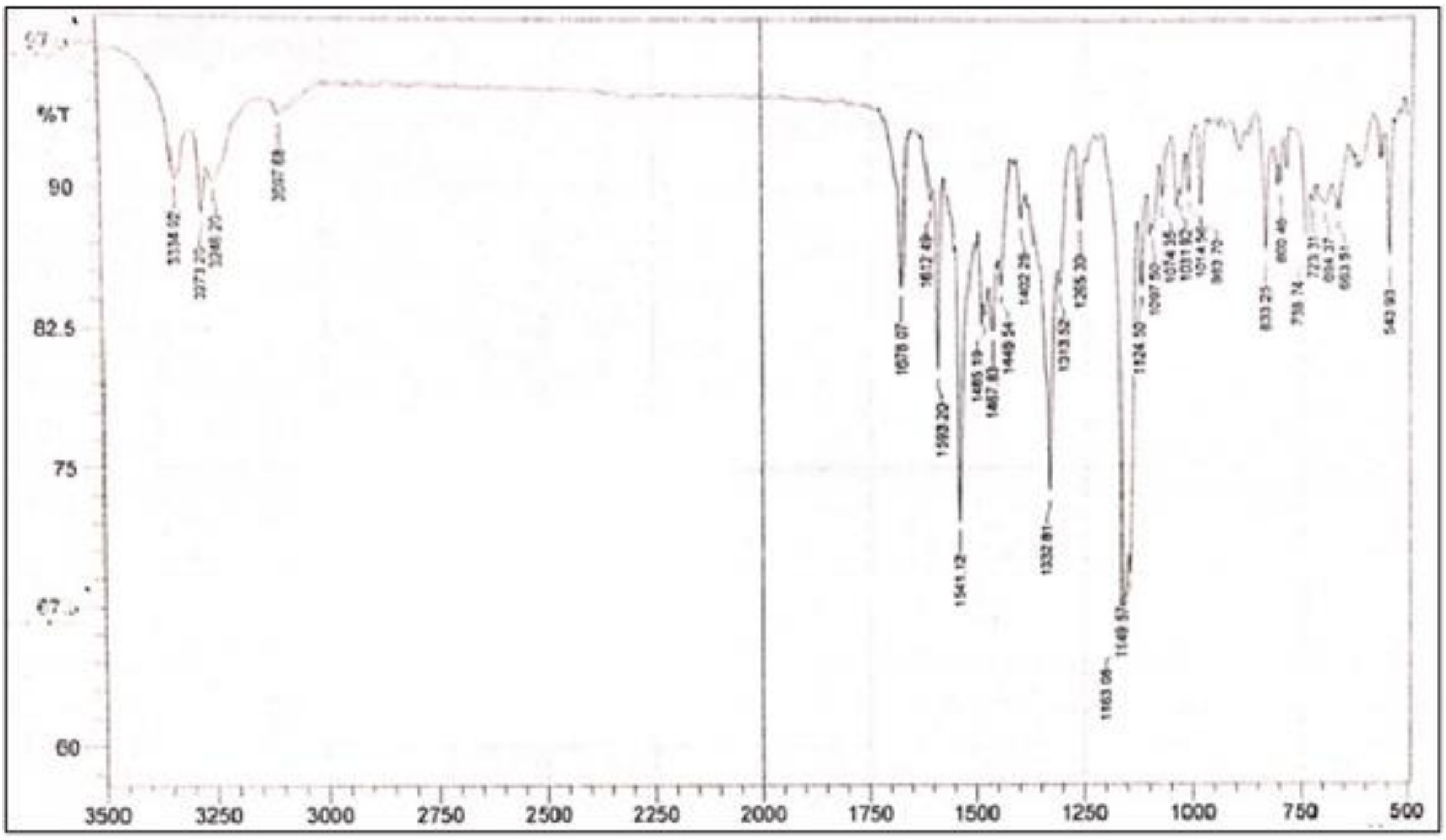

Figure S40: IR spectrum of compound **6l**

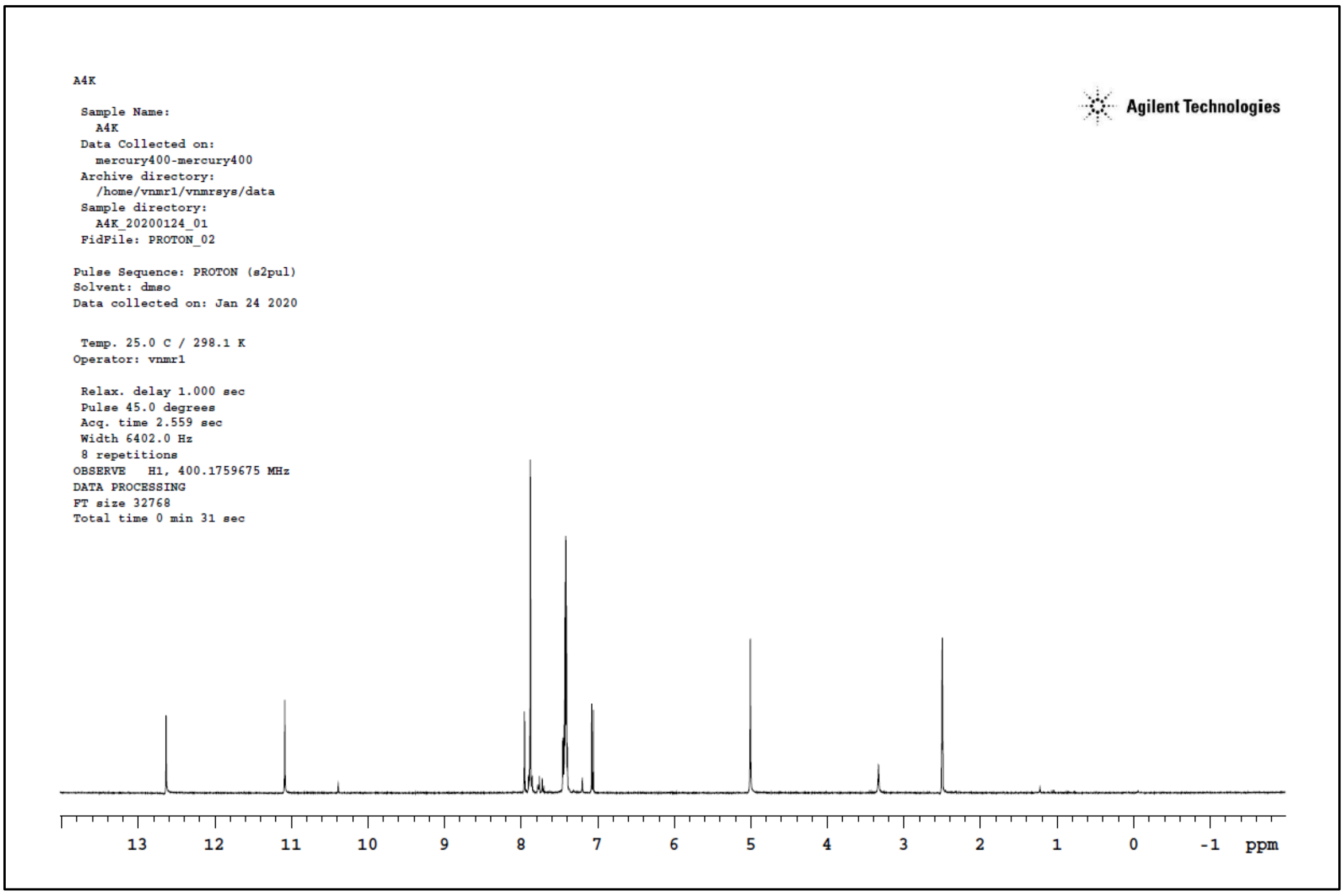

Figure S41: ^{1}H NMR of compound **6l**

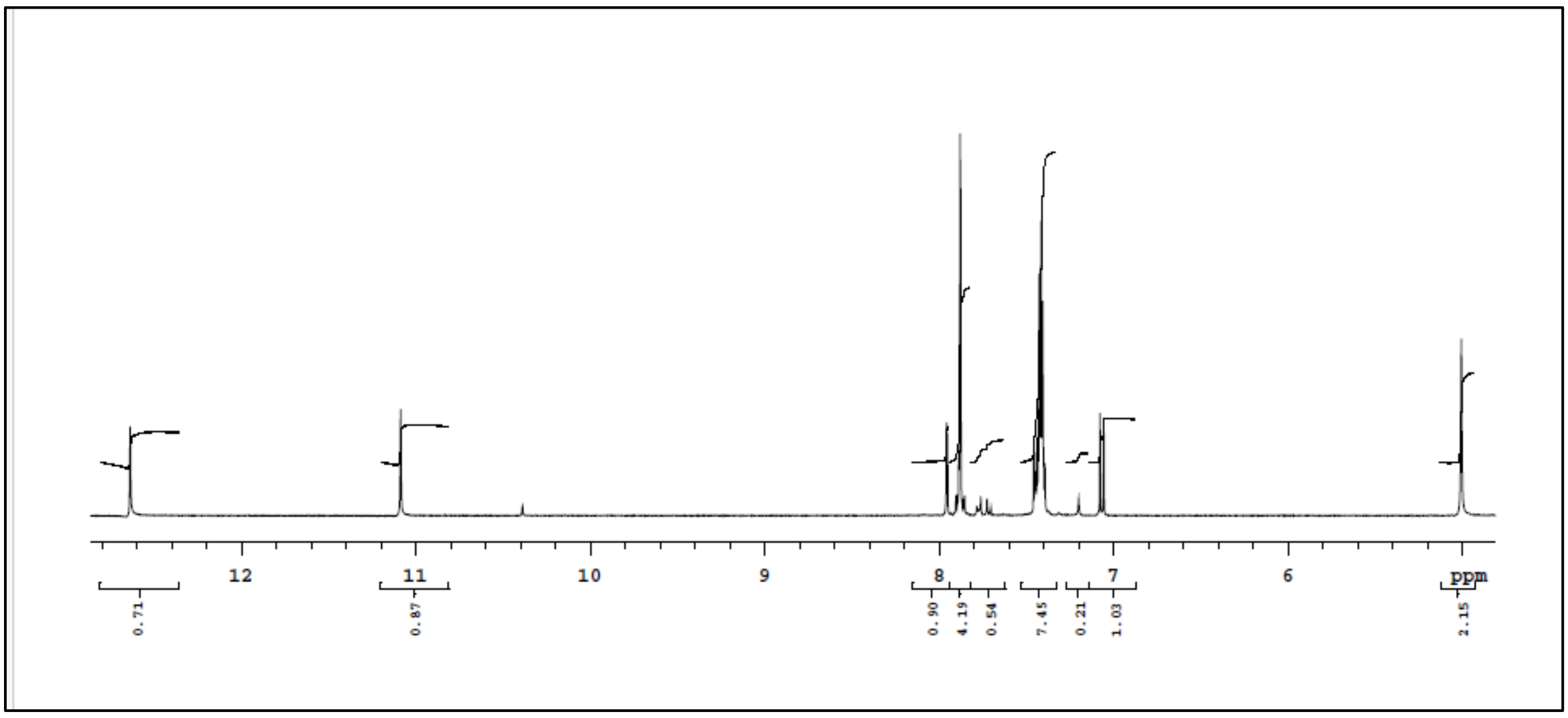

Figure S42: ^{1}H NMR of compound **6l** (5.0 – 13.0 ppm)

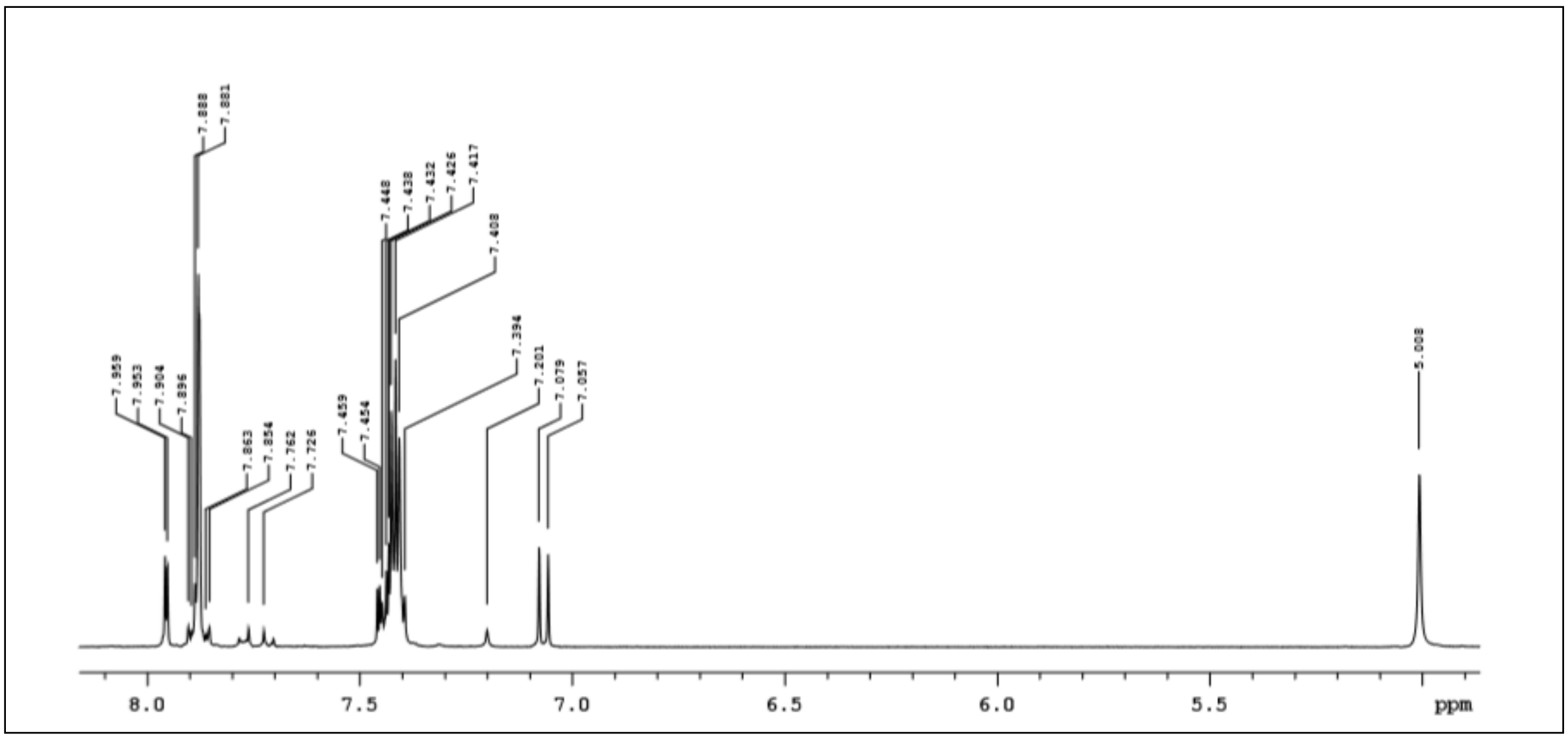

Figure S43: ^{1}H NMR of compound **6l** (5.0 – 8.0 ppm)

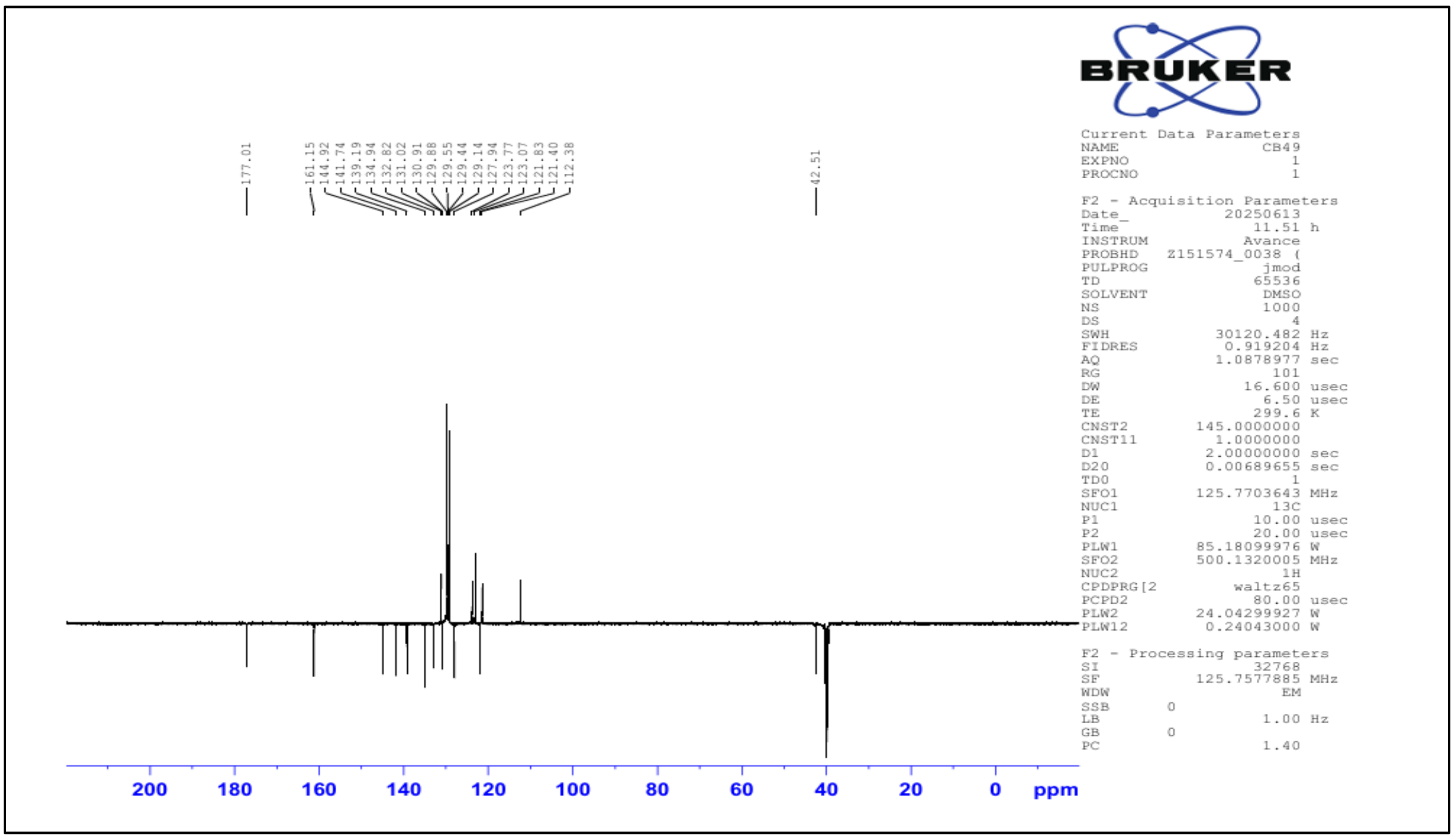

Figure S44: ^{13}C NMR of compound **6l** (APT)
